# Conservation of NLRP3 Inflammasome Pathway in Monotremes and Large-Scale Restructuring of the Caspase-1 Gene Cluster Region in Mammals

**DOI:** 10.1007/s00239-026-10307-6

**Published:** 2026-03-12

**Authors:** David Stevens, Tasman Daish, Frank Grützner

**Affiliations:** School of Biological Sciences, Adelaide University, Adelaide, SA 5005 Australia

**Keywords:** Inflammasome, Phylogenetics, Mammalian gene evolution, Comparative Immunology/Evolution, Gene Rearrangement, Inflammation

## Abstract

Activation of the NLRP3 inflammasome can be triggered by components of fungi, bacteria and viruses, as well as cellular stress and environmental irritants. The NLRP3 inflammasome has been well characterised in mouse and humans but limited information is available from other mammalian species. To gain a better understanding of the evolution of genes involved in the NLRP3 inflammasome pathway, we examined them in mammalian species representing the three major lineages (eutheria, metatheria and prototheria) and in chicken as an outgroup. Our results show that the inflammasome pathway machinery is generally well conserved in the species examined. We identified four NLRP members in echidna and seven in platypus as well as confirming *Nlrp3* is present in marsupials and monotremes. Monotremes feature eleven Dectin family genes that are split across two chromosomes. Only three family members were found in opossum, Tasmanian devil and koala. Of the four Dectin family members known to be involved in the NLRP3 inflammasome pathway only *Clec4e* (*Mincle*) was identified in all species examined. Echidna possesses a single copy of Caspase-1 which, alongside previous results reported in the platypus, supports the conclusion that this is the only proinflammatory caspase in the monotremes. Our analysis suggests that *Caspase-1* moved to a new chromosomal region in early mammalian evolution. This was followed by expansion of the cluster and accumulation of additional genes. The expansion of key gene families flanking *Caspase-1* may have led to an expansion of inflammasome pathways and a more regulated immune system through the CARD genes.

**Supplementary Information:**

The online version contains supplementary material available at 10.1007/s00239-026-10307-6.

## Introduction

**Fig. 1:**
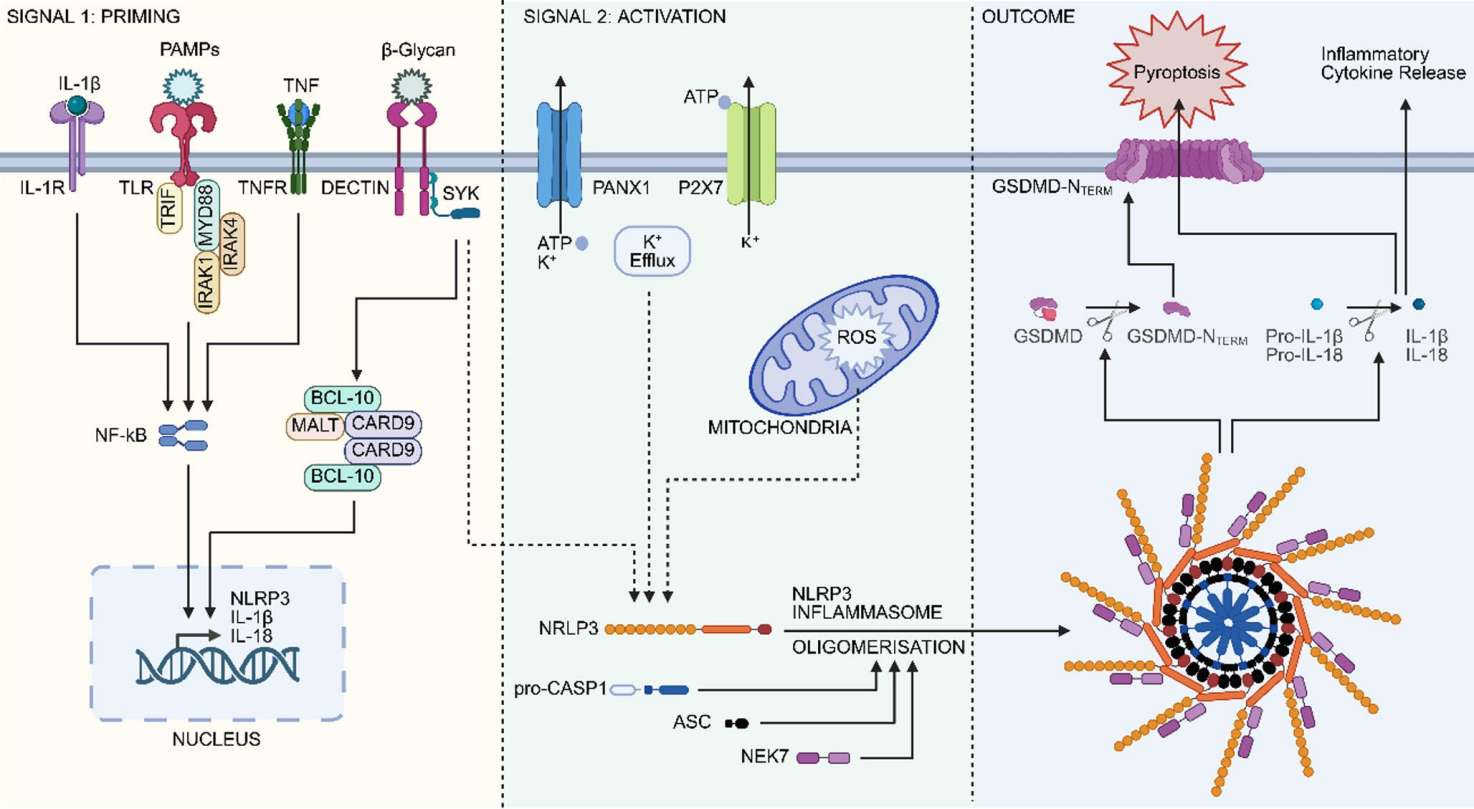
Molecular activation of the NLRP3 inflammasome. Activation canonically occurs through two signals: priming and activation. The priming signal (left) occurs between cytokines or pathogen-associated molecular patterns (PAMPs) and the receptors that detect them. This initiates an upstream signalling cascade that upregulates the expression of NLR family, pyrin domain containing 3 (NLRP3) inflammasome components. The activation signal (centre) is induced by a variety of PAMPs and damage-associated molecular patterns (DAMPs) which leads to activation of upstream events such as K^+^ efflux and production of reactive oxygen species (ROS) in the mitochondria. These result in the formation of the inflammasome and the cleavage of pro-Caspase-1 (pro-CASP1) (right). The active Caspase-1 is then able to cleave pro-IL-1β, pro-IL-18 and Gasdermin D (GSDMD) to their active forms. IL-1β and IL-18 are released from the cell and GSDMD-N_TERM_ form pores in the cell membrane inducing pyroptosis. IL-1R, IL-1 receptor; TLR, Toll-like receptor; TNF, Tumour necrosis factor; TNFR, Tumour necrosis factor receptor; Syk, Spleen tyrosine kinase; TRIF, TIR-domain-containing adaptor-inducing interferon-β (TICAM1); MyD88, Myeloid differentiation primary response gene (88); IRAK1, Interleukin-1 receptor associated kinase 1; IRAK4, Interleukin-1 receptor associated kinase 4; NF-κΒ, Nuclear factor-κΒ; BCL-10, B-cell CLL/lymphoma-10; MALT1, Mucosa associated lymphoid tissue lymphoma translocation gene 1; CARD9, Caspase recruitment domain family, member 9; PANX1, Pannexin 1; P2X7, P2X Purinergic receptor 7; ATP, Adenosine triphosphate; ROS, reactive oxygen species; ASC, PYD and CARD domain containing (PYCARD); NEK7, NIMA related kinase 7

Inflammasomes are multiprotein complexes, which generally consist of a sensor and an adaptor serving as a scaffold that promotes Caspase-1 autocatalytic cleavage and processing (Martinon et al. 2002). Inflammasomes are known to play essential roles in the host response to pathogen-associated molecular patterns (PAMPs) and damage-associated molecular patterns (DAMPs). There have been several inflammasomes described to date, several of which are formed by Nod-like Receptor (NLR) family members: NLR family, pyrin domain containing 1 (NLRP1) (Martinon et al. 2002), NLRP3 (Agostini et al. 2004), NLRP6 (Leng et al. 2020), NLRP7 (Khare et al. 2012; Radian et al. 2015), NLRP9 (Zhu et al. 2017), NLRP10 (Zheng et al. 2023), NLRP12 (Ataide et al. 2014) and NLRC4 (Mariathasan et al. 2004) as well as the HIN-200 family member AIM2 (Bürckstümmer et al. 2009; Fernandes-Alnemri et al. 2009; Hornung et al. 2009), IFI16 (Singh Vivek et al. 2013) and MFEV (Pyrin) (Akbaba et al. 2021; Xu et al. 2014). The NLRP3 inflammasome has been shown to form in response to a variety of stimuli including PAMPs such as lipopolysaccharide (LPS) and DAMPs such as ATP (Dostert et al. 2008; Gao et al. 2022; Gross et al. 2009; Kanneganti et al. 2006; Martinon et al. 2006; Rodrigues et al. 2020; Zeng et al. 2022). Here we investigate the evolution of genes involved in pathways relating to gram-negative bacteria (Kanneganti et al. 2006) and fungi (Gross et al. 2009) (Fig. 1).

Caspases are a class of cysteine proteases which cleave proteins with caspase-specific target motifs following an aspartate residue (Bibo-Verdugo and Salvesen 2024; Cerretti et al. 1992). Generally, caspases are ubiquitously expressed and exist as inactive zymogens (termed pro-caspases) which contain a prodomain and sites involved in cleavage and dimerization. Caspases are considered initiators of apoptosis (caspases − 2, −8, −9 and − 10), effectors of apoptosis (caspases-3, −6 and − 7) or proinflammatory proteins (caspases-1, −4, −5, −11, −12 and − 13) (Degterev et al. 2003; Eckhart et al. 2008). The proinflammatory caspase subfamily is also referred to as the *Caspase-1* subfamily as all the members are in close proximity in a cluster on the same chromosome. Gene duplication has shaped diversity of the caspase genes where *Caspase-11* and *Caspase-12* are likely to be the result of duplications of *Caspase-1* and *Caspase-5* a duplication of *Caspase-4* (Eckhart et al. 2008). While the main function of the proinflammatory caspases is to promote the inflammatory response, there is evidence that certain members are also capable of effecting apoptosis (Mandal et al. 2018; Shao et al. 2021; Tsuchiya et al. 2019; Wang et al. 1998). *Caspase-12* appears to be unique in that it acts as a negative regulator in mouse while in human it is an inactive pseudogene (Fischer et al. 2002; Saleh et al. 2006).

Knowledge of the molecular and biochemical mechanisms underlying the inflammasome response is increasing but information on the evolution of the pathway is still limited to human and mouse systems. The Caspase-1 subfamily has undergone gene expansion during evolution (Eckhart et al. 2008), associated with increased complexity of the inflammatory response. *Tumour Necrosis Factor* (*TNF*) (Glenney and Wiens 2007; Roca et al. 2008) and the *Toll-like Receptors* (*TLR*s) (Alvarez-Pellitero 2008) are present in multiple species outside of mammals but knowledge of the other inflammasome pathway genes in other species is very limited. NLR family members have been examined extensively in rodents and primates (Hughes 2006; Pétrilli and Martinon 2011; Tian et al. 2009) with further work expanding this to include dog, cow and chicken (Hughes 2006; Tian et al. 2009). Platypus (using the OANA5 assembly) and opossum reproductive NLRPs have been examined using genbank sequences (Duenez-Guzman and Haig 2014). Their combined works show species specific expansions for several of the NLRP members as well as deletion of certain members in some lineages. A recently published work on Gasdermin D (GsdmD) describes the evolution of the gene family in therian mammals and non-mammalian species using human, gorilla, mouse, cow, pig, seal and whale to represent the mammalian lineages (Angosto-Bazarra et al. 2022).

Monotremes are the most basal lineage of living mammals and the recently released echidna genome and the improved platypus genome assemblies provides an opportunity to assess the evolution of genes involved in the NLRP3 inflammasome pathway in mammals (Zhou et al. 2021, 2025). Comparative analysis with the examined therians shows that the majority of the genes involved in the NLRP3 inflammasome pathways are conserved in the three mammalian lineages. Our data suggests an expansion of key gene families (proinflammatory caspases, NLRP and Dectin) may have led to the development of the non-canonical inflammasome pathways, providing alternate pathways to induce a response, as well as an increase the regulatory elements through the CARD genes.

## Materials and Methods

A combination of BLAST, synteny and phylogenetic analysis was utilised to determine the conservation status of the different inflammasome pathway components in multiple mammalian species and chicken (Table 1). tBLASTn searches were used to identify potential homologues in the unannotated monotreme assemblies (platypus (mOrnAna1) and echidna (mTacAcu1) using human (GRCh38.p14) protein sequences as the query (Online Resource 1). The region surrounding the BLAST results were extracted and GENSCAN (Burge and Karlin 1997, 1998) used to predict putative genes in that region. The precited protein sequences were then used in tBLASTn searches against the human RNA Refseq library to determine likely homologues. Putative platypus and echidna sequences can be found in Online Resource 2 while the BLAST results can be found in Online Resource 3.

Multiple therian species were also examined using tBLASTn with human protein sequences as a query against the annotated NCBI GenBank assemblies. These were chimpanzee (NHGRI_mPanTro3-v2.1_pri), mouse (GRCm39), rat (GRCr8), dog (UU_Cfam_GSD_1.0), cow (ARS-UCD2.0), opossum (mMonDom1.pri), Tasmanian devil (mSarHar1.11) and koala (phaCin_unsw_v4.1). Chicken (bGalGal1.mat.broiler.GRCg7b) was selected as an outgroup for most of the genes examined. For *Pycard*, the green anole lizard (GCF_035594765.1-RS_2024_02) was chosen as the outgroup. The protein sequences were used in tBLASTn against the human RNA Refseq library (Online Resource 4). Synteny analysis was performed using eutherian chromosome alignments as references.

Predicted protein sequences from examined species were aligned in MEGAX (Kumar et al. 2018) using MUSCLE (Edgar 2004). Neighbour-joining (NJ) phylogenetic trees were generated using the p-distance method with 1000 bootstrap repeats and pairwise deletion. MEGA12 was used to identify the best model for the generation of Maximum likelihood trees using partial deletion with a 95% site coverage cut-off (Kumar et al. 2024; Nei and Kumar 2000; Tamura et al. 2011; Yang 1994). The resulting tables can be found in Online Resources 5, 6, 7 and 8. Maximum likelihood (ML) trees were generated using the model with the lowest Bayesian Information Criterion (BIC). The phylogenetic trees were edited in MEGA12 (Kumar et al. 2024). The ExPASy Prosite tool (de Castro et al. 2006; Sigrist et al. 2002, 2005, 2013) was used to predict protein domains.

**Table 1:** NLRP3 inflammasome pathway genes in multiple mammalian species and chicken

	GGA	TAC	OAN	MDO	SHA	PCI	BTA	CFA	RNO	MMU	PTR	HSA
CASP1	+	+	+	+++	+	+	+	f	+	+	++	+(*p*)
CASP4/11/13	-	-	-	+	+	+	+	f	+	+	+	+(p)
CASP5	-	-	-	-	-	-	-	-	-	-	+	+
CASP12	-	-	-	++	+++	+(p)	-	+	-	-	+	+
NLRP3	+	+	+	+	+	+	+	+	+	+	+	+
PYCARD	-	+	+	+	+	+	+	+	+	+	+	+
NEK7	+	+	+	+	+	+	+	+	+	+	+	+
GSDMD	-	+	+	+	+	+	+	+	+	+	+	+
IL-1Β	+	+	+	+	+	+	+	+	+	+	+	+
IL-18	+	+	+	+	+	+	+	+	+	+	+	+
CLEC7A	-	+	++	-	-	-	+	+	+	+	+	+
CLEC6A	-	-	-	-	-	-	+	-	-(p)	+	+	+
CLEC4D	-	+	+	-	-	-	+	+	+	+	+	+
CLEC4E	-	+	+	+	+	+	+	+	+	+	+	+
SYK	+	+^	+^	+	+	+	+	+	+	+	+	+
TNF	+	+	+	+	+	+	+	+	+	+	+	+
MYD88	+	+	+	+	+	+	+	+	+	+	+	+
CARD9	+	+	+	+	+	+	+	+	+	+	+	+
BCL10	+	+	+	+	+	+	+	+	+	+	+	+
MALT1	+	+	+	+	+	+	+	+	+	+	+	+
PANX1	+	+	+	+	+	+	+	+	+	+	+	+
P2X7	+	+	+	+	+	+	+	+	+	+	+	+

+: present, -: absent, f: fusion described previously (Eckhart et al. 2008). p: pseudogene/truncated, and ^: needs to be confirmed through experimental analysis

‘GGA’ Gallus gallus (chicken), ‘TAC’ Tachyglossus aculeatus (Echidna), ‘OAN’ Ornithorhynchus anatinus (Platypus), ‘MDO’ Monodelphis domestica (Opossum), ‘SHA’ Sarcophilus harrisii (Tasmanian devil), ‘PCI’ Phascolarctos cinereus (Koala) ‘BTA’ Bos taurus (Cow), ‘CFA’ Canis lupus familiaris (Dog), ‘RNO’ Rattus norvegicus (Rat), ‘MMU’ Mus musculus (Mouse), ‘PTR’ Pan troglodytes (Chimpanzee), ‘HSA’ Homo sapiens (Human)

## Results

### Evolution of Inflammasome Pathway Components in Vertebrates

Generally, we found that the inflammasome pathway is evolutionarily conserved in the species examined (Table 1, Online Resource 3 and Online Resource 4). However, the analysis also revealed unexpected changes to several components of the NLRP3 inflammasome pathway (Table 1).

### Dectin Family Receptors

*Dectin*−1 (*Clec7a*), *Dectin-2* (*Clec6a*), *Dectin-3* (*Clec4d*) and *Mincle* (*Clec4e*) belong to a group of immunoreceptor tyrosine-based activation motif (ITAM)-containing or ITAM-coupled C-type lectins known as the Dectin family (Goodridge et al. 2011; Malamud and Brown 2024; Plato et al. 2013). These receptors are essential in humans and mice for the activation of Syk-dependent and independent pathways in response to fungi (Gross et al. 2006; Sato et al. 2006; Steele et al. 2005; Zhao et al. 2014; Zhu et al. 2013). The members of the Dectin family are located within the natural killer cluster in two different clusters. The Dectin-1 cluster consists of *Dectin-1 *(*Clec7a*), *Clec1* (*Clec1a*), *Clec2* (*Clec1b*), *DNGR1* (*Clec9a*), *Micl* (*Clec12a*), *Mah* (*Clec12b*) and *Lox1* (*Olr1*) (Online Resource 9a). The Dectin-2 cluster consists of *Dectin-2 (Clec6a)*, *DCIR* (*Clec4a*), *DCAR* (*Clec4b1*), *BDCA-2* (*Clec4c*), *Mincle* (*Clec4e*) and *Dectin-3* (*Clec4d*) (Online Resource 9b). Some species-specific variations have been previously reported; *Clec4c* is only found in humans and chimpanzees, while *Clec4b1/2* is specific to rodents (Malamud and Brown 2024).

Our analysis shows that in monotremes the Dectin family genes are unexpectedly split across two chromosomes where the Dectin-1 cluster co-locates with the natural killer cluster (Chr17 in platypus and scaffold_11 in echidna) while the Dectin-2 cluster is located on ChrX4 in platypus and scaffold_55 in echidna (Online Resource 3).

For both monotremes, the Dectin-1 cluster contains eight genes while the Dectin-2 cluster has only three. These genes have been labelled as *Clec-like* with 1–8 covering those on platypus Chr17 and echidna scaffold_11 and 9–11 for the genes located on platypus ChrX4 and echidna scaffold_55.

In most eutherians examined the Dectin-1 cluster contains 7 genes with six in dog (Online Resource 4). The genes show similar organisation on chromosomes except from human and chimpanzee which have flipped *Clec1b* and *Clec12b* (Online Resource 9a).

Our data suggests that opossum, Tasmanian devil and koala genomes lack *Clec7a* and possess only two members of the Dectin-1 cluster (Online Resource 9a and Online Resource 10). The results are more complicated in monotremes. Phylogenetic analysis suggests the platypus *Clec-like6* and *Clec-like7* are homologues of *Clec7a* (Online Resource 10). The NJ tree predicts echidna *Clec-like7* to be *Clec7a* (Online Resource 10b) while the ML tree places it in a cluster that diverged prior to the *Clec12a* and *Clec1a* clusters (Online Resource 10a). Based on the agreement between the BLAST data and NJ tree, along with the poor bootstrap support in the ML tree, we are identifying echidna *Clec-like7* as a *Clec7a* homologue.

The second cluster in monotremes is flanked by *Necap* and *C3ar1* which share synteny with the Dectin-2 cluster in eutherians supporting initial BLAST results suggesting the three genes belong to this cluster (Online Resource 9b). Phylogenetic analysis shows that platypus and echidna *Clec-like 11* are homologues of *Clec4e* (Online Resource 10). *Clec4e* was also identified in the three marsupial species. Both trees indicate that platypus *Clec-like10* and echidna *Clec-like10* are homologous. The NJ tree suggests the monotreme *Clec-like10*s are *Clec4d* while the ML tree suggests it diverged prior to the *Clec4d* and *Clec6a* clusters. Based on these data we are identifying monotreme *Clec-like10* as *Clec4d*. No homologues for *Clec6a* or *Clec4d* were found in marsupials. In fact, *Clec4e* was the only member of the Dectin-2 cluster identified in opossum, koala and Tasmanian devil suggesting marsupials have lost most of the Dectin family. Our data shows dog lacks a *Clec6a* homologue while rat contains a *Clec6a* pseudogene. The remaining monotreme Dectin family members are discussed further in Supplementary Material 3.

### Spleen Tyrosine Kinase (Syk) in Monotremes

Spleen tyrosine kinase (*Syk*) is involved in one of the Dectin-1 (Clec7a) signalling pathways (Gringhuis et al. 2009). SYK activates NF-κΒ resulting in the upregulation of several cytokines (Gringhuis et al. 2009; LeibundGut-Landmann et al. 2007) and is involved in NLRP3 inflammasome activation in response to the fungi *C. albicans* and *S. cerevisiae* (Gross et al. 2009).

*Syk* was identified in chicken and all therian mammals examined. In the OANA5 platypus assembly *Syk* was absent with only a partial sequence present which appeared more similar to *Zap70*, a member of the same tyrosine kinase family. We re-examined this result with the new assemblies to determine if this result was accurate.

**Fig. 2:**
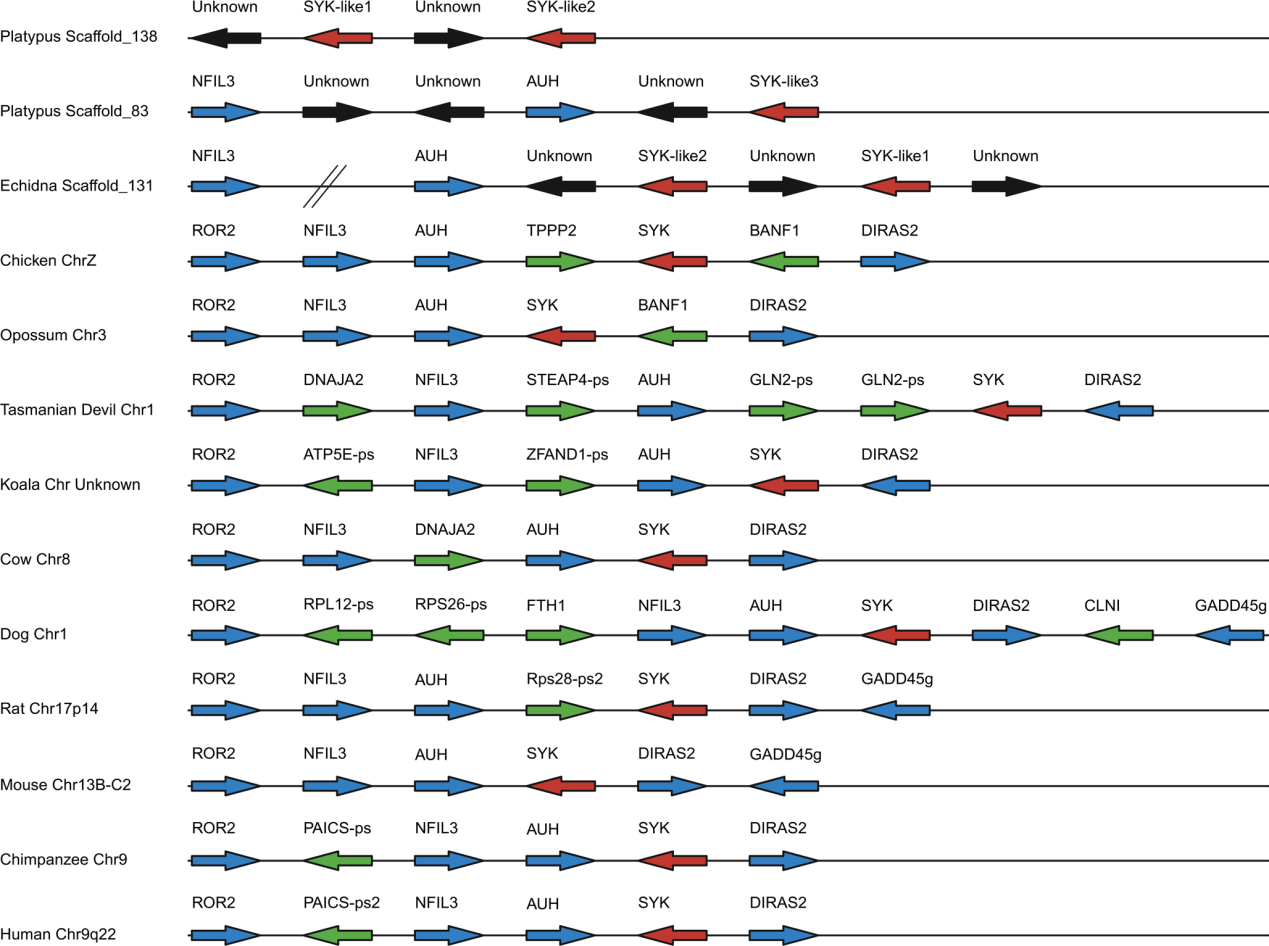
Chromosomal arrangements of Syk in multiple mammalian species with chicken as non-mammalian outgroup. Arrows indicate orientation. Red arrows represent the gene of interest, blue represents syntenic genes while green represents those that are species specific and black arrows are putative genes identified by GENSCAN that lack an apparent homologue in human. -ps at the end of a gene name indicates a pseudogene. Distances are not to scale

In platypus three potential *Syk* were identified (Fig. 2). Two of the genes (*Syk-like1* and *Syk-like2*) are located next to each other (scaffold_138) while the third (*Syk-like3*), was found on scaffold_83. The flanking genes *Auh* and *Nfil3* were also identified on scaffold_83 along with *Syk-like3*. *Diras2* is located on chr18 while *Ror2* and *Gadd45g* are absent.

The first match for *Syk* in echidna was to scaffold_1 however BLAST results suggested the gene was *Zap70*. This was further supported by conserved synteny with *Adamts10* and *Myo1f* as observed in therians (Online Resource 11). *Tmem131* was found on scaffold_33.

The second match in the echidna genome was to scaffold_131 where two copies of *Syk* were identified (Fig. 2). *Syk-like1* is flanked downstream by *Auh* and *Nfil3* which is consistent with *Syk* in therians. *Syk-like2* is located upstream of *Syk-like1* with an unknown gene separating them. *Diras2* was identified on scaffold_17 while *Gadd45g* appears to be absent.

Platypus Syk-like1 and echidna Syk-like2 contain the two Src homology 2 (SH2) domains found in SYK and ZAP70 however they lack the protein kinase domain. Conversely platypus Syk-like2 and Syk-like3, and echidna Syk-like1 contain the protein kinase domain but lack the SH2 domains. The arrangement of the monotreme Syk-like genes on the scaffolds matches the domain order observed in the therian sequences. As such we predict that these are the result of an assembly issue and they represent a single Syk gene in monotremes.

### NLRP3 is Conserved in Amniotes

**Fig. 3:**
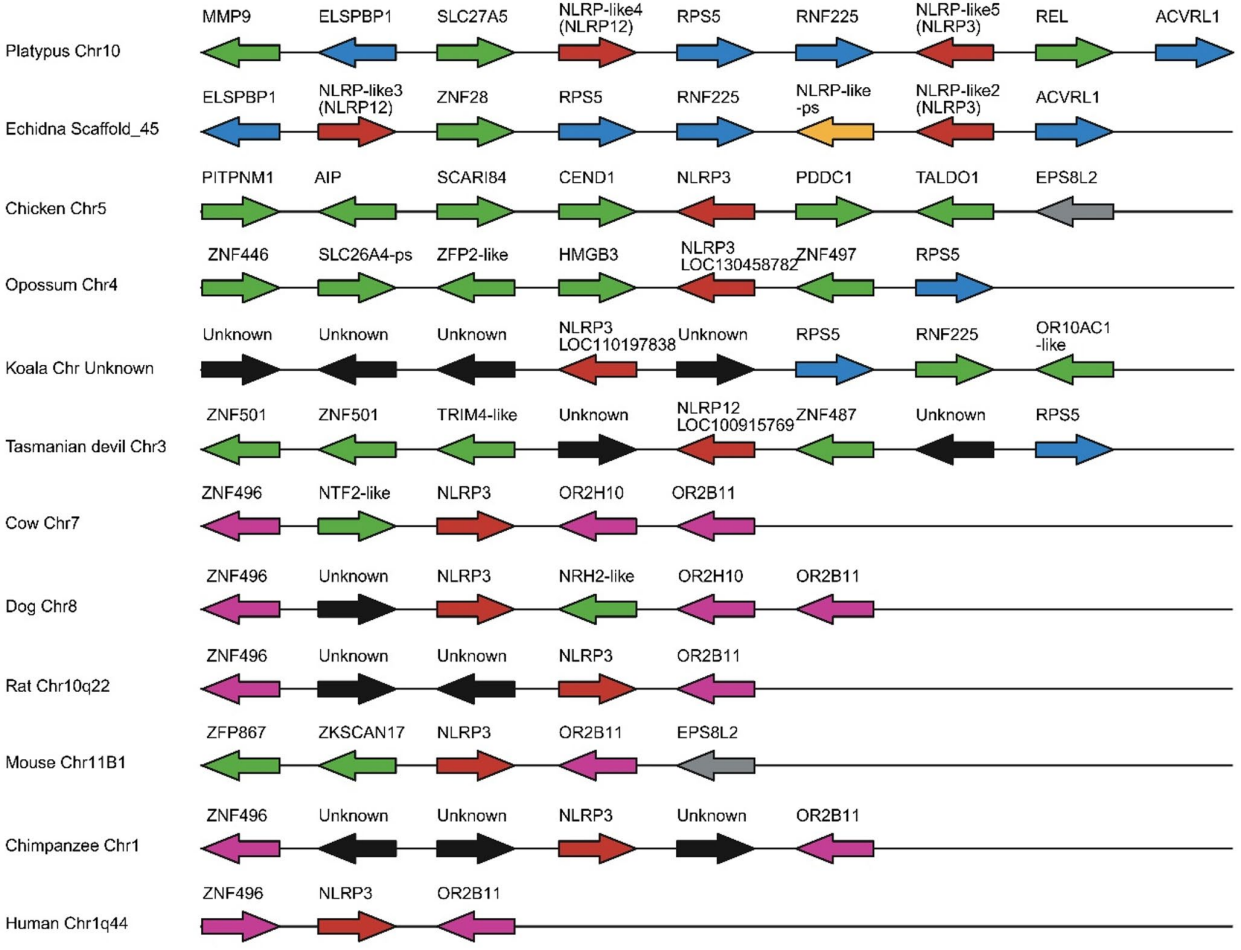
Chromosomal arrangements of *Nlrp3* for multiple mammalian species and chicken as an outgroup. Arrows indicate orientation. Red arrows are genes of interest, blue, pink and dark grey are conserved between species, green arrows are species specific and black arrows are putative genes with no apparent orthologue. The yellow arrow represents a putative Nlrp-like pseudogene in echidna. -ps at the end of a gene name indicates a pseudogene. Distances not to scale

NLRP3 functions as the inflammasome scaffold through NACHT domain interactions (Mariathasan et al. 2006). The LRR sequence of the NLRs is thought to function as a PAMP sensor and the interaction of these PAMPs with the LRR region is thought to trigger dimerization and inflammasome formation (Kanneganti et al. 2007). Like the caspases, the NLRP genes appear to be divided in to two roles: reproduction and inflammation (Tian et al. 2009). A previous study has examined this family in the platypus and opossum where they were unable to identify a *Nlrp3* homologue in opossum (Duenez-Guzman and Haig 2014). We decided to re-examine the conservation of the NLRPs in the new monotreme and marsupial assemblies to further elucidate the evolution of these genes (Fig. 3 and Online Resources 12–17).

Seven *NLRP-like* genes were identified in the platypus assembly and four in echidna. Two potential homologues of *Nlrp3* were identified in platypus (*Nlrp-like5/Nlrp3* and *Nlrp-like3*) and one in echidna (*Nlrp-like2/Nlrp3*) (Online Resource 17). While both ML tree (Online Resource 17a) and NJ tree (Online Resource 17b) agree on platypus *Nlrp-like5* and echidna *Nlrp-like2* the NJ tree suggests platypus *Nlrp-like3* is *Nlrp10*. The lack of LRR suggests platypus *Nlrp-like3* is most likely a *Nlrp10* duplicate as this is the only known NLRP member to lack the LRR motif (Wang et al. 2004). The monotreme *Nlrp3* share synteny with each other and are found in proximity to the monotreme *Nlrp12* homologues (platypus *Nlrp-like4* and echidna *Nlrp-like3*) (Fig. 3). Platypus *Nlrp-like3* is flanked by *Nlrp-like2* (*Nlrp10*) (Online Resource 14). No putative homologues were identified in the syntenic region of echidna. Homologues of *Nlrp3* were also identified in opossum, Tasmanian devil and koala. Marsupial *Nlrp3* only shares synteny with *Rps5* which was also observed to flank *Nlrp3* in monotremes. The eutherian species examined showed synteny with *Znf496* and *Or2b11*. Based on these data we believe that *Nlrp3* is present in all three mammalian lineages and that it has relocated several times. The first prior to monotreme divergence, the second prior to marsupial divergence and the third after marsupial divergence. The remaining monotreme NLRP are discussed in more detail in Supplementary Material 3.

### Potential Loss of PYCARD in Birds

PYCARD (ASC) is an adaptor protein that contains both a CARD and a pyrin (PYD) domain, allowing it to interact with the inflammasome sensor proteins and pro-Caspase-1. Recruitment and oligomerisation of PYCARD is essential for the maturation of Caspase-1 in inflammasomes formed by sensor proteins that lack a CARD such as NLRP3 (Mariathasan et al. 2004). In inflammasomes with CARD containing sensor proteins a second PYCARD-independent pathway has been observed (Broz et al. 2010). NLRP1 is able to interact directly with pro-Caspase-1, however, processing of Caspase-1 in the NLRP1 inflammasome is greatly increased when PYCARD is present (Faustin et al. 2007).

**Fig. 4:**
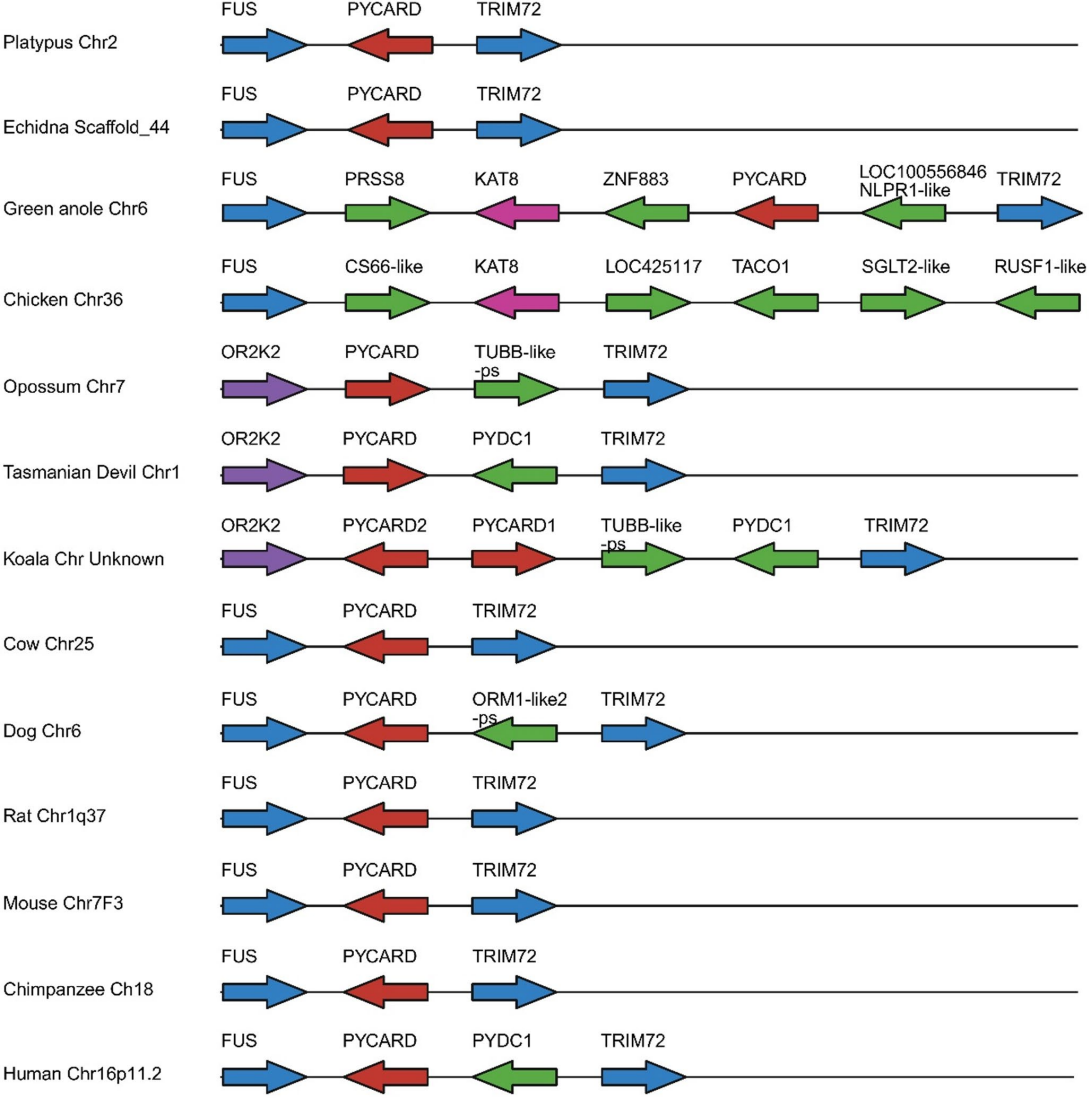
Chromosomal arrangements of *PYCARD* in multiple mammalian species with chicken and green anole lizard as non-mammalian outgroups. Anole LOC100556846 Nlrp1-like is named according to NCBI gene identification. It contains a PYD and FIIND lacking the NACHT, CARD and LRR of NLRP1. Arrows indicate orientation. Red arrows represent the gene of interest, blue, purple and pink represent syntenic genes while green represents those that are species specific. -ps at the end of a gene name indicates a pseudogene. Distances are not to scale

*Pycard* was identified in all species examined except for chicken. In monotremes *Pycard* can be found with the same flanking genes as those in therian mammals (Fig. 4). In platypus there appears to be another *Pycard-like* gene on chr2 however it contains only part of the second exon of *Pycard*. Two copies of *Pycard* were identified in the koala genome flanking each other and sharing synteny with the other marsupials. Both are predicted to have the necessary PYD and CARD domains for a functional protein. Phylogenetic analysis suggests the monotreme Pycard are more closely related to the eutherian Pycard than the marsupial proteins are (Online Resource 18). To investigate whether the absence in chicken is common to the bird lineage we searched the zebrafinch and turkey genomes for *Pycard* but were unable to identify a homologue. A *Pycard* homologue was identified in the green anole where the flanking genes *Fus* and *Trim72* were also found.

### Gasdermin Conservation in Mammals

Gasdermin D (GSDMD) has been implicated in pyroptosis, an inflammatory caspase dependant cell death pathway (Fernandes-Alnemri et al. 2007; Liu et al. 2016, 2024). Cleavage of Gasdermin D leads to the generation of an active N-terminal cleavage product that is then able to oligomerize and create pores in the membranes of cells leading to cell death (Liu et al. 2016). *GsdmD* was identified in all therians examined. However, in chicken and green anole the closest match is to Gasdermin A (GSDMA). This result was supported by synteny (Online Resource 19). In monotremes, *GsdmD* was found on chr4 for platypus and scaffold_29 in echidna where they show conserved synteny with therian *Gsdmd*. Interestingly both Tasmanian devil and koala have a second *GsdmD-like* gene flanking *GsdmD* indicating a duplication has occurred in these species. The positions of these *GsdmD-like* genes suggest they may be the results of species-specific duplications (Online Resource 19).

### Rearrangement and Relocation of Caspase-1 Gene Cluster

Facilitating Caspase-1 maturation and activation is the key function of the inflammasome. Caspase-1 maturation leads to the processing of IL-1β and IL-18 which are essential effectors of the inflammatory response. *Caspase-1* orthologues were readily identified in the platypus and echidna genome assemblies. Phylogenetic analysis confirmed this result with the monotreme caspases forming a clade near the therian Caspase-1 (Online Resource 20). Dog Caspase-1 is more divergent and either clustered with eutherian Caspase-1 (Online Resource 20a) or with eutherian Caspase-4 (Online Resource 20b). Interestingly Tasmanian devil LOC100914126 Caspase-4b is in a clade with the marsupial Caspase-12 suggesting these evolved from a common ancestor. This suggests that the Tasmanian devil has one copy each of Caspase-1 and Caspase-4 and three copies of Casapse-12 (LOC100914126, LOC100913862 and LOC100913330) (Table 1 and Online Resource 21). The koala LOC110215005 Caspase-4b forms a clade with the other marsupial Caspase-1 suggesting this is a Caspase-1 homologue (Table 1 and Online Resource 21). A Caspase-12 pseudogene (Caspase-12p) was identified in koala with shared synteny with the other inflammatory caspases (Table 1 and Online Resource 21). Interestingly the koala Caspase-1 cluster has an unusual organisation with the Caspase-4 homologue first followed by Caspase-1, then Caspase-12p and Caspase-12 (Online Resource 21). Based on these data we believe that monotremes contain only a single member of the proinflammatory caspases (Caspase-1) while the Tasmanian devil and opossum appear to have undergone several species-specific duplications.

The genes flanking the Caspase-1 subfamily in mammals appear to be well conserved (Online Resource 21). The chicken genome assembly contains a single orthologue of *Caspase-1*. Comparison of the genes flanking mammalian *Caspase-1* with those in chicken revealed that the gene resides in entirely different locations in mammalian and reptile genomes (Online Resource 21). The mammalian *Caspase-1* flanking regions have conserved synteny in chicken albeit without *Caspase-1* (Online Resource 21). Indeed, the syntenic genes in chicken can be found in the same order in eutherian mammals except that genes upstream of chicken *Caspase-1* are found grouped on a separate chromosome to those that were located downstream. In monotremes the chicken *Caspase-1* syntenic genes are still located on the same chromosome however the upstream gene cluster and downstream gene cluster are not syntenic with each other suggesting rearrangements to this region had occurred prior to monotreme divergence. The chromosomal relocation of these syntenic blocks then occurred after marsupial divergence. These observations suggest that a transposition event has occurred early in mammalian evolution that resulted in relocation of *Caspase-1* and later, the chromosomal rearrangement of the region.

## Discussion

### Evolution of the NLRP3 Inflammasome

The immune response to external challenges is a vital characteristic of multicellular organisms. Many of the genes involved in the NLRP3 inflammasome pathway are highly conserved in the mammalian species examined (Table 1, Online Resource 3 and Online Resource 4) while others display extensive change. In this study we aimed to shed light on the evolution of the NLRP3 inflammasome pathway and the region containing *Caspase-1*. The conservation of some of these genes is likely due to their function in fundamental cellular processes other than the immune response. For example, Nf-κΒ is involved in apoptosis (Ryan et al. 2000) and neuronal function (Freudenthal et al. 2005; Levenson 2004; Meffert et al. 2003).

Our data shows the core components of the NLRP3 inflammasome are conserved with *Nek7*, *Nlrp3*, *Pycard* and *Caspase-1* present in species representing the three major mammalian lineages. The improvements to the opossum assembly have allowed us to identify a *Nlrp3* homologue where it was previously thought absent (Duenez-Guzman and Haig 2014) and we have confirmed the gene is also present in the Tasmanian devil and koala. We observed frequent species-specific losses and duplications in the monotreme and marsupial NLRP family members which are discussed in Supplementary Material 3.

It is possible that the duplication of *Pycard* in the Tasmanian devil is the result of the proinflammatory caspase expansion in the species. The koala also features a duplication of *Pycard* yet contains a single copy of *Caspase-1*, *Caspase-4* and *Caspase-12*. Pycard has roles in apoptosis (Ohtsuka et al. 2004) so perhaps these duplications allow for another level of regulation to both the inflammatory response and the apoptotic pathways. This may, in part, explain why the marsupial Pycard appears to be more divergent than the monotreme homologues (Online Resource 18). *Pycard* is missing in chicken, zebrafinch and turkey but we found an orthologue in the green anole indicating it is present in reptiles.

### Pyroptosis Outside of Eutherians

It has been shown that mutating *GsdmD* at key residues prevented the proteins from oligomerizing and blocked pyroptosis (Liu et al. 2016). This demonstrates that GsdmD is essential for pyroptosis in mouse and human. The absence of GsdmD in chicken suggests that for pyroptosis to occur another gene would have to take on this role and this seems to be the case. The Gasdermin family member, Gasdermin A (GsdmA), is present in chicken and is capable of inducing autophagy through its N-terminal while the C-terminal acts to inhibit it, similar to the behaviour of the GsdmD cleavage products (Shi et al. 2015). Recent work has demonstrated that infectious bronchitis virus triggers the NLRP3 inflammasome, upregulates expression of GsdmA and GsdmE and induces pyroptosis in chicken (Han et al. 2025). The presence of *GsdmD* in monotremes suggests the canonical pathways to induce pyroptosis are conserved (Fig. 1), however, the absence of *Caspase-4/−11* suggests the noncanonical pyroptosis pathway may be lost or altered compared to human and mouse (Kayagaki et al. 2015).

### Conservation of the NLRP3 Inflammasome Fungal Response Pathways in Mammals

The Dectin family is located next to NKC genes and, like the NKC genes, are c-type lectins. While the NKC has undergone massive expansions in monotremes (Warren et al. 2008; Zhou et al. 2021) the Dectin family has not. We identified eleven Dectin family genes in monotremes compared to 13 in humans. In the eutherian species examined the Dectin family appears to be mostly conserved. However, in the marsupial species examined only three family members were identified (*Clec1a*, *Clec1b* and *Clec4e*) indicating a significant gene loss in these species. Phylogenetic analysis suggests that monotremes experienced species-specific duplications. We confirmed *Clec4e*, one of the Dectin family members involved in the NLRP3 inflammasome pathway, is present in the three mammalian lineages. We have identified two homologues for *Clec7a* in platypus (*Clec-like6* and *Clec-like7*) and one in echidna (*Clec-like7*). We have also identified a *Clec4d* homologue in the monotremes (*Clec-like10* in both platypus and echidna). Confirming the identity of some of the monotreme Dectin family members is complicated due to how divergent they are. This likely accounts for the low bootstrap support and the disagreement between the two phylogenetic methods. This could be exacerbated by the limited number of closely related taxa for the monotremes, and the limited number of genes present in marsupials. This results in phylogenetic trees comparing monotremes with the more distant eutherian sequences. The Dectin family members respond to a variety of PAMPs (Malamud and Brown 2024) and our data suggests that this response may be impaired in the monotremes and particularly marsupials. There is also evidence of crosstalk between family members and the absence of genes, such as *Dectin-2* (*Clec6a*) in monotremes, dog, rat and the marsupials examined may increase susceptibility for responses that require both proteins (Chang et al. 2017; Haider et al. 2019; Roesner et al. 2019). Dectin family members not involved in the NLRP3 inflammasome pathway are covered in Supplementary Material 3.

The apparent duplication of *Syk* in the monotremes is interesting. These genes appear to be truncated versions of *Syk* and domain prediction suggests that the necessary domains are split across the duplicates. The absence of Syk in monotremes would be very unexpected as it plays a crucial role, not just in the NLRP3 inflammasome pathway but also B cell receptor signalling and in T cell receptor β-selection (Palacios and Weiss 2007). Duplications may serve as an extra layer of redundancy or regulation as there is some evidence of Syk phosphorylation of two tyrosine residues in Pycard to enhance its ability to recruit and bind with pro-Caspase-1 (Lin et al. 2015). However, our results suggest that monotremes contain a single copy of *Syk* with an assembly error incorrectly splitting a single gene in two.

Together this suggests that the three mammalian lineages can induce the NLRP3 inflammasome in response to fungal threats although the variety of threats the monotremes and marsupials are capable of responding to may be reduced compared to eutherians.

### Evolution of the Mammalian Proinflammatory Caspase Chromosome Region

The *Caenorhabditis elegans* cell death gene *ced3* is thought to be the orthologue of the caspase family and it functions, like most caspase family members, in programmed cell death (PCD) (Yuan et al. 1993). Caspase-1, however, is involved predominantly in promoting inflammation although it can also cleave apoptotic targets under specific conditions suggesting that Caspase-1 has acquired its inflammatory function over evolutionary time (Miura et al. 1993; Wang et al. 1998). Indeed data suggests that Caspase-1 is able to induce apoptosis in cells that lack GsdmD (Tsuchiya et al. 2019). Based on Caspase-1 homologues in zebrafish, gilt-head seabream and Xenopus, this change in function has occurred after the divergence of *Actinopterygii* (Lopez-Castejon et al. 2008; Masumoto et al. 2003). Interestingly, Caspase-a has an essential role during zebrafish development and requires zebrafish PYCARD for activation (Masumoto et al. 2003). Caspase-1, however, has no such role in mammalian development (Li et al. 1995). Furthermore, in place of the Caspase recruitment domain (CARD) found in the prodomain of the mammalian *Caspase-1*, the *Caspase-a* and *Caspase-b* prodomain contains a pyrin domain (PYD) and the interaction between Caspase-a and zebrafish PYCARD is through the PYD (Masumoto et al. 2003).

An amino acid change in the cysteine-active site of chicken Caspase-1 raises doubts as to its ability to cleave its substrates (IL-1β and IL-18) to their active states and for inactivating IL-33 (Johnson et al. 1998). Furthermore, chicken IL-1β is lacking the Caspase-1 consensus site necessary for activation (Gyorfy et al. 2003). Interestingly a study of the IL-1 family in a variety of vertebrates, demonstrated a lack of Caspase-1 cleavage sites (i.e. the necessary aspartic acid residue) in IL-1β of chicken, Xenopus and rainbow trout (*Oncorhynchus mykiss*) suggesting that Caspase-1-targeted cleavage of IL-1β may be limited to mammals (Huising et al. 2004). These data, together with the absence of *Pycard* and *GsdmD*, would support the possibility that the NLRP3 inflammasome is inactive in the chicken. However, several recent studies show that NLRP3 pathway components are upregulated by different stimuli suggesting the NLRP3 inflammasome response is functional in chicken despite the apparent inefficiency compared to mammals (Chen et al. 2020; Han et al. 2025; Huang et al. 2021; Karaffová et al. 2020). All the mammals examined in our study have the cysteine-active site in Caspase-1 as well as the Caspase-1 cleavage site in IL-1β.

**Fig. 5:**
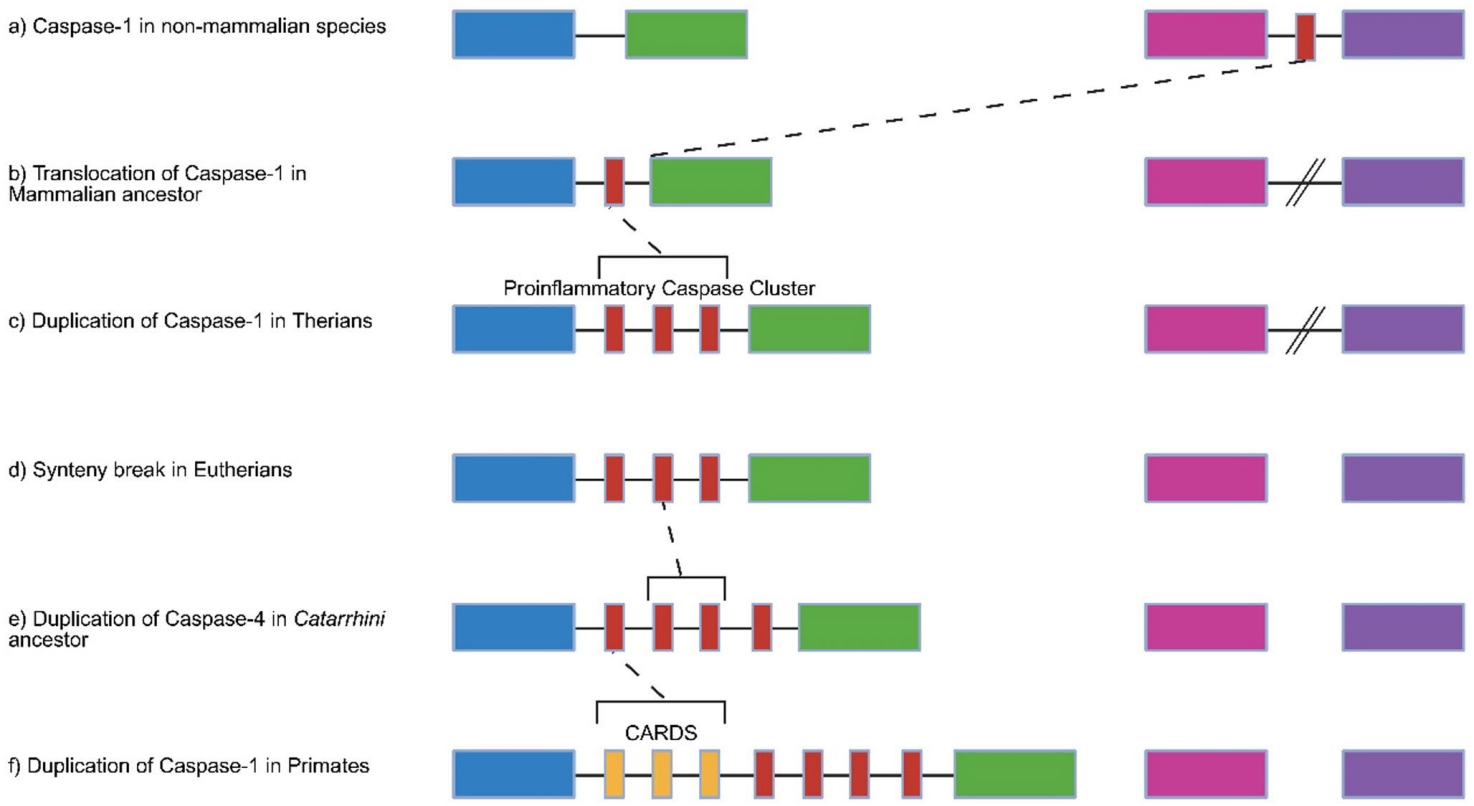
Evolution of the proinflammatory caspase chromosome region. In the mammalian ancestor, *Caspase-1* translocated to a different chromosomal region. The chicken *Caspase-1* syntenic genes underwent an intra-chromosomal rearrangement prior to monotreme divergence. In therians a duplication of *Caspase-1* resulted in *Caspase-11* and *Caspase-12* (Eckhart et al. 2008). The genes that flanked *Caspase-1* in chicken underwent an inter-chromosomal rearrangement after marsupial divergence with the upstream genes translocating to a different chromosome to the downstream genes. A duplication event in the *Catarrhini* ancestor generated *Caspase-4* and *Caspase-5* (Eckhart and Fischer 2024). *Card16*, *Card17* and *Card18* identified in humans and chimpanzees are likely the result of *Caspase-1* duplication. Homologues of *Card18* have been identified in various primates (Mao et al. 2024). Red boxes represent caspases, yellow represent the Card genes. Blue boxes represent the gene cluster upstream of caspases in mammals and green the downstream cluster. Pink boxes represent the gene cluster upstream of Caspase-1 in non-mammalian species (xenopus, anole and chicken) while purple represents the downstream gene cluster

Relocation followed by massive reorganization of *Caspase-1* in mammals might have facilitated changes in its regulation and function. Following mammalian divergence *Caspase-1* relocated to a different chromosomal region in mammals, causing the upstream and downstream syntenic blocks to relocate on the chromosome and, after marsupial divergence, move to different chromosomes (Fig. 5, Online Resource 21). After monotreme divergence the duplication of *Caspase-1* resulted in the generation of *Caspase-4/−11* and *Caspase-12* (Eckhart et al. 2008). Gene duplication events are known to be key facilitators of neofunctionalization and subfunctionalization (Conant and Wolfe 2008). The similarities between *Caspase-1* and *Caspase-4/−11* make this duplication a likely explanation for subfunctionalization. *Caspase-12* also shares sequence similarities to *Caspase-1* but does appear to be more divergent. Interestingly, Caspase-12 in mouse has been shown to act as a negative regulator of the inflammasome by suppressing Caspase-1 binding (Saleh et al. 2006). Human Caspase-12 has accumulated numerous mutations resulting in a nonfunctional pseudogene (Fischer et al. 2002). This is not restricted to human, as loss of function in Caspase-12 has occurred at least four time in primates (Holland et al. 2024). Duplications to Caspase-1 and Caspase-12 have been shown in opossum (Eckhart et al. 2008). Our data suggests these are species specific duplications as the Tasmanian devil contains a single *Caspase-1* and *Caspase-4* but three copies of *Caspase-12* while the koala has a single copy of *Caspase-1*, *Caspase-4* and *Caspase-12*. It is possible that these duplications were due to a functional requirement by marsupials to provide a more robust immune response due to their young developing externally in the pouch. Although if that is the case it is interesting that koala lacks these. Further expansion occurred in the *Catarrhini* ancestor where a duplication of *Caspase-4* led to the generation of *Caspase-5* (Eckhart et al. 2008; Eckhart and Fischer 2024).

Some species show species-specific variations in the number of proinflammatory caspase members that are present (Table 1) (Eckhart et al. 2008). An interesting example is in canines and other carnivores where a fusion event between *Caspase-1* and *Caspase-4* created a gene which encodes the active regions of Caspase-4 with the Caspase-1 prodomain (Eckhart et al. 2008; Eckhart et al. 2009). While the fusion protein is capable of processing GsdmD it cannot cleave IL-1β with this role being taken over by Caspase-8 albeit inefficiently (Digby et al. 2021).

Early studies in mice showed Caspase-11 to be essential for Caspase-1-induced apoptosis with evidence of Caspase-11 independently inducing apoptosis (Wang et al. 1998). More recent studies have demonstrated Caspase-11-independent apoptosis by Caspase-1 in a pathway requiring the apoptotic caspases, Caspase-9 and Caspase-3 (Tsuchiya et al. 2019). Caspase-3 and Caspase-7 are involved in Caspase-11 regulated apoptosis (Kang et al. 2002). Caspase-4 is also required for inflammasome activation in response to certain gram-negative bacteria using either a TLR4-dependent (Rathinam et al. 2012) or -independent pathway (Kayagaki et al. 2011, 2013; Sollberger et al. 2012). The absence of Caspase-4 in monotremes suggests this response may be absent or instead require Caspase-1. It is possible that the appearance of Caspase-4 after monotreme divergence may have allowed diversification of function between these two genes. In the case of the NLRP1 inflammasome, Caspase-5 interacts with the complex through the adaptor Cardinal and thus processing of both Caspase-1 and Caspase-5 occurs (Martinon et al. 2002). Both human Caspase-4 and Caspase-5 are capable of forming a noncanonical inflammasome that lack the sensor protein or the Pycard adaptor but instead forms from the direct interaction of LPS with the Caspase (Eckhart and Fischer 2024). This non-canonical pathway would be absent in the monotremes or perhaps performed by the monotreme Caspase-1. Interestingly the region containing the Caspase-1 family in humans is known to be a site which frequently undergoes rearrangements in various cancers, suggesting the large genetic variation may be due, at least in part, to chromosomal instability (Cerretti et al. 1992; Du et al. 2010).

## Conclusion

The fundamental machinery involved in NLRP3 inflammasome activation has been largely conserved in the mammalian species examined. The exceptions are the proinflammatory caspases, NLRPs and Dectin families which have undergone frequent lineage specific changes. We identified *Nlrp3* homologues in all mammalian species examined as well as the other proteins involved in the NLRP3 inflammasome. The conservation of Syk as well as the signalling molecules involved in the Syk-dependent pathway suggest responses to fungal threats are conserved in all three major mammalian lineages. However, the range of PAMPs that monotreme and marsupial NLRP3 inflammasome pathways can respond to may be different to those observed in mouse and human due to the absence of key Dectin family members (*Clec7a*, *Clec6a* and *Clec4d* in marsupials, *Clec4d* in platypus and echidna). The absence of many Dectin family receptors suggests that opossum, Tasmanian devil and koala may have fewer options to respond to PAMPs and DAMPS. Whether this leaves them more susceptible to certain types of threats is currently unknown.

This analysis shows that Pycard is conserved in all mammals examined however chicken, turkey and zebrafinch appear to lack Pycard, while green anole lizard contains a homologue. This indicates there may be differences in the components of the inflammasome complex in these avian species.

Caspase-1 is a key component of the NLRP3 complex and has undergone multiple duplication events during mammalian evolution resulting in an expansion of the inflammatory caspase subfamily as well as lineage specific duplications. Our data suggest that *Caspase-1* was translocated into a new genomic environment in early mammals, prior its expansion. We hypothesise that this relocation as well as chromosome instability in this region facilitated the amplification, functional and regulatory diversification of the *Caspase-1* subfamily. A comparison of Tasmanian devil, koala and opossum suggests the marsupials have undergone species-specific duplications. The absence of several inflammatory caspases may reduce the number of noncanonical pathways available to monotremes and may result in differences in the inflammatory response.

## Supplementary Information

Below is the link to the electronic supplementary material.

Supplementary Material 1 (Online Resources 1, 3-8)

Supplementary Material 2 (Online Resource 2)

Supplementary Material 3 (Online Resources 9-21)
