# Supplementary material for: Conservation of NLRP3 Inflammasome Pathway in Monotremes and Large-Scale Restructuring of the Caspase-1 Gene Cluster Region in Mammals
Source: J Mol Evol. 2026 Mar 12;94(2):353–68. doi: 10.1007/s00239-026-10307-6 (PMC13076529; doi:10.1007/s00239-026-10307-6)
Supplement: Supplementary file 2 — Supplementary Material 2 (Online Resource 2) [file 239_2026_10307_MOESM2_ESM.docx]

**Conservation of NLRP3 Inflammasome Pathway in Monotremes and Large-Scale Restructuring of the Caspase-1 Gene Cluster Region in Mammals**

David Stevens, Tasman Daish and Frank Grützner

School of Biological Sciences, Adelaide University, Adelaide, 5005 SA, Australia

Address correspondence and reprint requests to Prof. Frank Grützner, School of Biological Sciences, Adelaide University, Adelaide, 5005 SA, Australia, email: frank.grutzner@adelaide.edu.au

Journal of Molecular Evolution

Online Resource 2

a) Platypus protein sequences

>PYCARD_like_1

MVPVPRKLHFVDRHRKRLIDRVTGVDSMLDSLHGQVLNEEQYQNVRAEKTNPDKMRKLFS

FNPSWDQGCRDRLYQALRETHPHLVEELESYLVCKMGMKTVSST

>PYCARD

MGSARDHILTALENLTSDEFKKFKTKLLSCPLREGFGRIPRGPLMNMDVIDLTDKIVTTY

MENYGLELTTAILRDINQAEAAALQKAAGAAAPRVGPSKIALGEAEQQHFMDKHRQALIN

RVTSVDALLDALYGKVLSEQQYQEVRAEKPSANQMRKLFSFSVAWNRSCKDQLFQALKAT

HPFLIKGSDGSDQVPRSKRAKDHYTKRFEEYNIRVAFPGHNELPSGEGQPHFVDNYLICK

MGMEMVSSSWDHLIILYLPQRLEQCSAHNSKLAEGKECVCLLYRTLPSTSYGALPTVNAQ

QIRLTAFSPMEDRSNRKKEARENGQVVRQNQNGILFLKWLQSALAYNTTEHSCLSRFRCL

KWNGMDFVA

>TRIM72

MSAAPALMQGMYQELSCPLCLKLFESPVTAECGHSFCRSCLARLPQDPQAGGTPCPSCQA

PTRPEGLSTNQQLARLVESLAQVPQGHCEEHLDPLSVYCEQDRVLICGVCASLGKHRGHS

VVTASEAHQRMKKQLPQQRLQLQEACMRKEKSVALLDRQLAEVEETVRQFQKAVGEQLGV

MRAFLSALEKTLGQEASRVTGEAGTALQGERRGLASYLEQLRQMEKVLDEVTDQPQTEFL

RKYCLVTSRLQKILAESPPAARLDIQLPIISDDFKFQVWRKMFRALMPALEELTFDPATA

HPSLVVSPSGLRVECVEQKAPPPGDDPQQFDKVMGVVSHQLLSEGEHYWEVDVGDKPRWG

LGLISAEAGRRGKLHPIPSQGFWLLGFRDGKVYEAHVESKEPKVLKVEGRPTRIGIYLSF

QDGVLSFHDASDPDNLAPLFSFRERLPGPVYPFFDVCWHDKGKNAQPLVLVPASSVLSFF

SPLTPSAKVLLPHRPESSSGMKRGAEGWGRSLRGSEEEEHLGGARTGQRPNLCPECGRGF

SQRSDLVKHLRTHTGEKPYPCPACQRRFSRGSDLVKHQRAHRRAALRLCGLRPPLQPELS

LPGPSAAPPEREALFLQPQRPLLWPQVHPGQALADAARGRALSPTGPPVPCTTWSIYRVS

TVKEQTEGEAAEDGVDDQELAFNQQLVSFCCVNDNWYYLSTYLEFT

>FUS

XYSTQPTPQGYGSSGGYGSSQGSQSSYGQQSSYPGYGQQSASSSGSGSYGSSSQSSGYGP

PQSGGYGQQSGYGGQQQQQSSYGQQSSYNPPQGYGQQSQYNSSSGGGSGGGGSGGGNYGQ

DQSSMSGGGGGGGYGNQDQSGGGGGYGGGQQDRGGRGRGGGGGYNRSSGGYEPRGRGGGR

GGRGGMGGSDRGGFNKFGGPRDQGSRHDSEQDNSDNNTIFVQGLGENVTIESVADYFKQI

GIIKTNKKTGQPMINLYTDRETGKLKGEATVSFDDPPSAKAAIDWFDGKEFSGNHIKVSF

ATRRADFNRGGGNGRGGRGRGGPMGRGGYGGGGGGGGSRGGFPSGGGGGGGQQRAGDWKC

PNPTCENMNFSWRNECNQCKAPKPDGPGGGPGGSHMGGNFGEERRGGRGGYDRGGYRGRG

GDRGGFRGRGGGDRGGFGPGKMDSRGRVLGGEARDP

>NLRP-like_1

MEVSLTELHFVDQPSLREQLIQRVTSVDQVLDKLFGSVLSQEQYQLVRAESTNPNKMRTL

FSFSPSWTQNCKDLLLKALREIHGPLLKELERSPEMQSPQDGAAIRKRYRARMEEKFSLL

RERNARPGESVVLQHIFTQLLCLPEHPQREQKEHELLAVGWDHARTMEEKGQFVEVNALF

GPDKIRFRRPKTIVLQGAAGIGKTMLARKIMLDWAKGNFFEDIFQFVFYLNCREMNQLSE

RSLSDLISVHLGDPRASFDEIMSQPEKLLFIVDGFDELKWSFEEQEYDLCYDWSEKRPVP

ILMSSLLRKILLPEAYLLITSRLTSLEALNNLLQHPYHVEILGFSEADRKEYFCRYFGDE

NQAMQAFDLVKDNETLFTMCFVPLVCWIVCTCLKQQLKCGKDLTQTSRTTTDLYVNYLAA

LFPATQDRPSQDPPILRRLCHLAAEGVWTQKILFDGDDLRKHGLDMSDVSPFLQLSIFQK

DIDCENSYSFIHLSFQEFFAAMFYALGAEEEAMDDSITDIGDVKKLLEENEKSQWKGFLT

LTVRFLFGLLNEARVKDLEKKFNCKISQEIKKELLQWSKGHCEILDLETLFDCLYEIQEK

EFIRTVMNRFQRIHLEIDTRMKLLVSSFCIKHCQTLQSIRIGGGDLDCEPIIENDLDSRV

KYGMEWGETGVWEHCTEAWVGQRVGYSPVPQQQQQKWRFPMESPLKKAQLPSELAGKLPN

DAHENDYFTSRVKQAWKDLFSGLRTNQDLTELTFSKLFIESGMETLCEELRHPQWKIRTI

RLDSCDLTAADCQGLSSAFASNQSLKYLDLIFDDPENTGVKWLFEALRHTDCSLEALGLQ

RCHLTVASCKDLSSALASNQKLKRLYLTNCDLNARAVKSLCEGLRHPNCSLEILGLQSCG

LRDACYEDLSSILTGNKKLTRLDLFGNLLSESVKQRLQNDSKHANFNYERKSAQPQVGGP

KCPLNSGVPWALTLPGASFPSFSPMVGTMAVITLALPAISLSLLTSLVSFYILTSTLHSP

AAKLLLCLVLTCLAIDS

>NLRP-like_2

MAPAPRDKLLLSLKELREEELRTFKFKLLGIPLQDGYFHVPRGEVDGLKPVELADLLILY

YGEKYAVMVVLEVLKAMHMNGVVETLGQDTWEDSRETYRERMKKSLERAEERHGHLRDRV

DLRHRFTPLCLVTNSLPGAQEEEGPAPVAEGNGSSGIGEKEVHSIGLETLLDPEEEGTDS

PRTVVLHGEAGIGKTTLAKKLMLDWASGDFYPDTFDYTFLVSCREINLMAEKSLAELICH

CYGDGHAPVTEVLKRPERLLFIIDGLDELKYSLEEEGEEPGSELRDKQPVQTLLSSLVRK

KLLPESSLLITTRPRALEKLQPLLEDPRYVEILGFSEVEREEYFFKFFTDENKAKKAFNF

VQRNENLSSLCSIPLVCWFSCTCLKQQMVRGEPLTHTSKTITDVYMSYISIFLQPDRARA

KQPAYPTLKRLCSLAADGIRNRKILFQEDDLRQHSLDQADIAAFLSKANFRQESKGETFY

SFIHYSFQEFFSALYYMLEGDGNESNRDVNALLEKQEQTRSDGSALTLRFLFDFLSQETV

STLEIKFSYKIPPKLQKELKCIKARVKSLLSPIQKGPFELNISLNKIPKENNEQDSRDNF

QTMKQSKVSSKEGRMSVDMDKGFPQGIQGWIGDEK

>NLRP-like_3

MLFLRFDGLAHFELRLINQLIRIFCIPPAELGFVLFILRCLSAHYVPGAVLSTGVEDLLD

EKLNNFKFKLQDISLEEEFKRVPRGRLKPAEPVELADPLIRHYREDYAVKVSLEVLRAIN

QRQLAEELSQATGQVVVPIKVQSLKWSPREADDPDQGHNEGEDPAQRPQGLIQSSSEVMY

PPAVEYPSRIPQEDPHDVEDENSLLGANPEYLRLLLVKEHPGQLGSAGHKQMSATQVDAL

FDPEEEGRKPFRTVVLQGAAGIGKTTLATKILEDWAAGRLFHGRFDYVFYVSCREMNLVR

ERSVADLISHCCADKNGPVTDIVWLPKRLLFIVDGFDELRCSNERSRKDLCSDCKEKRST

DTLLASLMSKRLLPEASLLITTRPTTLEKMQALLEQPRWAEILGFAEAERKDYFYRYFKE

KKLARQAFGFLQGNDGLFTLCMVPIVCRIVCASLKRQMERGEALTQTSQTTTAGYLSYLF

GLLREDGTGPKAPRPLNLRRLCSLTAERIQNPKILLEEDDLRRHGLGSSGSTTFLKISIL

KKNHHPQKFYSFPHLTFQEFFAAVSYVQKTDFLLHVFYLLMRKNETRNNLATSSQFVKKL

VGQYGQFGSGYLTLTMRFLFGLLNEERPGEEPGLIEIGALATKLDHTVASFCVKNCPGVC

SVTLSSVYSAGGKGDDVNAVVEEGLSLGSQHESSHRLGKCFLPDAFCQNLSSALHTDPNL

LELNLSHIALGNAGLRVPSEGLMDQGCRLQKLT

>NLRP-like_4

RLAEYLEELRDPELRKFKFHLEDLAPAAGWAPIPWGRTEKADSLDLAHLLVAHVGERGAWE

LAVHVFERIHRKDLWERARAEAEVRDSSGSVLGRHWEQRRRAEHLVETHEAQATRDPRD

VYREHIRRKFRFFEDRNARPGECVNLSQRYTQLLLMERHPTPRDAAPVSPTPQREDAGA

PERQPGPLQLETLLEPDASWPEPPRTVVLRGAAGIGKSMLARKIMLDWADGLLYRARFD

YLFYVSCREMSRVGRGSLAGLISGRWPRREAPLADILRRPERLLFVIDGFDELGRTPRRP

PPGRRAGGWKAKMPVAGLLGGLLGKDLLPEASLLITTRPAALARLQPLLEQPRHAEILGF

SRAGRRDYFRKFFGDGRQASRALGLVRDVEALSAVCFVPLVCWIVCTCLKQEMERGEP

PGQPSKTTTAVYVFYLLSLLRPDPGGSGPRGRPVLAPLCSLAAHGVWARKVLFDEGDLS

RHGLAGSDVSAFLNLSVFQKDIHCQRLYSFVHRSFQEFFAALFYLTDEGGREGGRGSRH

SVTRLLEHYGRSETGYLALTVRFLFGLLNGENKSYLERQLGCPLSPTVKGELLAWIEAGAR

IGGRTLERGALDLFSCLYETQEEGFIRRALDPFQVIVACDLSTKMDHVISAFCVGNCRNASV

VHLGSEEFSSEEAAEGQGPVGTEGTHLGDQPLSPVEKCWLPDTYCEHLSSALRTNQNLAE

LVLDRNALGNRGVKLLCQGLGHANCKLQNLGLKKCRFSSAACQDISSALSANQNLVMMDL

SNNALEDVGVKLLCAGLRHPKCRLQSLQLKKCYFSWAACEDLSSVLSTNPHLMELDLTGN

ALGDAGVQLLCVGMKQTSCRLKTLWLKICHLTRASCMELASVLSVDCSLTELDLSLNDLED

AGVRLLCEGLGQPKCKLQKLRLGICRLTSAGCGAVSTALGANGHLKELDLSFNDLGDVGAQ

QLCDGLNHPNCKLQKLWLDSCCLTAVACESLSSVLAKNQTLTKLYLTNNALGDAGVGLLCE

RLKQPTCKLQTLWLFGMELKAETQNALAALRRTKPHLDIGS

>NLRP-like_5

MEFTERLRCAEHRTERLRESDPTESVDPSPALRELAVSPSRPSIRGGQGVGLLIVVLSSP

KRSVRRSAHTEHFVDRHRGQLIGRVTSVKPLLDLLHGKVLSEEQYQTVLAGATSFDQMRK

LFTYSLSWDRDCKNLLYQAMRKIHPQLVAELDGSLRFSGPRVPQLEKMGMKTRLGETDCT

VSVDTFPVHNELTVFETHSDVLVCLYPNCRQPWEGVSLAEDYREKYLKYVRWKFRYLEER

NARLGEKVALEVRYTPLLLVEEHRSLAQRQHELLALGRQPTSWVSRRVHVEALFDPDAEG

LEPPLTVVLQGSAGIGKTVLARKVMLDWAAGTLYPGRFDFAFYVHCRELNLERKRSAVQL

IQQCCDDDGVPLSEICRRSDRLLFLVDGFDELGWSSGGWPGAADDDDDLFVDWKDKRPVG

SLLAHLIRKRLFPKASLLITTRPAAAESLRPLLRWPRRAEILGFSEAERAEYFHHYFLDS

GRANRALAFIQENDVLFTMCFVPLICWIVCTGLRQQMDRREDLAQASKTTTAVYLSFLSS

LLRPARHLPSRVPPAHLRGLCSLAADGILSQRILFRKADLRKHGLPEDGLSAFLHVDVFE

KDVDCETLFSFVHLTFQEFFAALFYLLGPDETGPGPLPDARTLLQHYRLCDTGFLTLTVR

FFFGLLNRERVADLKATMDCVVRPEARRALVEWIDTSARKEALPGPTLQWLYCFYEIQEV

DFVERAMGSFRKIDIDVRTRMDQIVVAFCLRNSRNLCSIELMRFFNVAEADAAAAAAERT

PEVAADRRPQDWLQDTLCESLSEASANNRGLTHLALRNKNLGRRGAELLSKGLSHPNCKL

KSLRLVNCLLTPDSCRDFSSILSTDRNLSELDLSSNALKDSGLSLLCEGLRHPSCKLQTL

CLVKCLAHYQVHEEGVRSDYLVSSSALGENCRYTSQNDFTAEGGPFWKGLQSCALTSACG

PALSSLLTTSANLTKLDLFNNALGDTGVSLICEGLKHPNCKLQALLLRHCALTLNCCPDL

SSVLSINRDLSELDLSYNSLEDAGVCLLCEGLRHPNCKLQTLRLQKCKLTSRSCSDLSQA

LGINRELRELILHDNVLGDAGISVIWKGIQQPSCGLKILRLGKTHFSEKMEEEMRTVQKM

KPELKIRYRPARC

>NLRP6-like

MEVPDSEARPREGVIRDLLWEALEDLKQDEFKKFGHKLVTINHEGRKNIPRGRLENVDLG

DMVDALVQFYDGDVALDVTRKVLKKMDMKAAATKLKEKRRRGDDPPGPSDFPGPGLEGRA

GAPNLKSQVTVAGKGLAPGLGETEGFAEEAARPVPAASTGDHILAGAREGEERNARSVED

QQALTKSPDLARGAAADEERGRRPLGGGGGGRTRSGPRRSDANTFNELFHKDEDGRRPGT

VVLRGPAGIGKTMTRPEDHVRLGRGQAVPRPVRLRLLRRLQGGGRGGGGALAGRPGAGPL

PRPAGAREGHAGRAGPAPLRRRRRTSCPPCRGGGRAVLRPLREGGPAVLLRSLLRSRTVL

PGASLLVTTRPAAPERLRACLRSPRCAEIWGFSDRDKKKYFFRFFKDEGQARRAYRFVKE

NETLLAMCSVPFVCWIVCSVLRRQTESGHDPARASKTTTAIYLLFVTSLLDGCDAEARAR

GRADLRRLCRLARQGVWEDRGRFGPEDLRRHSLTGSVIPTLFLRVLTVQRDLRSEVVYQF

IDQSFQDFFTALSYLLVDAGEAEGEGPGGARPLPDGAGTAAAAAAAAGPDDAERVLGCAG

ARAVRPAVLAWLEREGREAGRQGALPSPAEHLPPAEHVPPAEHVPPRHVLPSEHVRLAEA

VGDEDPEEEEEEEEEEQEQLELLYRLYESQDWELTRRGLAARGSVRVVGVGLGRLDQAVL

AFCLRSAPPGLALQLHRCAFAGAADQARKGHRSWAGKLSRGLASGGSHRSDKLKSPKRSS

LWPLCEALGDPQCKLKTLTLSHCRLSDAECRDLAEALASSRTLTDLELLGNKLGALGMRN

LCLGLSQPHCPVETIRLQQTSSREAYLELVNVLWVSPRLQALDLGHSTLDGLTVARLCHG

LRHPNCRIASLSLRQCSLLPNSWAEVAAIFASTTTLREVDLSGNPLGAAEIGTLCEGLRR

PDCKVNRLDLSSVDLCEEAVKGLVALSKAKPELVVVNPALSAPPEANSDTFSMLYPICKT

GMKTVSPTSHNPITLYLPQCLERCSAQSKCLRSSIIIIVVTGGGHWTIGEGARLDLSGVS

GSSVA

>NLRP-like6

MALSIRDLLTQTLEDLLENEFRKFKWKLCEIPLWASPRGQGGAASIPRGVLEKADPLTTT

ELILSYCGSSSALDVAARVLENIQQRELSNRLRERVPGKISQEMYRSEIRHKYERVKDYN

SLPGAWRSLEQHYVAPLIIRRCRPASEREPELLSKGPRHLELLRLCGEGGSDRVHLDHLL

DGPGGRRPLTVVLQGAAGIGKSYTAHKIMLAWASQRLYHDRFDWVFLFNCRELGVEPRPR

SLVDLVLSDCPALGPHVGQIFSSPQRLLFLLDGFDELQLPRSLEDDERGAEEEGEEEEGQ

EEEAAASAGWRRAGAAVRRRRPAAATVRLLLRQRLLPGCLVVVTTRPSALEQVEGCIRAD

VHLEVLGFLEPERQAFFTRFFGDAKRGREAYEAVQGNEALSTMCFVPLVCWIVCTVLHKQ

LEKGQGLDGLQTTTQVFLHFLSILLRFHRRRGARAPADSLLEQLGTLAVHGLMARKVIFD

QEDLEAHGLPAAAPPTIFLSAVLRQGVTVETVYSFGHVMLQEVFAAIFCFLPGRGARPEL

GLAHLLEAGLQAENGHLLQTIRFLFGLSHPQCQAMLRQLLPHRAALTPSPEGALLSWVQR

SAESPRAEDPRFLLELLHCLYEWHCDGLVGRVASELNIRFLLFPLKRSDCLALAYCLGCC

ASVSCLHLYSCGLDQGDIRLLLPALGKCQALHLGLSDIPSGLMQEIGRSFSPKQSVTSLL

LQGLGSNHSNSQKETVFKVSALWGSEPCSLRVNNVDKETTLEFCFWAVPAHRPREVTLQG

TQLCESSFRQICRLLRFSYSKLESLRISGNLLTKGCIPHLELLVRANTGLTHLDLSGNSL

GDEAVVLLCAHLGAAAGQLRNLRYVDPGEVPRLFGPCFPSSIDQWDLLVLCAEHCTKHLG

SGPREASLPLNSLVENGLTQECVPALSSLLPKLPALTCLKLGFNSLGDPGLLILAPALSD

PTCGLLKLDLEANGLTDACMPTLAQALAQNQTLEALILNGNKLSNLSIPHLDVIWREATH

LCRLELLFNQLPSTTRSRYSMAQAEPRDDVITTTPLYRETRGHGTRPRLTQRSSRPVPMP

NDGHRAVRVF

>MIS12

MQKGAGKEKGLRDAEANGPPPEVLEEWDVLVEKGFGEQISSSRVKYRFFVPPPPVPPPAG

SPPGFRDRVRPSAPEERLATDERWTNTVIITVVITVIVAVASLPGPSSPSSPPPGGVPAA

EMSVNPMTYEAQFFGFTPQTCLLRIYVAFQDYLFEVMLTVERVILKRLEAAPGSGVSPVQ

IRKGTEKFLRFLKERFDGLFGTMETVLLQLVLRVPDHVLLPEDRSHARHPRGPEELARLR

EEADRLRGRYEAEVRAGRALLAELEEQRAARAELEKTLRWFDGLENAWREHGSGDPRESL

AFLIRSSDRLRAVVGDVERKGRRLHLS

>PGGHG

MGYGALDDPAVFTSPTLPSDPRFLATLTNSYLGTRVYRDILHVNGVYNGALGDAHRADVP

SPVNVRLEAPEGVEVSQSFTLDTRTGTFLHVVETPEFTATHRIYAHRALTHLLAFSVTVR

RSAPQAQPITVRLRSDFTPKSRDLDLHLGPDFQGARYLSGRTLSPEVAGGPQPTVHMLWT

PAPPALTLPEARREATWQFLVAVAEAEAEVRRLFEEGAALLRAGSLYPAHVEAWGALWGA

SGLDLDAPLPLQRAVRGCLYYLLSAVPSPAPGVRDPFHGISPGGLSNGSRGEDYWGHVFW

DQDLWMFPNILLLWPEAARAILQYRVRTLSGAQANARDQGYKGAKFPWESAATGHEVCPE

DIFGTREIHINGAVLLAFEQYYYSTRDLQLFKEEGGWDVVSAVAEFWCSRVIWSPEEQCY

HLRGAGARGKLGGCVGPRTSCLSSSFLTPASQINHPIRNDISRGVIPPDEYQTDVDNSVY

TNVVARNSLRFATSLGRDLGLAVPEEWLRVAENLKVPFDPKRRYHPEYDGYRLGDSVKQA

DVVLLGFPVPCAMDPDVRKNDLEIYEAATSPRGPAMTWSMFAVGWLELREPERAQQLLNK

CFANISEPFKIWTENSDGTETVNFLTGMGGFLQAVLFGYTGFRIIRDCLKFDPVCPTEVR

HGQVTGVSYLGNKLNFSFSEEEVTVEVTWAQSQAPALEAVLEPSGRRLALPQGQTVSFPT

TAGRIQRVSSYTT

>PSMD13

MKDVPGFLQQSQSAGPGQAAVWHRLEELYTKKQYKQGCSFKHPLMKTVSLTWDNLMTLFL

PQRLEQCSARSHTASREFSWFEFWQGMNVDFGGPDHSKRLVQCSAHSCGINSLFRSSILC

RTPALPKEMASSRRKQFSAASCPGSEGRRYRPQRLARSKRLTDAVTAPRTALRQLHQRVR

AQVRLPLRFLPRFLGPLALPYRRKSWWCCHYRVNPLSLVEIILHVVRQMTDPSVALTFLE

KTREKVKSSDEAVILCKTAIGALKLNIGDLPVTKVSRPTPGSHAFSPGTPEGREGGGAEV

PEVSAASLILPVLGREIGTVAPWPATTCVLPYPPPTCPRVETIEDVEEMLNGLPGVTSVH

SRFYDLSSKYYQTVGNHASYYKDALRFLGCVDVKELPVSEQQERAFTLGLAGLLAEGVYN

FGELLMHPVLESLRGTDRQWLIDTLFAFNSGNVEKFQALKAAWGQQPDLAANEALLLQKS

QLLCLMEMTFTRPANHRQLTFEEIAKSAKVTVNERSDPVAPGSGPRRTAPRSAGVQVELL

VMKALSVGLLKGSIDEVDRRVHMTWVQPRVLDLQQIKGMKERLESWCTDVKSMEMLVEHQ

AHDILT

>EIF3F

MAAAAAAGPAAPAAPAAPAAPPAPAAPAPEAAAVAVPGAAAAGSAGSAGPGPAPGVPSGP

ALGGPFPGGRVVRLHPVILASIVDSYERRNEGAARVIGTLLGTIDKHSVEVTNCFSVPHN

ESEDEVAVDMEFAKNMYELHKKVSPSELILGCTQDSAWHMRLEQCSAHGKRSTRTVVVIF

AVRLARPVNAADGSGALPGVGGGDRADPKRLRVPFRRYATGHDITEHSVLIHEYYSREAP

NPIHLTVDTSLQNSRMSIKAYISASMGVPGKTMGVMFTPLTVKYVYYDTERIGIDLIMKT

CFSPNRVIGLSSDLQQVGTASARIQDALSTVLQYAEDVLSGKVSADNTVGRFLMDLVNQV

PKITPEDFETMLNSNINDLLMVTYLANLTQSQIALNEKLLSL

>MYADM

MPITVTRTTITTTNMSSSGGNHTIVGSPRALTTPLGIVRLLQLLFTCIAFSLVAHIGGWF

GPMGDWCMFSWCFCFAMTLVILLVEMGGLQPRVPVSWRNFPITFACYAALFCLSASIIYP

VTFIKHHDKSEEKDCRIAATVFSILAFLAYTTEVCWTRARPGEVTGYMATVPGLLKVVET

FVACIIFVFISDTNSYERHGALKWCLAVYCIFFILSLAAILLCVGECTSWLPCSFHTFLS

GYTLLAVLAYATATVLWPLYQFSHRYGGQSRPNHCLREYGTLCFWDKLLVVAVLTAVNLL

AYLADLIHSARLIFVHV

>CASP1

MQPEEFHFHRIFLLLLPEKLKEDGCELTVSMPSEHKRKKAAKFLYRTLQNIPKTQLLKDR

WCLIIESLTHGMISGLLDDLLQMQVINQEEMDTVREEHHRPAEKTRALLNSVIPKGDLAS

QIFIDSLCKKNPFVAAKLGLSAVPQALQAPKTLTESHPDGSGEILKLCPSEEREKLQKEN

EGEIYPVLVKAGRQRQALIICNIKFEELCERVGAELDIKGMKKLLEDLDYTVQVERNLSA

TEMESKLKMFAGRPEHKFSDSTFLVFMSHGILEGICGTKYKKQEPDVLSYSTIFRVFNNI

NCPGLKDKPKIIIVQACRGENEGMAWVSDSLGPSATSSQEPEDLENDAIHRTHVEKDLIA

FCSSTPDHVSWRDPKTGSLFIVQLIKCFQNHAWNCDLESLFLKVQRHFETPKQKLQMPTR

ERATLTKRFFLFPDEGTEAQRSEVTCPQSHGRQVAESEFEPMTSDSQARALSTEPRCFRG

PSVCPLTVKHEGGERSNMAQRKEHRPRSRRDLDSNPNSATLASTVYGGSGEGVKGAQKEC

GMGQTRGNHSCMKSSGYAEGQDGRAISAVSWVSSFSDEVTEAQRSYTVTQVPSGRGGFEP

MTSDSQARALSTEPRCFTVSLFVRENSFHPPTIMNVFLN

>GRIA4

MEKAGQNGWQVSAICVENFNDASYRRLLEDLDRRQEKKFVIDCEIERLQNILEQCSAHSK

PSVNTIDRYTLPLNDGCKEKQANDKEYRSRTEFEFCVEDDDSGPGVPSTEHWGQYKQIGL

DTIPVPHGAHSLNPHFKDEVTEAQRSEVTCLRSYRSMVFIERLLYFEHCTTHLREYNREG

PSNQLRYESETTRRHLITTARAKSDFPTGWWPKLIFDPRHQRDRITEDTKADGGLRNDTG

YHSLRSEIANEKSDLLTMRSHTFGHKATLNLCLRKVAGKRGSATCQLCDCGQVTSLLSDL

ICKMGMKTVSLPWDNLMTLYLPQRLEQCSAPNKRLTNTNITITLLCQIVSVGKHVKGYHY

IVANLGFKDISLERFMHGGANVTGFQLVDFSTPMVSKLMQRWKKLDQREYPGSETPPKYT

SALTYDGVLVMAETFRNLRRQKIDISRRGNAGDCLANPAAPWGQGIDMERTLKQVRIQGL

TGNVQFDHYGRRVNYTMDVFELKNTGPRKEAAFKAHLREAVSICLTPVPGKLSDSTFHSL

YPESNSSSRGLSGLGDKILARTLLRRRLQIPLSERVQNPNAALAHSQHSRHDLGLTSITR

QARGTAPQPLYFTALPEAFDNIDKPEVGYWNDMDKLVLIQDVPTLGNDTAAVENRTVVVT

TIMESPYVMFKKNHEMFEGNDKYEGYCVDLASEIAKHIGIKYKIAIVPDGKYGARDAETK

IWNGMVGELVYGKAEIAIAPLTITLVREEVIDFSKPFMSLGISIMIKKPQKSKPGVFSFL

DPLAYEIWMCIVFAYIGVSVVLFLVSRFSPYEWHTEEPEDGKEGPSDQPPNEFGIFNSLW

FSLGAFMQQGCDISPRSLSGRIVGGVWWFFTLIIISSYTANLAAFLTVERMVSPIESAED

LAKQTEIAYGTLDSGSTKEFFRRSKIAVYEKMWTYMKSAEPSVFTRTTAEGVARVRKSKG

KFAFLLESTMNEYIEQRKPCDTMKVGGNLDSKGYGVATPKGSPLSSPVEIKVISSRGSDT

VVTPYEEVTI

>GRIA4-like2

MENGNANPYCTLVPGGLYFVWIRTVGDIKAWPSGFGKVQYIRVGRHVSCPPAAYSLEGET

DININEEILAAERLVKQGTKGNLEKETKLKKILHTQSRSKTGDLVVVTVSQLGRWLVSGR

PSATSQNSSALGSSGTGEIRGRRPRFTARKEAMVNHFRIFTKKPLWIRYQNDRRWRWGVL

GEMCPWCRYGSEMTRQHKTREAMQIDKECAQSASQNTQPFHLSTPIAQRNEARPGSSETE

RHVRFHSHPASTHASSSEEHVIRTIQPPGKPKVTVLASYWREAREDLISSECRILFRVTR

GFEGGERADLSHPEKDGPVRVEDMSEESNEGELGMRDICSQYSRGVFAIFGLYDKRSVHT

LTSFCSALHISLITPSFPTEGESQFVLQLRPSLRGALLSLLDHYEWNRFVFLYDTDRGTY

CWGSYKLIGLDTVHVPHGTRSLNSRCTDEGTEAQRSEATCPRSHSRQVAERGLKRRSS

>GRIA4-like3

MERRGPRKKGEEEARQARQAARDPRVATEPRIEGNMGHLSRSGGRRGFPEPGEPAATHSL

TFPNQFHRPAGGDAYLFERCSEKPPSPVPKIQKTTRQSVCK

>PDGFD

MSTNRGCDWGAGGSRVLSTVSVHLLEQAPSRATECLLGTFLGLPCGFIACANSLSLGKKG

NEEIFKLNPETSHRFKCNSDKVTEEQRSEVICQGHTAASLRGKKRSPVPSPRGNDAQYLS

PVEKSCSRNRRTRVQVPVCLLGMRHQWVNVSHPKNRPTRAFLEVDRANGNLGKELLRLCQ

LRPQWPLPVAVVFIESRLRSEPCFNTWETRALSTESRCLLRTYCAKGTALTRWERRVQQW

AFDKASKRGRVIVGYTEGGSETGRGREVGGGEKIESLNCNSRNPDRYPLGPSIGTDTNMK

VVIRQKFTDSIGTVGDLYRKEETIHVTGNGCVQSPRFPNSYPRNLLLTWRLYSQGNTRIQ

LAFDNQFGLEEPENDICSLGSGWRVEDNPLLVKTHPCWATAARESRAKTRVCCTGGGNDK

PLPHFYQENSTDPLSERSQMEGGAFWERCVRGVAMGRKRLDGIRQEVLWDQVPSSVKRGL

RLRAPRVTGTVPNSITLNLPQCFEKFMAHTLSTTLCPRCDTVNTISARTAKQNRQPLVGV

TSWTRSTSLSRPTLTMQPQWPGTGERSSIRSYLLSTYCVQSTVRSTWESTIQKKTDAFPS

HNKLTV

>PDGFD-like2

MTEIHLNPLKDTEFSIKIQTLKVSISKQVTEGDEGTEAQRSEVTCPQSHSRQVAELGFEL

MSPDSQARALSTEPRCCCSVTSDLKLIVGRENVCLLLYCTLPSTWYSALHIDNFQPAASE

TNWESVTSSISGIDYHPPSVTDPTLTADALDQTVAGFDTVEDLLKHFNPETWQEDLENLY

LETPHYRGRSYHDRKSKVDLDRLNDDVKRYSCTPRNYSVNLREELKLSNVVFFPRCLLVQ

RCGGNCGCGSPNWRSCTCNSGKTVKKFHENSFETLSLLRVYRRKGHTASLHEELLPKPWT

GRPPWPALPPVRPSLLLTEGLVPSRRWDSSYSNRSIRSDVYREGELEALKPSD

>MTMR4

MDCSPEPRRRRQIRRFLEDPEEAELAQFVQEFPGGDGGGGGGGCRRPEPEEPSSRDPEAL

PAAPEPDPRPPARPWPPDGHQHISAPAPLSPLTRPRSPWGKLDPYDSSEVGAGPFSPRQD

DKEYVGFATLPNQVHRKSVKKGFDFTLMVAESGLGKSTLINSLFLTDLYRDRRLLNAEER

ITQTVEITKHSVEIEEKGIKLRLTIVDTPGFGDAVNNTECWKPLADYIDQQFEQYFRDES

GLNRKNIQDNRVHCCLYFISPFGHGYDSPRPPPGQSEDRGRGPGLRPLDVEFLKALHQRV

NIVPILAKADTLTPPEVEHKKRKIREEIERFGIRIYQFPDCDSDEDEDFKLQDQALKDSI

PFAVIGSNTVVEARGRRVRGRLYPWGIVEVENPAHCDFVKLRTMLVRTHMQDLKDVTRET

HYENYRAQCIQSMTRMVVKERNRNKLTRESGTDFPIPTIPPGADAETEKLIREKDEERAA

LSAREGTDIGIGRRLPCPRGTHGLEGTAGVKIDSAYGPKSRGPEEGVDIGCLKDPIDRPV

VFIERSLCAANLGEYNATDLVDPFPAHEELTVQPRVRHSYTRRSGVAQWLEAGPGSRRVE

GSNADSATYQPCDPRRVCCPSLGLSALVGKMGMETVSPRVLCAMSALKKYGRVNEWMSVR

QDPGRGQGRRGAAWLGGSPEPHRMGSAMAEAPFCPGKSPGPGPPFPPLIGPGRVDFGQMG

QEGGQRDRQEKQVPGTQGPHPSRMLPGRQTAGAPAVAFGHPLRPGSGDAQVLPSGRFPLR

PPMSPGPAPSPKRKSRFPSFRKGEVGADGSCPVSRPPRAAIAPPPGPSFGLDLFPGPSRP

SPGCPRFPPPGRRRDPHSRPARRRRLTGDGPPLGKRVFIGVILEPVGAVLSPRAGILIAG

PGCSSRVGGQDTRQGARTLLGLRRDNHNDNSNDVSDAYYVPSAEKQRGSVGRGRAWESED

VSSDPRSATCLLGDLGPATQPKPREVPSSGKWGRSPEVTEPVEAKRVGEGHRVENPAGPP

ARPEGRGGRPRDPSATMNLSARGSGSCSVLSCFGEEGPPSLEYIQAKDLFPPKELVKEEE

SLQVPFAVLQGEGVEFLGRAADALIAISNYRLHVKFKDSVINVPLRMIDSVESRDMFQLH

ISCKDSKVVSALFLALPLPPAHPGSRPLRGRCHFSTFKQCQEWLSRLSRATARPAKPEDL

FAFAYHAWCLGLTEEDQHTHLCQPGEPVRCRQEAELARMGFDLHNVWRVSHINSNYKLCP

SYPQKLLVPVWITDKELENVASFRSWKRIPVVVYRHLRNGAAIARCSQPEISWWGWRNAD

DEYLVTSIAKACALDPGGKVAGGSACGGNGEGSEAGDTDFDSSLTACSGVESSSGPQKLL

ILDARSYTAAVANRAKGGGCECEVLPNCEVVFMGMANIHSIRNSFQYLRAVCSQMPDPSN

WLSALESTKWLQHLSVMLKAAVLVSNAVDGEGRPVLIVALAKILLDPYYRTLEGFQVLVE

SDWLDFGHKFGDRCGHQENAEDQNEQCPVFLQWLDSVHQLLKQFPCLFEFNEAFLVKLVQ

HTYSCLYGTFLANNPCEREMRNIYKRTCSVWALLRAGNKNFHNFLYVPGSELVLHPVCHV

RALHLWTAVYLPPSSPCTLREESVDLYLAPAAQSQEFSGRSLDSCSVCSGPLCALSGCSD

LAWDSGAS

>ABHD11

MLRRACAWRLRPSRGLSLARAWSNEGPRPVPLSYTQFDGPTQEAPLVFLHGLFGSKTNFQ

SIAKSLARQTGRKVLTVDARNHGESTHSSEMSYEAMSADLQALLSQLGLPRCVLIGHSMG

GKTAMTLALQKASGPGPERPTPCIRSGKSRRAQLERGGSVGERWILRSVKVPFGSPPPTR

SCPYHGGGRSLEGQGEPELVERLVSVDISPEETTGVSDFPSFVAAMQAVRIPKELTRSQA

RKLADEQLKPVIQEVSVRQFLVTNLVEAAGRYVWRVNLEALTHHMDALMGFPQLPGTYSG

PTLFLGGSNSQFIRPSHHPKIRRLFPQAQILSVPGAGHWVHADQPHDFTAAVRDFLT

>IL1B

MPAYGEQDSSREVEETDDKKTKRQPGLESTMARVPDQSRDLMECYSGDGEDQFYEVDGPS

QIKSGFQDLKARTCQETRIHKEDKCSPCQMGIELKVTELPSSHGFRKAVVLVVAVERIKR

QAVSYNTSFMDRDLMDIFTSIFKEEPISCSTWEQTLVTDSLYHYLRCQEVTIWDEEHKSF

TLNTMANPCELRALHLIGANATQEVKLNMNFYYKTERLAGPTVKQPVTLGIKGGNPGNLY

LSCVKKGGKPTLQLEVVNKSDLLGKNQERFIFNKSTEGTSTTFESAAYPDWYISTSREED

EPVFLGASKGEEAITNFFLH

>IL18

MNQGTGGELQGAFEDGFESDAFMKHGNPKFQVFQDSSTKVLVMKQDSCTPRFEAMSEQEIKDNAP

QTKFIIQFYKNTKPGCYPVTISVKSGDRTFHLSCQGNILHFEVRLMSVWFADPGETQIQRGPSGKEGD

KKQNATLSWQTH

>NEK7

MWDNLITLAAGNCCLNSCTGENVITSDEKLLLFLHLFWGLQLFPTFLLQKALRPDMGYNTLANFRIEKK

IGRGQFSEVYRATCLLDGVPVALKKVQVRSFVSYKSKLPAAFET

>LHX9

MTAALHSLSGFERGCGPWTALGRDGGNAEERGAALRGGRGGRRERAGTGQRKAEVGLSGEQELV

LGEQERGRSSQREQELVLGEQERGRSSLGEQERGRSSQREQELALGEQKRGRSSQREQKKGKEL

PEGAGAGPGGAGKRKERPGGAGTDPGGAGKRKERPGGAGKRKERPGGAGTDPGGTAKRKELPG

RAGKRKELSGRAGKRKELPTGTGPGPGRAGKRKKRPGGAGPGPGRAGKRKELAELEKEDWVANG

TWIGVPLQEGLEVGPAVDQVVLHAEVVAGGQGLGAGGAGEAAQVIDGLPGPHDHLGGGNPKVAAG

TSLHGEPSERKQGRRDHKGLTETLFLNSNKEGPHPHVLFWGVFFKILSELGLAGYFLSLPSFRPRTP

WFKAPLRPVSCRFFTTSFQTGKPNKGSPSA

>GSDMA

MLHAARHLLLLLLRLLALGTGGSPTSPTPTWTPARGCYRAEEDGEPTFRCSYAGLGAIPE

GIPNDTRKLFLDANQLGEVPAGAFEHLPVLSELDLSHNAIARLSGAAFRGLEGSLRLLDL

SANLLAAVPAEAFSGLRAATNLSANPWRCDCALQRLLRGMKLAEGTGAGIVCATADRPEL

VGRQVLGLEGEAGPCGARQGRRGTDAALLVTVGGWLALVGVSLARYVRRNHEEVRYLICK

MGIKTVNRMRDNLITLYLPQRLEQCLAYKAPPSPPLYCSSLEADFPSGFQAGLDLPEYSW

EEALRGKRRGLVLAGPWLGSEGSLRCSVVPKCHLLGVSPAGGRSNIPFIPDKKQKTFPSE

TIGSKSLEEEEEEERDFRVLQAEVEMEMYALKALTLGQREGLLTTLLGIMGQAKALQTLE

DTVEQALDMEEPVQLAQPGSAILPILKEDSGRLNPTLSGTVLYLLGALRELSEEQQQLLA

MSVEKEILPQQMRLLETILEQHFLREESGPGQLHSALLSGLRGEEWAVTQALLSLSGLDL

DENELLFTFDPEALPQLSALYAALSIFHLLAKTCSFSSSGLPTQPGILASLLPQGPDWGQ

GAVACSPSYLICTVRIKTVSPSGDVDCVQPDDLVSTPVLSAVSGRRARLRQTGPCCSPRR

RAPAEGEEEGDQGCSSVGLGEEGGPPNLNVSTHSPRTMTAMFENVTRALARQLNPQGDLT

PLDSLIDFKRFRPLCLVLRKRKGTLFWGARYLPTDYALLDLLEPGAAPTESTDNPHFRFK

KLLDLRLEGKVDVPNTVKVTGGAGLTQSSHLEVQTLSVAPKALDTLRDERADQGPTPSRK

LLPEHPFLQELRPRGENLYVVMETVETVKEVTLERAGQAQGGFSIPLLAPLGLQGSLNHQ

EAVTIPQDCVLAFRVRQLVTKGNGEWDIPHVCDEKLKTFPPEDKTEEEKFTCESVEGAEV

PEDFGALQEEVESEARQLAQLTPEARATLLRSLRALLGKNHQLRLLEGSLEGALHKGTPG

ALEDLGNVTLSPQAMGSILYFLGALTELSEAQQKLLAQSMEKKILLTQLKLVERAMEENF

QQSQGGDFPLPPQLLSSLGDEDLTLTEALVGLSGLELHRAGPRYTWEPATLPRLCALYAG

LSALHLLAAPAS

>GSDMD

MTFSPTVTENSQPGKTRSGVGRCRIHGLAWSSHRWRRSGMPRLVFMPRPAFFRRSHGGRY

RPRHGQGVLSHHPDPTADPSWLPLQAIRVTGRSLRRGRKICFLSWDRWSEEVSSVVLQSG

LHVAKPYFLIKMKGSPQPPGSMAPIFAQLTKNVAKKINSEGELLPLLSMNNSKRFRPLCL

VRKKRKGTLFFGARFRPTNLSLLDVLDSDLPAPELKREDKFGFQDRVDGRLKGKVDLRDS

LLSVQVSGEIKRVQNYSLEVQIVLISPEDLDKMQKERKLKKNEPEELKELRRLGENLFVV

TKVVETLEEANLSSERQAEGGCLLKLLSIHMKVQGPIRSGRGKTGRPSCHLPDLHLWSQS

INSWYLLSANYAQTTLALHNHQEVVNIGKGCTLAFGLGHLIFRDKWKILSMPSKEKTFLS

KGLEEDPFVKDLAMAKDAKGFEGLRQEVRQEKQYLIYLDRQLKETILQAVQDLLGQREEM

QKVEDALEDAMDGKATQKLEGPGNILLTILKEDSDHVVPELTGTVLYLLGALLVLSDTQQ

HLLKLALEKKLLPQQLKLVESILEQTFPMSQEGHFFLTPGPEDEERSFTTALLEQYGLEL

SGPNSQFLWKPDALASLSALYGALSLLDRRN

>NAPRT

MAEAERAASPLLTDLYQVTMAYGYWRAGRARERAHFDLFFRQCPFGGGFALAAGLRDCLL

FLRRFRLRDPDVDYLASVLPPDTDPAFFDYLRGLDASEVTVRALPEGSLAFPMVPLLQVS

GPLPVVQLLETTLLCLVNYASLVATNAARLRLIAGPEKRLLEMGLRRAQGPDGGLSASVY

SYLGGFDATSNVLAGQLRGIPVAGTLAHSFITSFSGQERLQSGALAPGDLSAQAETWLTR

VCELLGRPVKDAHPGERAAFVAYALAFPRAFQGLLDSYSVMGSGLPNFLAVALALADVGH

RAIGVRLDSGDLIGQAQEIRRIFRTCATRFQVPWLEFIPIAVSNNVDEALLAQLAQKGSE

VNLIGIGTNVVTCPLQPSLGCVYKLVAAGGRPRLKLSEEKEKRTLPGCKAAYRLGGPDGA

PLMDLLTLVEEPPPQAGQELRVWPLGSGEESRTLTPATVETLHRLYFQRGQASLEISPQE

CESLPTLTQARAFAQESLSRLSSAHKRREAPEPYQVALSEKLHALLESLSRSSRGLSLIC

KMGIKPVSLTWDNPMTLYLPRRLERCCAQNPEEFLTLLMRHVLGLEPLLRLQCGGREELS

YWSGQRSWWCPASSSCLSLPSWAGLRLAEGGVGLAVTQFRGNSLGLWVATARAQLTADRS

GTHKQIGPRGTWKSSEEAALWIRGIIIVIVVAALVAASPVCSERLDSSLPCGLCDRRHRA

ALLRASPGPLGPAGGPLG

>MROH6

MAAGAGEGVRPELGEAEGPGNPEPPPQPKAKPTRARGGRPKV

TRRPRPAVAPGSQPPPCPVGALTLAALAEEIQSHRGDRAGPGQRPGDRGDGRTAEPASRP

PAPEGNGGRRGDRATASGEAGEDAASSPGNDQRRPQKRPPKQPRPQLPQGEARPECRPSS

PAQSTFHPLASASPCAPGPEQFPLASCFLTDLAVHTVACLTDAGFSGTQATAVCLSSTLE

AHGTILRDKVEELVHGLHLQIHRFSEGRARRAALRVLCSLAVEHAPDVVHGLLSHSLPCD

SAVELWRGLSRNQRVNVTVLVQLLWKLKGQPRVLGGSPAGPDGALQEPLAATRALGEMLA

VAGCVGAMRGFYPQMLIALVTQLHQLARCPPDNLSKARGPPQSKAAHPRSHAHCAVEALK

ALLRADGGRMVVTCMEQAGGWERLSGPDTHLEGVLLLASAMVAHADHHLRGLFADLLPLL

RSPDATRRLTAMAFFTGLLQSRPTVRLLRAGSILERLGAWQGDPEPSVRWLGLLGLGHMA

LHAGKVQHVEVLLPALLGALGEADGRLVGAALGALRRILLQPRGHSCTDSICPDVGARLW

PLLDDARDPVRSSAIGLFGTLVGRSPLLQRCATRDLVLDSLVPLLLHLQDQSPDAAEQSA

EWTLARCDRFLHWGLLEEIVTMAHYDSPEAFSRTCRRLVRWYPGRVPGFLDQAQGYLRSP

QVSIRRAAGMFIGFLVHHTDAGAVKEGLVDSLLHTTLLLYATGGQLLNEFSPEHLVQCSA

LNNYEGRISGDLRELECDPEASVRSATHVTLHQLRLASQDWASRPGRFSPRRLLRPRGRP

ARPWPLYEEGPFKRRSRAGLWGSHMGA

>ZC3H3

MEMEEKEQLRQQIRLLQGLIDDYKNVHGNSRAQPAAAAGPRWPLPAYRGRGTFGVGYPRP

VRGDFFPHRGLSWRKKYSLVNRPPGAAEQPEGRAPPSQDRPGPSPPDPPRRVRLGPDQNV

VVGIEAPSDPGSAGGSRTHRDVPRSDPGLQKKEGGAGASNGEEDADLVCRKERGDRRVGN

SAGSGPGGPGEPRRTVSENARGLTGQAPPARPRSSEGAAGGKAGPPVPDALRLQRLRPGR

EPPLRNSLAQAPLDVSGPGRRAPATRTAREPSLPGPCRTPKFKKTNYTWVASTVKAPRGP

PRRSLSPRAAAEAARRAPSTGAADGPIKPQPKADPAVRPRKPAAPSKPGGPSSKYRWKAA

GPTPATAAAFQWRAEAPGRSDAPPASPDRADLPAPSQASGGPSGWKPAFGETALSAYKVK

SRTKIIKRRGSVSLPGDKKSSLLPPATPKSHYSLRRKHGARAKSSPVLKKNPNRGLVQVT

KHRLRRLPAARAHTPGKEDCSRGSTSSDGFSREVGRQASAQNGARHIGRFTPVRAALPAP

VAAEPEDWVCLPVLKSGAFPRAGGQKAKPWGESHAIRSPRAGDAPLYFEVPLSRAPSGSE

LPISPLGGTDGRPDDFRRGSPRRPTGGGRRSDGRDPPGPAPHPSRRALAPGADTGRRASA

G

>Syk-like1

MSRRAACGPVRGPWRASPWASLCASRVGQAVSRPWRAGPCADRGVRARVRTVACEPVCEP

RGAGPCAGHAVRAACGPVCGPWRAGPCADRGVRACVRTVACEPVCEPCGAGRAPAMSCGP

RAGPCADRGVRARVRTVACAPVCGPWRASPCVSHAAQAARSPCRVGRVRARVRTLAFGPL

CEPRGAGRVPAMSCGPRAGPCADRGVRARVRTVACGPACGPWRASPRASHAAQAVCRPCR

AGRVRARVRTVACEPRGAGRAGRGVRAPVRARAPAMWGGPCANPCANASHVARAVCEPAC

MTGWRGPGPSAGRALRGRIHFPKPGSRGSPQCPSPPGPGVHFCFFRRGPATNRLHAGRNA

LPGPGPVPGGVCRVHGAVPDAHRVPRAEPDVSRVQSAELDASFGGQSAELGVYRVQSAGL

DAYRVHRAELDVYRVQRAVLDVSRAQSAELDASFGGQGAELDACRVQSAELDAYRVQSAE

LDAYRVQSAVLGVYGVRSPVLSAGERTAERVLFLERLRGAERCAERWGEDAIHSFVRPFV

HSFIDPFDGGASRPSGEEEEEDGEEEEGEEKEGEEEGDEEEEEEEEEGEEEGEEEEEKRK

RKRGGGGGRREEEEGEEEEEEGDEEEDEGDEEEAKRKEEEAKRRKGKRKGRRRRKRRKGR

GRGGGKEEGEEEEKRRMERKKRRKETRKRRETEEEEEEEKRRKRMKEMRRRRRGGRRRRR

RRGGGRGGGRREEEEEEGKRRRRKRRGEGREEEEEEEGEKKRRKRRGGGKEEEEEEEGKR

RRRKRRGEGREEEEEEEEERRRKRRGRGGKEEEGGGGRREEERRRKRRRRGGRGGCCVGF

EAAGGPGRQSRLEAREPPGGRPDDPVGARRIPSPRLFQVRAGGGARMASGGGEAAAQLPF

FFGNITREEAEARLEEAGLGEGLFLLRQSRSSLGGFSLSVSSGGRVHHYTIERDVAGAFA

IAGGRSHPGPAELCAFHGREADGLVCRLRDPCLRPPGLRPRAGPFEGLRETLIRDYVRDT

WNLQGQALEQAILSQRPQLEKLIATTAHEKMGWFHGAVSRTQAEDALLAAPKADGKFLVG

DDDDDDDPRQLERSLAHSKRLTDATDYHAPDWSIPSAQSSALRIIPSMMMMMMMMTPPPV

LSTEPDTRVRSRGPEGSFALCLVHGGRALHYRIDRDKAGKLSIPDGKKFDTLWQLVDHYS

YKADGLLRPLATACPRAGQAHDGQRPTRFRRGGGDGRGGGTLPGGTPPPPRTGVTTPNHP

AHLRPALPPPPPPHRASSAALCTQ

>Syk_like2

MRARACVCSRVSARVCGFQAGGLIGRIHSFQRAKKGYLLSADCVRSTVPNAWKAQFGNRE

RPSQPNNGLRAERCAGRLLLDASYYMQRLGWTPPTGRLLLYAERWAGRLLLDASYCMQSA

GLDASYRTPPPVCRALGWMPPTGRLLYAERCAGRPLYAERCAGRRLLYAERCASQGACSG

PFLPPSPFVRMLGSLLILSRPAKRCDEHLRAENAVVGVSRVQSAVLDSHYVQTAVLSVYY

VLGVGSVQSYVPGAYRVQSAMHIVSTDRALSAGSQSPSNPLMRLYKETRDALAHAKLGPH

AAGHGHQAAVNRLESSAELNPYVMQRGRRGAGQPLPEELREALPMDTAVYESPYADPEEI

RPEAVELDRSLLTLEEGELGAGNFGTVKKGFYRMKKAPPQTLRDVPDAPKESPQNPPDPR

KPPGPPKRPPGPPQGPSESVQSPPRTPKNPSGPPQNPPDPPQGPPPRAPPQKVQDGPGRH

TRARTHTAPAPVLSVSGSVAAPERARVCGGRWRPRRGDKAVAVKILKDGGGGGGGGVDEA

VKEELLREADVMRRLDNPYIVRMIGLCRAEAWMLVMELADLGPLNKYLQKNRHVQARNLT

ELVHQVCMGMRYLEENSFVHRDLAARNVLLVTQHYAKISDFGLSKALNADQNYYRAQTHG

KWPVKWYAPECINYYKFSSKSDVWSFGVLMWEAFSYGQKPYKSSGRPIRQQRERVQHNNP

APNELTFHARVPGFPVYHVVCVRVSPCRVTSPVCAPCVLCLCVRETHVCASETLRPVPQG

MKGSEVSAMLEKGERMQSPEGCPVEVYDLMKICWTYKVEERPDFAAVELRLRNYYYDISN

>Syk-like3

XELREALPMDTAVYESPYADPEEIRPEAVELDRSLLTLEEGELGAGNFGTVKKGFYRMKK

PSYGLPQTPRTPQKHSRSPVQSSRPPTDPHKPSGTPSDPPTGPREPHRRPPGPPGPFMDP

PQALSDHPVPQGPPHKPSGTFQTPPRSPPKTLQTLANRQDPPKDLQVPPKDPPSPSSPPP

GPLKTLQDPLKTLQTPPKDPPPRAPPQKVQDGPGRHTRARTHTAPAPVLSVSGSVAAPER

ARVCGGRWRPRRGDKAVAVKILKDGGGGGGGGVDEAVKEELLREADVMRRLDNPYIVRMI

GLCRAEAWMLVMELADLGPLNKYLQKNRHVQARNLTELVHQVCMGMRYLEENSFVHRDLA

ARNVLLVTQHYAKISDFGLSKALNADQNYYRAQTHGKWPVKWYAPSASTTTSLQQSDVWS

FGVLMWEAFSYGQKALQGAARSPAGDRSSGRPIRQQRERVQHNNPAPNELTFHARVPGFP

VYHVVCVRVSPCRVTSPVCAPCVLCLCVRETHVCASETLRPVPQGMKGSEVSAMLEKGER

MQSPEGCPVEVYDLMKICWTYKVEERPDFAAVELRLRNYYYDISN

>AUH

MAAAAAAAGVGLLGVGRGARGRFRFRLRAASASGAVRGLGSEPREEDELLRLRFLPDQDK

DEGTEARRSEVTRPQSHSRQVAELGFELMSPDSKARALSPEPVRMHVCLCVWTRVPASVD

ACAHVCMSVGAGAHVCMSVGPCAHVCMSVSARAHVCMSVGARAHVCLSVGAGAHVCLSVG

PCAHVYMSVGACAHVCMSVGAGAHGGMSVGACVRVGTRVHAWGQVCPLSSPGIVVLGLNR

PQAKNALSWNLIKQLSQSLEALKSDKKVRTVIVRSEVPGVFCAEECSAPSRRLTDTDLMI

RRGRRVCEEEEEEEEQEEEEEEKEEEQKGEEEEEEEQEKEEKEEEEEKEEEEKSRPEGAS

QDGGRTAQLPVPTIAALDGLALGGGLELALACDIRVAGGSILWKAPAPPNGLFRPPEVPA

QRAPPTGSPGSARCSIGCPVELQARTLGARLIILAWPPLPDGALSARLVALRRVGGLQQR

VLGVRLVALQSVQLQVRALPAPPGLGRMQLQDGALGARLVALRRVQLQERPLGPRFVALG

RKRPPRPPGLWIRTGRFRIGRSDCRSSPWGGWGCLSERSGLDSSACASSAKMGLVETKLA

IIPGAGGTQRLPRTVGPALAKELIFSGRLLDGDEARAAGLVTHSPPQNPRGDAAFLRALR

LARDFLPQGPVAVRAAKLAIDQGMEVDLATGLAIEEACYAQTVPTKDRLEGLQAFREKRT

PRYKGE

>NFIL3

XMTTVASNEVAEARRSAAAPPPDGPPPAPAGLRRPRPGPRAAMEALKPVPPRDDPEEDPA

AAVASAAPGPPPARLPPPLPPPPPPPPSSSPSSSSRRRRREFTPEEKKDAQYWEKRRRNN

EAAKRSREKRRLNDLVLESRLVALGRENAALRAELLALKARFGLLPPPPVKLEPPEAGSP

RGSEADEAGGGKTPSDGEDEQRVPKGPGPAAPAALPHKLRLKVRGAAPLKREGSEADLPP

PFALQVTRLQAWGLWPPPAPDGGGLSDRGAALRGLGGGRAVAASDSGPAGKRPVRSPDHL

SVKPLDKSPVGQAARQKTCQVIDPSVKPPNGLSIRPPKQQPKNLPGRSPNHRSIEPPDSP

AVRTPDKSGTRRPNPPTSRSINNPSRPRIACQSNHPTDNRPVVRTPRTTRRRTCQIAESA

VSQTTRWPINQTPPPPPPPPRPAGKRPVSQITQSAVIQTTGPARSMASRQPGKEAVRTPD

E

>DIRAS2

MPEQSNDYRVVVFGAAGVGKSSLVLRFVRGTFRETYIPTIEDTYRQVISCDKNICTLQIT

DTTGSHQFPAMQRLSISKGHAFILVYSVTSKQSLEELQPIYEQICQIKGDVHKIPIMLVG

NKSDESQRELDAGEGEALAARWNCSFMETSAKMNYNVQELFQELLNLEKRRAVCLQVDGK

KAKQQKKKDKLKGKCSVM

>ADAMTS10

MIKQSVLHHPMQALCTILLISLILQLMDHEPSDPLAHCRSSSPMERGNQTSISEFLLLGM

SNQVEQRQLLFLLFLWMYLLGVLGSLLIIILVIVSDPHLHTPMYFFLTNLSLADVCFLST

TVPKMLVNIQTPSKSITYAACLVQMYFFILFISLDHFLLTGMAYDRYVAICHPLHYTTIM

SPRLCGLVLAGSWLISSLHAPTHTLLVVRLSFCSNREVLHFFCELYHILKLSCSNILINE

VAVFVAAVVIGLAPLTGVLFSYTCIIFTILRIPSKGGSQVLDHAKYLASGQHPSQRQHSS

CCRFPPPPPHKLLTAMKGALGDGVIQEGQLGGNEDSGPIRVKLQGTGEPQETTTASRKLL

SQLKNKQPQSRSGHQCPMAVCFALAEEFLSSLKSYEITFPVRVDHNGAFLDFAPPQRQRR

SLGTRPPEPAEPRVFYKVEAPHTRFLLNLTLTSHLLADHFSVEYWKRDGLDWRHHIRREC

LYAGHLQGQRLSSKVAISNCHGLHGLIVADEEEYFIEPLSGRGSGVPEGEGSPHVVYKRS

SLQRPHLDAACGVLDEKPWKGRPWWLRPLKTAPTKPLGNQTQRGQLALKRSVSQERYVET

LVVADRMMVAYHGRRDVEQYVLAIMNIVAKLFQDSSLGNIVNILVTRLILLTEDQPTLEI

NHHAGKSLDSFCKWQKSIVNRNGHGNAIPENGIANHDTAVLITRYDICIYKNKPCGTLGG

MCERERSCSINEDIGLATAFTIAHEIGHTFGMNHDGVGNGCGARGHETAKLMAAHITMKT

NPFVWSSCSRDYITSFLDSGLGLCLNNAPPKQDFVYPTMAPGQAYDADEQCRFQYGVKSR

QCKYGEVCSELWCLSKSNRCITNSIPAAEGTICQSSTVDKGWCYKRVCVPFGSRPEGVDG

AWGLWAPWAECSRTCGGGVSSSTRHCDSPRPTIGGKYCLGERKRYRSCNTDDCPPGSQDF

RELQCSEFDSVPFRGKYYTWRTYRGGGVKSCSLNCLAEGFNFYTERAAAVVDGTPCRPDT

IDICVNGECKHVGCDRILGSDLREDKCRVCGGDGSSCETIEGVFAPTLTEGGLEHVQGQL

SDRPKPPPCVLQATTPRAGAFSELRLTTFLVEEDPPRVTALPENRPHHNRPVGLRSVSLP

PPALLNFQARCPATPAPSPGYEEVIWIPKGSVHISIRNLNLSLSHLALKGENDAFLLEGK

PGPSPQLRLPLAGTIFHLRQGADQPECLEALGPTNATLIVMVLVRSDLQGIRYRFNAPIT

HEALPPTYTWHYAPWTKCSALCAGGSQVQAAECRKQPDSSPVPSHHCKAHAKLPERQRSC

NTEPCPPSWAVGNWSGCSRSCNRGARTRSVVCQRRISPNEEKTLDDSACAQPRPHVLEPC

SSQSCPPEWAALDWSECNPSCGPGLRHRVILCKSGDHSATLPASQCSAATKPPTSMRCNL

RRCPPPRWVAGEWGECSAQCGFGQQLRSVLCTTHTGQLSGDCTAALQPPATQQCETKCES

SPTESPEECRDVNKVAYCPLVLKFKFCSRSYFRQMCCKT

>MYO1F

HAVRPGPTWAPLGYQWNLYCE

ELRPRGPHRTPPTTALLISQALLRCLLIIVIIVIVIGATPDCIPLSARDPTGQRAPKSVP

TMSFSLIQGTGQSPLYPSGPRTKQNGQNQAVPFGALRNSPWRARLSPRPPTATPHTSNSQ

WVLGNPVDVAGDCKLAVGRECACYIVTLYSALHTGSKERFHWQSHNVKQSGVDDMVLLPR

VSEEAIVENLKKRFLDDYIFASSQEGWGRGGGTKSGGEGRGWSEFPQSDSHWLNVKATQE

KVKQRDSPEIVALDPAVQTYIGSVLISVNPFKQMPYFTDREIELYQGAAQYENPPHIYAL

TDNMYRNMLIDGENQCVIISGESGAGKTVAAKYIMGYISKVSGGGDKVQHVKDIILQSNP

LLEAFGNAKTVRNNNSSRFGKYFEIQFSRGGEPDGGKISNFLLEKSRVVTQNESERGFHI

YYQLIEGASQDQRQNLGIMTPDYYYYLNQSETYKVDGTDDRSDFHETLNAMQVIGIPTEV

QQLVLQIVAGILHLGNISFREEGNYAQVESADCESALQAIQWRGAGARGRGPGKKGPERF

LGIWTLTPVLAFPAYLLGVDSGRLNEKLTSRKMDSKWGGRSESIDVTLNVEQAAYSRDAL

AKGLYARLFDFLVEAINRAMQKPHQEYSIGVLDIYGFEIFQRNGFEQFCINFVNEKLQQI

FIELTLKAEQEEYVQEGIKWSPIEYFNNKVVCDLIENKLNPPGIMSVLDDVCATMHATGG

GADQTLLQKLQAAVGCHEHFNSWSSGFVIHHYAGKVSYDINGFCERNRDVLFSDIIELMQ

SSEQDSIRPNEPGSTRTLNAVLDTYAFIRMLFPEKLDADKKGRPTTAGSKIKRQANELVS

TLMKCTPHYIRCIKPNETKRPRDWEESRVKHQVEYLGLKENIRVRRAGFAYRRPFQKFLQ

RYAILTPETWPHWRGDERQGVQHLLHSVHMEPDQYQMGRTKVFVKNPESTPHRIQSLCPT

AVFFIPIARSPPLMLHARFYLSGHILGFGSQVPTGQIPGTPALNLLPTFLPQLFLLEEMR

ERKFDGFARTIQKAWRRHVAVRKYEQMREEASNILLNKKERRKNSLNRNFVGDYLGLEER

PELRRFLGKRERVDFADSVTKYDRRFKSIKRDLILTPKHLYVIGREKVKKGPDKGQVQEV

LKKHLDIQVLRSVSLSTRQDDFFILHEEAADILLESMFKTELLSLLCKRFEEVTQRTLPL

TFNDTLQFRVKKEGWGGGGSRNVNFSRGSGEMATLKVSGKTLMVSIGDGLPKSSKPTKKG

TPQSQSRGRRPAPARSAPGPPRGTCRNGAPPPMAPSSSQQQLEQMYAGHQKQSRGPPAAM

LPKQGASRRTRARPPSEQNLEFLNVPDQGMAGMQRKRSIGPRPPPGVGRPKPQPRAPGPR

CRALYQYVGQDVDELSFNVNEVIDILMEDPSGWWKGRLHGREGLFPGNYVEKI

>TMEM131

MQGTFPPPASGSERNRSTPAADVARRLAAGDDVGRDLAGEEASILVEIISRLLRGSEEAC

NVIRPSLRKAVKSCSNNNNDASKAKRPSQFSGKITVKAKEKSYSKLEIPYQADVLDGSFV

RKHSGSFFCAPFANRGVIKLTSGPRILSTFWSWSVVWYSPKRLVQCSALDTHSNTVDGKR

GEAEEKSRAGGEGKLRSGEDDASRHGKSRFASVGNFQCLERRCPAAFDPVKLFLSGLTGV

IFRRDLGPVVSSGQDSSSDPIERPVYLTNTFSFAILIHDVLLPEEARIMFQVQNFSKPVL

IPPNESRSVFTLFFIPSSSSVHIDSNILLITNASKFHLPVRAYTGFLEPQIKTVPTQRRA

PGLNPHFADEVMTMVFVKRLLCAEHRSKRWGRYGVISSSHYFVVHPQAEEPFIDFGILSA

SEASHILFAVFNSNPIENTRVNIRNRFIYTNAHLLLYSLAIKGWHVIGDGLTVELLAAER

GNRTAIDARIPELVDASASAQSSVILASGYFAVFGVKLIAKDLEGIHDGAIQITTDYEGL

KPLKRAEDPSGPRTRIYFRSVCDLSRKSVFAGFPFMALKQPGDGI

>CLEC_like1

MAKTDLLIFPPKPGPLPDFPITADVTSSVKQRLRLSAPYGIGTMSNMISLYLPRVLIKQC

LANKMASEVTYADLKFQDSFKAQRIQEFDNIQEIEHPALSPVWRCSALGLLILCLLLLIG

LASLGILCDAAVTRPLLRTRVFQAKKTNTEQLNGLEKNLSLQLETIANISKEKEMIQSNL

TDALQEMATKLCRELSSSKPKSQICSSNVSPTTPHRHQSEKSHLRSVYGIHPADPATPPS

AIREWERVGFGMGAGPTKKPRATDFHSVACGFHRGLIAAAWGVASTRPLLRTRVFQKKKT

NTEQLNGREKHLSLQPETTANISEEKEMIQSNLTDALQEMATKLCRELTKNKQEYNDAEF

SDPLRSRWGTEQSGGHAGRGWRLKQYSASGKCSIITIGSLTDNVYEPICKLRYWSLPDAL

NNYNRLIDCWEEGMMPSDLRQKNVLPNKMREEKHPAPSPARRCSVLGLLTLCLLLLIGLA

SLGILCKYHWMGLEARKTNTEQLNGLEKNLSLQLETIANISKEKEMIQSNLTDALQEMAT

KLCRELSSSKPDSQICSSNVSPTTQHRHWSEKSQLRSVCGIHPTDPATSPSAIRERERSF

ISLQYNQYIWVGLSRNSSSSQWKWEDGSALSPDLISFSSKDTTVGKMCATTCDNVFHSSL

CTNNRYYICEKPAGLVKKFTAD

>CLEC_like2

MCQNGFQGWCLTLILKLFLNSFGILLAFHIIIFKKKSSTRPLLDTAVTRPLLQTRVFQAQ

KNIEQLNGQEENLSLQLETMANISKEKEMIQSNLTDALQEMATKLCRELSSSKPDSQISS

VTSICERGMKIPWITSSVRLLRSYARAAERCWRKSKHQADLTHFKFILSCLNSALSSARQ

NFSSLIDTHARHPRRLFRTFNSLLRAPVPPPPPSLTPNDLATYFLMKINTISYTHSLGEL

IRSHGFDYHLYADDTQIYISAPVLSPSLQARISSCLRDVSTWMLARHLKLNMSKTELLIF

PPKPGPLPDFSITVDGTTILPVPQAHNLGSSNVSPTTPHRHQSEKSQPWSVYGIHPDDPA

TPPSAIREQKREGRSWDGTRNFISYFQCNYNIWVGLSRNSSSSQWKWEDGSALSPGLLKF

PSYALNERKTCAYISAYHLSIDSCTNSHYYICEKAAGVVKKLTSV

>CLEC_like3

MGGDDDGGDDDDGGDDDDSGKISQTHPDLSRELSRAATMQDEDRYNVLNIPRRPFSQGPA

PTDKASSSSSAHSHIWRLLSLLVLILCLLKLIGLALFGIRFFQAQSQENGEATSPNDKPG

NPHADRSWSCSKQLEHLSSQNSNLSAALREMSTKLCQGITRNQPDVLDDFVEYNYEPYPE

RWICARDNCYYLSMVTKKWGESRKSCEALNSTLVKIDNKEELSRLVEYWWKLSRGDPERA

LNIWDNTLKTEDMIPVLRDLYNLMGETGMANEVTYADLKFRDSPVAQRIQEFDNIQEIGI

GSPRDSTFIFLKNFPINRFILHSRHQHQKPRATDFQSAASPVASPELSVGDAVTPPFLWT

GVFQAKKNIEQLTGVEKNLSLQLDISANISKEKDLIQSNHSFALKKVATKLCRELTSIKQ

APDSLSNLVSDHACKPCPENWHWHRDSCYWKTSVLNLDESRKVCAERNSSLVKIENKEEL

GTDLKVVGQKQEGPGGPVGIIRGELQVPPPASNNADKVLANRSLLLQLGGIPQNVIGTSQ

TPKTSSTNKLFVKERTKKKFHSSVFSDVPVHSVWRLLSLLLLILCLIQLIGLVGFGIRLG

RDSRQLAAGHLRQERGVPALSAPSEEPWDSLTYDLYYSGKMDHNPRAIPAVLTDAAFSNA

SLSLKIPLAPTDSFLFRGICRSCFKQKEFLLSQNSKLSADLREVTTKFCRKLMENKTAAL

DTAVPDTPRRESLRSAGKHNPRYLFLQYFVTAKIERYHWMKEEVTYTELSFHKTDDVEKV

LQMDLTEKPEQPAPRSLRSRVPLGQLPMCLLLLLLLSGLIVLGILYISLYFFSQNQTVSP

VKRDGNGLEPAVIENLRSGLPGQKERSFVTRGNMVLDDIYICSHAHIAGSSWEAMGEARM

VPDSGDGVDGVRFVDS

>CLEC_like4

MELTDPRAENMEDEDEDGYIMMSIQQQTSIKGLAGSERVIRQPNHVAIQDGNVSETLQQL

ARRLCQELVSKTKGHKCNPCSSLKYHQGNCYLLSHRNRTWEDSRTYCASKNYVMLKVDNK

EELAYINRNTNKIRWIGLSRRAIDSPWMWEDGSVLATDLFQISGDGEANRHCAFFHNGKI

QAADCQESYPTLFPSLAKEGFKACWPSSSDWELHVGSDYLDLHSAWHTARTKQNTTVIIE

KQRGSVERARAWESEPRSTMQDEDGYIMLDFKSRIHATSKGPSGAPGLPPSWRWMTLALL

ILCLGMLIGLTVLGSMCFWGTSGPKGPHKSSWGLGASDVPIFQKETLDRFIVAADLKVAN

IHSPASPTDSRQQALLQQITQKYCQELSSKPGGHKCSSCDHNWRFHGGKCYGSFKNNKTW

EESKKYCDDRNSTLLKIDTQEAWELNSLSVSSLIFLVPTLCRRLSWILGARKEENFIQGR

PDFTRWIGLSRPSSGGRWTWMDHSALTDNLFELLGDGDEGKHCASIRKKKISTTFCRELH

YYICEKVGIVKVDQLV

>CLEC_like5

MSVEDVYILKELAIIVMINNIYSSNSTESSPTLVLGTWWRLLTVMSWLFCLPLLASTVIL

SLKVIQASELIGKQEEILANLSLQQRVCSERLQVCQVQIQMSTSPESNCSLCLEPWVMNG

ESCYLFFDGWKNWASSSEFCVQEKSELLKIGSKEELHLVQGSAQSAPGLPPSWRWMTLAL

LILCLGMLIGLTVLGSMCFWGTSGPKGPHKSSWGLGASDVPIFQKETLDRFIVAADLKVA

NIHSPASPTDSRQQALLQQITQKYCQELSSKPGGHKCSSCDHNWRFHGGKCYGSFKNNKT

WEESKKYCDDRNSTLLKIGHSRSLGAEFLVSVIINISGAYAVQKALLDLGSTQRRGRPDF

TRWIGLSRPSSGGQWTWMDHSALTDNLFELLGDGDEGKHCASIRKKKISTTFCRELHYYI

CEKLPATPPLRPFADSAILPEPPASLIVPDLHLGRQCGYLEAMQDQGTYDSLYWVTPDPP

PVTRPSLKTALRTVLGTYSSNSTESSPTLVLGTWWRLLTVMSWLFCLPLLASTVILSLKV

IQASELIGKQEEILANLSLQQRVCSERLQVCQVQIQMSTSPESNCSLCLEPWVMNGESCY

LFFDGWKNWASSSEFCVQEKSELLKIGSKEELEIKTASPKWDKDCVQPDLLVSTLVLSTV

PGTQTNPQIDFSHWLLRWMFSSCFKSFGTIMAYWREHGPGSQKDWVLIPKSSTCLLCDLG

PTALMYIAVMYLFAVMSVSSCKLILGRAFINRNIERKKTGSSWSYWVGLTQDKCYGDWRW

RDSTVPSSDL

>CLEC_like6

MGMKIVSLTWDNLITLYLPQRLEQCSAHRQSCLDSSMEDEDGYTILSPRTRVFAREPAAS

GKALNHLAPSYLASLLSYRNPAHRYRSSDDNLFTYFDLVHLPSNLSPMSCLWPGTPSLFM

SESLPAVSPRWRLAAVTLGIVCLGLLGAIGILVFQALRPVNGPCSSEMSKNHIQPSKSPP

VRKRDSTKAPETITMVPEPRSRICPPNWNRNGDSCYLFRYTLDNWNRSKMFCESQRSHLL

RINSHEELVFIQHLTSNNSQMSVWIDLTSRSDGSWMWGDGSVFSPHLFDIRQTNSLNRCA

WIHENLVFDALCSSLAYSICEEKTVNSLWAGTASTNSVRPYSPKLLVSSSAGRQAEGNGG

DVPLPRSGKPVHRSVHRSPVQMRAKYSSTQDMLEDDGHTTLSLHSRTSSAARSPEPSGPD

SIPSFLQPSDGGLEKVKDEKKVEEEKEEAEAEKEEKDEKEEEGEDEEEEDEEKEETGAGE

GRSGRGGGRGGAGGDTAVKEHCSHLGRVAPSRAWRPVALTLLIVCLVLLLGLGILGFEFF

QVSRLSNAQRTAISQQEERLGNLSWQLQDLRAQTRKLTGTLQQVAGRHPGGDILEAGGDA

SLKGGGEDRGRDVDLRVICVEMVVEAVRANEFTKGELVIPLSFSKFFYSYWTGLSRNGSG

QPWVWLDGSPQASDLFQVVVDSDSPRSRDCVTILNGKTFSKDCKELRRCACQRAAELDLG

NLSLILPPVCCVTLLCITLACGFVPFIHPTLSPIADEGTEKFSDLPKVTQQTFGKDGIRT

HDL

>CLEC_like7

MGMKIVSLTWDNLITLYLPQRLEQCSAHRQSCLDSSMEDEDGYTILSPRTRVFAREPAAS

GKALNHLAPSYLASLLSYRNPAHRYRSSDDNLFTYFDLVHLPSNLSPMSCLWPGTPSLFM

SESLPAVSPRWRLAAVTLGIVCLGLLGAIGILVFQALRPVNGPCSSEMSKNHIQPSKSPP

VRKRDSTKAPETITMVPEPRSRICPPNWNRNGDSCYLFRYTLDNWNRSKMFCESQRSHLL

RINSHEELVFIQHLTSNNSQMSVWIDLTSRSDGSWMWGDGSVFSPHLYTEKLFEIKDHGY

QLYLIVFSPVLRKQSVNIID

>CLEC_like8

MEEALSTVLCTRGGKVWEKTAFGKEVEKFNFGYAEFEVMMGYPGLLGGTRSSLGAGNSKT

IMAVGFYNFANFLWRYKRKLSTNVAEGVCVTQLPKMNQRKSVDLFASIPLILLVCISAGV

SILACAYPLDMEDDVQYAELKFNMPEEKPKQKLPEKKAEDSSSSSPRWFLAAIMLGIFSF

ALLGAMVVLGLKITSSVKWGLTVSLTWDNLITLYLPQCLEQFSAHIIHARGLRDRQVENY

THLEGKTARFTDQKDVAGASPQDWQEVENLRRKLENITEEHRALLLQNEQLREALKKAKN

YTGPCPQDWFWHGESCYIFSSHFKNWKMSQENCASQGSQLLKVDDQDELEFVYRAVAHSR

NPFWLGLRRSDARSRWVWEDGSTPFVGL

>CLEC_like9

MVPDQAYQDRVGGETGLVPSMAPEIVYAELRFKMDNEKLGAPPAPAPQKATPPPLRPRLP

ILQMALLLLLLLLLLLSFLVAFVVFYRRSHLCQEDRSLKSTRILSEFVCVKQSSQSPVFS

CSYIETHQETEDLPSPHQLPQNQTTLDCINEGSEMEGRSWDCCSRGWRPFQSRCYFISAD

KMSWDQSQQNCHQMGAHLVVISSDTEQKFLESILDNKAAYFLGLTYLEVSKDWRWVDQTP

YNKSVLVCVQPEYLVPAPTLGTSSQITPVIIHTYSVTTKEKYISNF

>CLEC_like10

MHHVGEASQTPVIPKPGVTVFEFGKNYGHSIEPNPSIPVQGPSPYPSSLKTQIVSEQSIS

TTPHTTTTIINNTITNHNPIPHMLRQGSSDLSACPPVTQHGFAQLCKDPEGLAPLNHTQV

SCLRERSQVEGRMWSCCPQDWRPFQTSCYFFPRDVLPWNEAQSKCLKQQAHLVVINTEAE

KNFITQGKPLNSSIHLGLKKRKKERQWRWEDKTPYNPADTFGVEGDPQEGDPALGAREER

CAVLTLRQTSSRWRWSGTDCVDATAHWICEMPRRSF

>CLEC_like11

MSPPKGAGPQNRNFNGHFPSRLSGLLGRGCCCPPWSPWAAATVPVLLLSACFIARCLVTQ

RAFSQFCEEEGTLLRLNDFFSEISCYSSGSGSIRDCCPRGWKHFQSHCYFFSSDTLTWSS

SRLNCTGMGAQLVVINSQEEQEFIFQSKPRRREFFLGLTDKHVEGKWTWVDGTPYDPSRS

FWDLGEPNNIVGVEDCATTRDSPNAKETWNDVTCFSFHYRICEMPAQTLSAGKKGM

>C3AR1

MGTCWGHRMGRMSMPWDNTSSPDSPLQRGHSLPQLLSMVILSFTLVLGLLGNGLVLWVAG

WKMQRTVTTIWFLHLTAADLLCCLSLPFSLAHLALGGHWPHGQLLCKLVPATIILNMFAS

VFLLTAISLDRCLLVTRPVWCQNHRGVRVATAVCGAAWVLASVLCLPVLLYRETYVDGPL

WRCGYIFDHHASKDGLEDNDLLGFSNSSPPTPEMDDPGTLDIPLRARDALLTGSVWLLGP

SLPWGPLDTEAGHPSAIPNPDLSLDPGGPRGDLLPTGMPSQSPDELDLVMDSWDELIKIY

TAQSQVPGPLVAMTLTRLSLGFLVPLAIMVACYSVTVIRVQGSRFAVGQTRTFWLAARVV

AAFFACWAPYHLVGVLSLLATPNSPLDHALATWDPLTHALASANSCINPLLYALAARDFR

VRARLSLRSILEAAFSEGLSHFSTCPPSQSDVLSIDPLGPEV

>NECAP

MAAEAEYESILCVKPDISVYRIPPRASNRAYRASDWKLDQPDWTGRLRITSKGKVAYIKL

EDKVSGELFAQAPVDQYPGIAVETVTDSSRYFVIRIQDGTGRSAFIGIGFSDRGDAFDFN

VSLQDHFKWVKQESEISREAEKPDTHPKLDLGFKEGETIKLSIGNITTKKGGPAKPRPVG

AGGLSLLPPPPGGKISIPPPASSVAICNHVTPPPVQTSSQGGGEAGTGGLSRGRRVSGPQ

SVDPSPGALTSLSCRPPTHGHSDILLDLDAPAPSTKAPAAIPAAPDLWGGFSTAARPSPV

LIQQGDPPFNTTTCSVQAGSPKVPIANGFLAQGPDDTQIYISAPVLSPSLQARISSCLWD

VSTWMSARHLKLNMSKTELLIFPPKPGPLPDFSITVDGTTILPVPQARNLGVILDSSLSF

TPHILSVTKTYRFHLYNIAKIRPFLSTQTATLLLRALVISRLDYCVSLLSDLPSSSLAPL

RSILHSAARLIFPQKRSGHVTPLLKQLQWLPIDLRSKQKLLTLGFKALHHLAPSYLSSLL

SFYRPPRTLRSSAAHLLAVPRSRPSRRRPPGHVLPRSRNALPPHLRQTDSLSLFKTLLKN

HLLQEAFPD

>CARD9

MSDYENDEECWNTLEGFRVKLISIIDPSRITPYLRQCKVINPDDEEQVLNDPSLVIRKRKV

GVLLDILQRTGHKGYVAFLESLELYYPHLYKKITGKEPTRVFSMIIDASGESGLTQLLMNE

VLKLQKKVQELNLLLSSKDDFIKETRVKNSMLRKHQERAEKMKEECQAFSQELKKCKDENY

NLAMSFAKQSEEKNAALMKNRDLQLEIAQLKHNLMKAEDDCKVERKHTTKLKHAIEQRPSQ

EVMWEMQQEKDLLLVKIQELENSIREGKREKNSLYITVLEEDWRQSLLEHQEKETTIFRLR

KDLRQAETLRNKPGMLLIGREALSKSAPFPTVRGGGEPAPGSLVRPSGPEAEDEGGAGAEL

SQGGAARSIVPAVMFIERLLCVEHLVKHGQGKGQEHLDNDPPCMEEKEMFELQCTTLRKDS

KMYKDRIEAILQQMEEVAIERDQAIMTREQFHLQYSQSLIEKDGHRKQIRELGEKCDELQL

QLFTREGQLLSMESKLKRLQLETPNLSLDLEEMSPRNSQEDKTLTYPTAPHSVQSERPSHN

LDRGDLQPAAASPVPLHRRLSDRSETCAPIVGRSGEGPAFSFPKGKQGQCTARGLNTEFKV

LHTKGKVSNGEPPDKERRRMKDNFEHYRRKRALRKNQTSRHLGEVDRDNTTGSDNTDTEGS

>SNAPC4

FEGTRNAVELRKFWQNHEHPSINKAEWGEKEIEKLKEVAAKHSYLNWQKIAEELGTNRSP

FQCLQKYQTYNKDFKRKEWTKEEDQMLTQLVGEMRVGSHIPYKKIAYYMEGRDSMQLIYR

WTKSLDPNLKKGFWTPEEDAKLLRAVAKYGERDWFKIREEVPGRSDVQCRDRYLKRLHVN

LKKGKWSPSEEEKLIELVEKHGVGHWTKIAAELPQRTGSQCLSKWKIMIGDKKKQRNRKR

QRKSRRGPRGGQHVPSSSEESEIEFTDNSEEEEEKKSDQEKEPAVPSYTVPNIDLWIPSG

EPRATPRGAVTIQTPRPRARTMGSGSGGATPGPTGDAGEGQPRGQGASMDVSATPKGFPC

ARSTDIPLENPEPQGNPEPLENPEPLEKEDPGSGKQVLKLTPDEVKKVLRSNTYILRRKL

QEKLKKPRLPTSSVMPQVSAGMSGDQVLQVWENTVEKRLQRLKVGLRRNIRRSNLDRRLL

LAVTPWVGNVTLPRFQNSRKGLVLQTKVKKRPLQTVLLTSTPMFTLFIQLFQIDADGCMK

VIQERKANQVEVVQVEPGNPEQLRQASCSSQDTSGCQPQKDPAKGNSRRRSAPRVEDGQL

ARDGKVTAQAGAPSAPQPQRPKPKTVSQLLREKRLRESKATMPNPVILAPQVLVSQPVLL

RHPLQPVIPSGPATATLAQAGPPVKPPAPSPTSTPISSASLRALASGTLAVASENSSGQV

SSEASRAVGVTEDPKAKEEGKSSAPLTVVIPPPGDGRGRVQDGGSPQPGVPPNPSPGPGQ

LLAISAPGGFTLARGAPSGGKQGPPEASPLLPPAPVRFQQPRLLPALAVRPSGPQGGPSE

ILPFTWVVTPQGLVPMSVQAFVGLHGPSKTVGDLGAANCQGPVGTTLVPPGGPTLRLLLP

PATTPLAVTSSGAPGTNTAVAPGSSLSSHRVAGEPAPPTVELSHSASLPSAAPPDREVSR

SPEEVSAAEKSQPPQPTPSPQSQNPNGGQRAEPLGSHGLPASATKSPSSGEGEAPSSQSH

MPKDLPATQVGRCPDRTGASGNSAPTTAPLPARVGKDRPIAPRPEATPSPGGHPAPADPP

PRGHPPPGGDRPPGPVAPDKNKLDLTLISLEEEAVVREWMKGKRGVHVPPLKNNLPYLPP

SVYNLKTLSGLLREKKNLERSAASLVAGDGGPAGSELSPEGLEAIRALVRERLKDNPAYL

LLKARFLAAFTLPAFLATLPPPGVPTTLSRRPEESEEEDEDDGEEEEKDLTDEESGEEDH

PPGEAQPGPEAATSGQEAKKTEVKGRLGVSWSGSEVTAAKKRNGGAPGGRGPERDRCSGE

PSEMPGLRFLPYREDGEPVSCLHLHLRVIFVVFPMTDPEETLMDLLGTEMKPSPPVVNCE

FGFPVRRHPIKEPPCRSGPGYNEVKSGPSGSALKQKLPQQAPPSLEFLHRLLHFLSTPRA

KDSPDTLKCACKAESGQRWWLPVGLKGHRGMLSFPEALRINIRARWRRPLQSILNRLCKT

LDFFVRSRHRTPCPGPASVGCHLKRSPSQEVHWAPV

>MALT1

MGSVNSQVVPLKTAPALLWNCVQVSDAGFYVCRVNNDTTFEFSQWAQLDVCELMESSYGNLDGFS

ESKLQICIEPKPQKLIPGDTLVLHCVAVGSPIPHYQWIRNGFPIADETKKLFMVPFVDKEHQGTYWCHI

YNDQDSQNSKKVEVIIDELTDYGQNAKDKVALLIGNMSYLNHPKLKAPLVDVYELTNLLRQLDFKVVSL

LDLTEHEMRNAVDEFLLLLDKGVYGLLYYAGHGYENYGNSFMVPIDAPNPYRSANCLCVQSILKLMQ

EKETGLNVFLLDMCRKRNDYDDTIPILDALKVTANIVFGYATCQGAEAFEIQHSGLANGIFMKFLKDRL

LEDKKITVLLDGVAEDMGKCHLTKGKQALEIRSSLSEKRALTDPIQRTVCSPESLVRNLQWAKAHELP

ESMYLEFKCGVEIQLGFAAEFSNVMIIYTRIIHKPPEIMMCEAYVTDLPLDLDVDPKHANKGTPEETGS

YLEHLIFTVHLSYNYQGMEDTVDERQEVNVGKPLIAKLDIYRGFGRKSCFQTSLMSSPSNLTPISGAA

EHYHSFPNSFPGAFDPHPGNSGGDLPSISCHCSRTSDMVFSSQSTQDYSSQFCKSNIPVETTDELPF

TFSDKLKFSEK

>BCL10

MQEEREEVDFPAALETLRPTLCNKIIAERHFDYLRSKKILTREDTEEISCQTSSNKRAGKLLDYLQENP

KGLDTLIESIRREKTQNFLLERITDEVLKLKNIKLKYLKGLNYSSCLSSPYGATNNLSRSHSDESNGSVN

VNDRDSTVIYHPEGESSTATFFSTVPSLNLPIVEEGRTENAVFSSATLPGPGEPGAPPLPPDLQREDE

ESSGNSSDNLFLPLRSRSGLPR

>MYD88

MASAAPGPSPGLSPGSPPGPSLALDRVPLVALNFKVRTRLSLYLNPQTPVAADWTALAEELDYEYLQI

RHFQAQPDPTGCLLEDWGRHGRQATVGRFLALLAKLQRDDVLSDLGPSIEEDCQKFLLKQQEEAEK

PVQVAAVDSSVPRTAELSGITTRDDPLGQLPELFDAFICYCPSDIQFVHEMIRQLEQTEFGLKLCVSDR

DVLPGTCVWSITSELIEKRCRRMVVVISDDYLQSNECDFQTKFALSLCPGARQKRLIPVKYKAMKRDF

PSILRFITVCDYTNPCTKAWFWPRLAKALALP

>TNFA

MSTESMLRDIELAGEPAGKTAGAQRPGHCLCLSLVSFLLVAGATTLFCLLHFGVIGPQDREQDGPLLP

LTQMLKSYQPSAEKPAAHVVANSQEDKKLVWVGGRANALLEGNVILDKNQLVVPASGLYLVYSQILF

KDSSCPANPDDSPILTHNVSRFSDSYEYEVSILSAIKTPCQGGAKGTWYEPIYQGGIFRLNQGDRLSS

QTNSPEYLDFSMEGQVYFGIIAL

>PANX1

MMPRTQISCFSPSTFSWRQGAFVDSYCWAAVQQKHFSQSDSGNLPLWLHKFFPYILLLVAILLYLPSL

FWRFTAAPHLCSDMKFIMEELDKVYNRAIKAAKSFRDIDVRDAASSVLALNDNGGQSVWEIPENHFK

YPIVEQYLKTKKRSKNLIVKYLFCRVLTFLIILLACVYLGYYISLSSLSDEFVCSIKSGILRNDSTVPEQFQ

CKLVAVGVFRLLSFLNLVVYAALAPVVVYTLFVPYRQKTDVLKVYEILPTFDVLRFKSEEYDDLSLYHLF

LEENISELKSYKCLKVLENIKSSGPGVDPMILLTNLGTIKTDVVDGREGKSGEEAEPETPANSPGELKA

KKWHHQVYFPTLNQLTSLSVEDSRCERPAALFRWLKCVTAVFSFDRSGPEQRDKGE

>P2X7

MSFALVKDKLYQHKEPLISSVHTKVKGLAEVKGHTLPRIFDTADYTFSLPGNSFFVLTNFIETYQRQGQ

CPEKYRLLLSRHSERREMKDETRDRITVQYWAMLDEAHVIFSLLRPALLKSAENFTVLIKNNIDFPAHN

YTTRNILPDLNTSCMFHKTQNPQCPIFRLGDILQETGEEFSEVAIKGGIMGIEISWDCNLDRWFHHCRP

KYSFRRLDDKTTNESLYPGFNFRYAKYYKEGGIDTRTLIKAYGIRFDILVFGTGGKFDFIQMIIYIGSTLS

YFGLATVFIDFLISSYSSECCRTRIYPCCNCCKPCVVNEHYYRKKCELIVEPKSTLKYVSFVDEPHIRM

VDKQLLGESLQDAKGEKVPRPDTDFTVFTKLRPPLPYQAPAPGHPQELQPLNREEPPASDRESPHW

CHCGNCSQSQLPKGNRCLEELCCREKKGPCITISALFQELVLSRPTLQFMLFYRDPLMTLDSDALTR

ELRHCAYKRYIDWRFGSEDMVDFAILPSCCRWRIRKEFPKPGGQYSGYKSFY

b) Echidna protein sequences

>PYCARD

MGSARDHILAALENLTSDEFKKFKTKLLSCPLREGFGRIPRGPLLNMDVIDLTDKIVTTY

MEAYGLELTTAVLRDVNQAEATALQMAAGPATPGPVLSGPAFGEAGQQHFVDKHRQALIN

RVTSVDAILDALYGKVLSEEQYQAIRAEKPSTKQMRQLFEFSLSWDRSCKDQLFQALKVI

HPFLVKDLENS

>TRIM72

MSAAPALMQGMYQELSCPLCLKLFECPVTAECGHSFCRSCLARLPQDPQAGGTPCPSCQA

PTRPEGLSTNQQLARLVESLAQVPQGHCEEHLDPLSVYCEQDRVLICGVCASLGKHRGHS

VVTASEAHQRMKKQLPQQRLQLQEACMRKEKSVALLDRQLTEVEETVRQFQKAVGEQLGV

MRTFLSVLEKNLGQEASRVTGEAGAALQGERRGLASYLEQLRQMEKVLDEVTDQPQTEFL

RKYCLVTSSPAPSLTIVHTSHKFLGPTPSSNRFHTHLGFPSRLQKILAESPPAARLDIQL

PIISDDFKFQVWRKMFRALMPALEELTFDPATAHPSLVVSPSGLRVECMEQKAPPPGDDP

QQFDKVMGVVSHQLLSEGEHYWEGFWLLGFRDGKVFEAHVESKEPKLLKVEGRPTRIGIY

LSFQDGVLSFHDASDPDNLTPLFSFRERLPGPVYPFFDVCWHDKGKNAQPLVLVRQEE

>FUS

MGVIRSSPPKTISRGLYGQTVGVPLPVLEGPTSGSVDVFRLQLWTSLSLDWGCGEFDFAF

ILLQSTPSQPHRAMDLIPPSLGRATRSRAVSLMASRVTVATANRLTLRAMARAATVPPTD

RRRTARGQLTGWVFAYRQCSPNQCSPNQLALPHPLSSMAWSQKDLDSNPGSASLLLGDFG

TPSTPQGYGSSGGYGSSQSSQSSYGQQSSYPGYGQPSASSSSSGGYGSSSQSSSSSYGQP

QSGGYGQQSGYGGQQQQQQSSYGQQQSSYNPPQGYGQQSQYNSSSGGGSSSGGSGGGNYG

QDQSSMSGGGGGGGYGSQDQSGGGGGYGGGQQDRGGRGRGGSGGGGGYNRSSGGYEPRGR

GGGRGGRGGMGGSDRGGFNKFGGPRDQGSRHDSEQDNSDNNTIFVQGLGENVTIESVADY

FKQIGIIKTNKKTGQPMINLYTDRETGKLKGEATVSFDDPPSAKAAIDWFDGKEFSGNHI

KVSFATRRADFNRGGGNGRGGRGRGGPMGRGGYGGGGGGGGSRGGFPSGGGGGGGQQRAG

DWKCPNPTCENMNFSWRNECNQCKAPKPDGPGGGPGGSHMGGNFGEERRGGRGGYDRGGY

RGRGGDRGGFRGRGGGDRGGFGPGKMDSRGDHRQDRRERPY

>NLRP-like_1

MGHIELRKLDEVERSEISVGEQHRFVGRRFVGKKAGREVAVQPYWIFLTQLSLDIKECLV

QCSAHSKRSINTIDDDDDDEGPAIRERYQAQMGEKFRILRDRNSRPGESEALRHRFTRLL

CLPEHRQREEKEHELLATGVDHARAMEIQGQFVEVNALFDPDQERRFQPKTVVLQGAAGI

GKTVLARKVMLDWAEGNFYEDAFHYVFYLNCREMNILSERSLADLISVHLGDSQVPFDKI

MSQPGKLLFIVDGFDELKWVFEEQESDLCHDWRESRPVPVLVSSLLRKILLPEAYLLITS

RLTALDTLNRLLLHPRHVEILGFSAADRKEYFRKYFRDENQATQAFSLVEDNETLFTMCF

VPLVCWIVCTCLKQQLKRGEDLTQTSRTTTALYVSYLTALFPASQDHPGQDHPPGPILRR

LCHLAAEGLWTRKILFDGDDLRKHGLYVSDVSPFLQMSIFQKDIDCENCYSFIHLSIQEF

FAAMFYTLGPEEGRMNCPDPDFGDVKKLLEKYRKSHETGFLTLTVRFLFGLLNEERVKML

ENKFNCKISQEIKRELLHWSQNQCEFLQQGTLFECLYEIQEEEFVAKVMDHFQEVDVCFF

TKMTFLASSFCLKQCQNLKKIKITAVYEEESRQATEPQTDDPENKSSFNFTCWTNLLSVL

STNQSLTELSLRFPQIDDLTMLVLCDGLKQPNCRLQKLINQNLIHLDLTGNNLGDGGAKL

VFEALRHANCHLQSLRLEDCSLTAACCRDLCSALTSNRNLIYLHLGRNELGDTGVTALCE

ALKTPSCSLRSLWMQDCGLTDACCPNLASVLRCNQNLVQLKLWGNDLKDSGVDMLSGSLR

APQCRLQELGLGNCKLTDACCERLSSALGRNRTLIHLDLQCNFLTESGLIPSLLLPVVQK

ATGPMMYQLQNNYSKTPEPASLQDGQSGQSLQLPKEEVHTYSHQNKDLFRKMFPHTVNKR

QRNQSYRHHQERNFL

>NLRP-like_2

MGMKTVSPTWDNLITLEPPQCLEQCFAHTSLVVVGSAGLLGGTAQTLDIGQTPTLPSDGS

EVSPLMGPRGALHFVDRYRGQLIGRVTSVKPLLDLLHGKVLSEEQYRTVLARATSFDQMR

ELFAYLPSWDVDGKELLYQAIWQIHPQLVAELESSLICKMGMKTVSPTWDNLINLYPPQR

LEQCFAHIWLPSPTFHGNCPLKGHHDLLLAKSNGSYSVLILLDLSAAFDTVDHPLLLNTL

SDLGFTDSVLSSFSSYLSGRSFSVSFAGSSSPSHPLMVGVPQGSVLGPLLFSIYTHSLGD

LIRSHGFNYHLYADDTQIYMSAPALSPSLQARISSCLQDISIWMSARHLKLNMSKTELLV

FPPKPCPLPDFPITVDSTTILPISQARKLGVVLNSALSFTPHIQAVTKTSRSQLRNIAKI

RPFLSIQTATLLVQALILSRLDYCISLLSDLLSSQPSEGVSLVEDYREKYLKHVKWKFQY

LEERNARLGEKVALGLRYTPLLLVEEHRNLPQRQHELLALGRPPTSRVPRRVRVDALFDP

DAEGLEPPLTVVLQGAAGIGKTVLARKVMLDWAAGTLYPGRFDFAFYVHCRELNLKRKRS

AVQLIQQCCNDDSIPLSKISQRPDRLLFLVDGFDELGWSSGGWAAEKDDDDDDDDDLFVD

WRDEKPVGSLLAHLIRKRLFPKASLIITTRPAAAEGLRPLLRWPRRAEILGFSEAERAEY

FHRYFPDPGQAERALAFIQENDVLFTMCFVPLVCWIVCTGLRQQMERGEDLAQASKTTTA

VYLAFLSSLLRPARHLPSRVPPAHLRGLCSLAADGILSQRVLFREADLRKHGLPEDGVSA

FLHVDVFEKDADCETLFSFVHLTFQEFFAALFYLLGPDEARPGAGPGPVPDVRTLLRHYR

LCDTGFLTLTVRFLFGLLNRERVADLKEKMDCVVCPEARPALVEWINASAQKEVLPGPTL

QWLYCFYEIQEVDFVERAMGSFRKIDVDVRTRMDQIAVAFCLRNSRNLCSIELMRFFNVQ

EEEAAVAAAAAAAGTPKAAAYQGSQDWLQDTFCESLSEASAKNRDLKHLALKNKTLRGRG

AELLRKGLTHPNCKLKSLRLVNCVLPPDSCRDFSSIVSTNQNLSELDLSSNALEDSGLSL

LCEGLTHPSCKLQTLWLQSCALTSACGPALCSLLSTNPNLTKLDLFNNALGDAGVRLICE

GLKNPNCKLQALLLRHCAVTLNCCPDLSSVLSINRHLSELDLSYNSLEDAGVCLLCEGLR

HPNCKLQTLRLQRCKLTSRSCPDLSQMLGVNRELREITLHDNVLGDAGISEIWEGIQQPS

CGLKILRLGKTDLSEKMKKEMKTLQNVKAELKIRYWFRGSQGGRGLLVRSPSPEHVTLAL

SGEG

>NLRP-like_3

MTCSIRSRLAEYLEELRDPELRKFKFHLEDLAPAAGWAPIPWGRTEKADSLDLAHLLVAH

VGERGAWELAVHVFERIHRKDLWERARAEAVVRDLSVSALGCQWEQRRRAEHLVEPHEAE

ATRDPRDVYREHIRRKFRFIEDRNARPGECVNLSQRYTQLLLMERHPTPQDAAPESPTAR

LEAAGAPERQPGPVQVETLLEPDASWPEPPRTVVLQGAAGIGKSMLARKIMLDWAEGRLY

RARFDYLFYISCREMSRVGRSSLAGLISGRWPRREAPLADIVRRPERLLFVIDGFDELGR

SPRRPPPGRRAGGWKAKLPVASLLGGLLGKDLLPEASLLITTRPAGLARLQPLLEQPRHA

EILGFSRAGRRDYFHKFFGDGRRAARALGLVRDVEALSAVCFVPLVCWIVCTCLKQEMER

GEPPGQPSKTTTSVYVFYLLSLLQPDPGGSGPRGRPVLAPLCSLAAHGVWARKVLFDEGD

LRRHGLAGSDVSAFLNLSVFQKDIHCQRLYSFIHRSFQEFFAALFYLTGGDGREGGRGSR

HSVTRLLEHYGRSETSYLALTVRFLFGLLNEENKSYLERQFGCPLSPTVKGELLAWIEAR

AQIGGRTLERGDAGPLLLLTQEEGFIRRALDPIQVVVARDLSTKMDHVISAFCVGNCRNA

SVVHLGSVEFSSEEAAEGQGPVGTEGTHLGDQPLSPVERCWLPDTYCEHLSSALRTNQNL

AELVLDLNALGNQGVKLLCQGLGHANCKLQNLGLKKCRFSSAACQDISSALSANQNLVMM

DLSNNALGDVGVKLLCAGLRHPKCRLQSLQLKKCYFGWAACEDLSSVLSTNPHLMELDLT

GNALGDAGVQLLCVGMKQPSCRLKTLWLKICHLTRASCVELASVLSMDCSLTELDLSLND

LEDAGVRLLCEGLGQPKSKLQKLRLGICRLTSAACGAISTALGPNGHLKELDLSFNDLGD

GGAHQLCGGLNHPNCKLQKLWLDSCCLTAIACESLSSVIAKHQTLTKLYLTNNALGDAGV

GLLCERLNQPTCKLRTLWLFGMELKAETQNTLAALRRTKPHLDIGS

>NLRP-like_4

MALSVRDLLTQTLEDLLETEFRKFKGKLCEIPLWDPPGGRGGAAGIPRGALEKADRLTTA

DLILSYCGSGSALDVVARVLENIQQRELGNRLRERAPDIGSSVKITGKLDTEKPRALPKR

TSGAAVCEGNCGSNNSDRGPCSNYNYGGVDKHLLNVKHRPKRRGRAKTIGPEAVPSHTGL

TIEGGKEDGDLIWKMGMKSGSPPWDNLITLEPPQRLERCFAHRKISQEMYRSEIRHKYER

VKDYNSLPGAWRSLEQHYVAPLIIRRCRPAREREQELLSKGPRHLELLRLCGEGGSARVR

LAHLLDGPGGRRPLTVVLQGAAGIGKSYTAHKIILAWASQRLYHDRFDWVFLFNCRELGV

EPRPRSLADLVLTDCPALGPHVGQIFSCPQRLLFLLDGFDELQPPRSPEEDEREEGEEGG

EEAAGRRAEAAVHRRRPAAATVRLLLRQRLLPGCLVVVTTRPSALEQLEGCIRADVHLEV

LGFLEPERQAFFGRFFGDAKRGREAYEAVRGNEALSTMCFVPLVCWIVCTVLHKQLEKGQ

GLDGLQTTTQVFLHFLSILLRFHRRPRARAPADSLLEQLGTLAVHGLLTRKVMFDREDLE

AHGLPTAAPPTIFLSAVLRQGVTVDTVYSFGHMMLQELFAAIFCFLPGRGGSARAGARPP

PGGGAAGGERPPAPGDPLLCQAMLRQLLPGRAALTPSPEGALLSWVQRSAASPRAGHPPF

LLELLHCLYEWHCDGLVGQVAGQLNVRFLLFPLKRSDCLALAYCLGCCPSVSCLHLYSCG

LDQGDIRLLLPALSKCQTLHLGLSDIPSGLMREIGCGFSPKQSVTSVLLQGLGPNNSSSQ

KETVFKVSALWGSRPCRLCTKRRLRCLQANRVGRAPRPVERARARESEAVSSNPRSANCQ

LGDFGQVILLILINRIY

>MIS12

MSVNPMTYETQFFGFTPQTCLLRIYVAFQDYLFEVMLTVERVILKRLEAAPGGGEGGGVS

PVQIRKGTEKFLRFLKERFDGLFATLETVLLQLVLRVPDHVLLPEDRSHARHPGGREELA

RLREEADRLRGRYEAEARAGRALMAELEEQRAARAQLEKTLRWFDGLEGAWREHGGGDPR

ESLAFLRRGAHRLRDVVGDVESKGRRLESA

>PGGHG

MGLTVFIPILQMRSPVPPPLPSLTLNDLATYFIRKINTVRPSRCKLTDAECRDLAEAVAS

SRTLMDLELMGNKLARTSTMDYGGVDDPAVFTSPTLPSDPRFLATLTNSYLGTRVYRDIL

HVNGVYNGALGDAHRADIPSPVNVRLEAPEGVDVSQSFTLDTRTGTFLHVVETREFTATH

RIYAHRALTHLLAFSVTVRRATPQGPPVTVRLRSDFTPKSQDLDLHLGPDFQGARLAAFA

CYFQVSNGSAGMFELRVDRPERSPEEGACRYLCGRTLSPEVAGGPQPTVHMLWTPAPPAL

TLPEAQREATWQFLAAVAEAEDEVRHLFEEGAALLRAGTLYPAHVEAWGALWGASGLHLD

GPLSLQRAVRGCLYYLLSAVPSPAPGVRDPFHGISPGGLSNGSRGEDYWGHVFWDQDLWM

FPNILLLWPEAARAILQYRVRTLSGAQANARDQGYKGAKFPWESAATGHEVCPQDIFGTQ

EIHVNGAVLLAFEQYYYSTRDLQLFKEEGGWDVVSAVAEFWCSRVTWSPEEQCYHLRGVI

PPDEYQTDVDNSVYTNVLAQNSLRFATSLGRDLGLAVPKEWLRVAENLKVPFDPKRRYHP

EYDGYHLGDLVKQADVVLLGFPVPCAMDPDVRRNNLEIYEVATTLQGPAMTWSMFAVGWL

ELKEPQRAQQLLNKCFDNISEPFKIWTENSDGTDAVNFLTGMGGFLQAVLFGYTGFRITK

DCLRFDPVCPAEVRHGQVTGVSYLGNKLNFSFSEEEVTVEVTWAQSQAPALEAMLETSGQ

CLALPQDCEPTVG

>PSMD13

MPRAAGAEGGRFPACPARRGTGSECAFPVGVAGEGLGNGRAPSGPPPQGIPRVPTRSLRP

VLGAPRGAPPWCPPPAAAAAAMKDVPGFLQQSQSSGPGQAAVWHRLEELYTKKLWHQLTL

QVLDFVQDPCFAKGDGLVKLYENFISEFEHRYDPPCVLCPGSRGRVRCRTDPSVALTFLE

KTREKVKSSDEAVILCKTAIGALKLNIGDLPVTKETIEDVEEMLNGLPGVTSVHSRFYDL

SSKYYQTVGNHASYYKDALRFLGCVDVKELPVSEQQERAFTLGLAGLLAEGVYNFGELLM

HPVLESLRGTDRQWLIDTLFAFNSGNVEKFQALKASWGQQAIIVAAQAGEPVHPELAAPR

SVQGTFLLYCPPPSTWNSAPHTPDLAANEALLLQKSQLLCLMEMTFTRPANHRQLTFEEI

AKSAKVTVNEVELLVMKALSVGLLKGSIDEVDRRVHMTWVQPRVLDLQQVRRRAPLCLGA

GGPLGIIIIIIIIIIIMMAFIKRLLCAKHCPKHWGGYKIKGMKERLESWCTDVKSMEMLV

EHQAHDILT

>MYADM

MPITVTRTTITTTSMSPGGGNHTIVGSPRALTTPLGIVRLLQLLFTCVAFSLVAHIGGWF

GSMGDWCMFSWCFCFAMTLVILLVEVGGLQPRVPVSWRNFPITFACYAALFCLSASIIYP

VAFVQYLHKGEQKDCGIAATVFSILAFLAYTTEVCWTRARPGEVTGYMATVPGLLKVVET

FVACVIFVFISNTNSYERHGALKWCLAVYCIFFILSLAAILLCVGECTSWLPCSFHTFLS

GYTLLAVLAYATATVLWPLYQFSSRYGGHSRPSYCPQNYGNPCLWDRLLAVAVLTAINLL

AYLADLIHSARLIFVHV

>EIF3F

MAAVPEATTTTTTTTAAAPDPAAAAAAPSPAVPAVPAAAPSAAPSVPVPGGPFPGGRVVR

LHPVILASIVDSYERRNDGAARVIGTLLDEGTEAQRSEVIIMTVFVKRLLCAKRCTKRRG

GYEVIRLSHVLALSLHPHFTDEVTEAQRNEGTEAQRSEVRIMTAFGKRLLRARHCTKRWG

GYEVIRLSHVLALSLHPHFTDEGTEAQRNEVTEAQRSEVIIMTAFVKRLLCAKHCTKRWG

GYEVIKLAHGGLTVFIPILQMRELRPREVRHCTKHRGGYEVIRLSHVLAHSLHPHFTDEG

TEAQRNEVTEAQRSEVIIMTAFVKRLLCAKHCTKRWGGYEVIKLAHGGLTVFIPILQMRE

LRPREHLQQCFGHRTIDKHSVEVTNCFSVPHNESEDEVAVDMEFAKNMYELHKKVSPSEL

ILGWYATGHDITEHSVLIHEYYSREAPNPIHLTVDTGLQNNRMSIKAYISASMGVPGKTM

GVMFTPLTVKYVYYDTERIGIDLITKTCFSPNRVIGLSTDLQQVGTASARIQDALSTVLQ

YAEDVLSGKVSADNTVGRFLTDLVNRVPQIPPEDFETMLNSNINDLLMVTYLANLTQSQI

ALNEKLLCL

>CASP1

MGRNLDLRSGQWEVAVLKAVPASPGALVVNLRIPQVPDQAKDQVCQLKERGPTCLLPLCQ

YCNTRIMNKELYNSLAIRSRNKFPLRASSYPNIPLILGHRKALSSPRLMEEQTPPLIFFP

GKGPLLGSCERKFQNSLTEQTGNAAKAVSLSPDLPAPAPQEAEGEWLRSSMAQWKEHRIW

SQRSRVQIPALPIVSCMTLAQLLKDRWCLIIESLTHGMISGLLDDLLQTQVINQEEMDTV

REENQRPAEKSRALLNSVIPKGDLASQIFIDALCKRNPFIAAKLGLSTGLVIPHSSRDMI

LLESLLTSERTEAQMGHFSSKPRIVAHHGLTPLFIRVASILVLPDHTHGAEEKFQLISRG

KQIQNSLSRFHSPKPTPEDRQGQSSNTQLVAHSPVYPAMAASCSAPQALQAPKALTESHP

DGPVEILRLCTSEEREKLQKENAGEIYPVLNKTGRKRQALIICNIKFDELLERVGAELDI

KGMKKLLEDLDYNVQIERNLSATEMESTLKLFAQQPEHKFSDSTFLVFMSHGILEGICGT

KFKTQDPDVLYYSTIFRIFNNLNCPGLRDKPKIIIVQACRGENEGMALVSDSLGASAYSS

QDLEDLENDAIHRTHVEKDLIAFCSSTPDNVSWRDPKTGSLFITQLIKCFQNHAWGCDLE

SLFMKCWTFSTAAFPQTGVDHTTYIPIDQSLQRAQHCAKHLGKNNTIELVDAILDWIQAN

GVNIHGQSSGNLDICGKQCELLWTGDISAKFALYSLKLLVQCSAPTIYLPHLDHQADNII

RAIIHQYHSKVEFGNRLEQCFTHRAEHGDTKQQQEEEEEEQQRSTCLSLAQKARCQPCHQ

NRHTEGKGRGMGRIRTLLIIHLPDRSIAIARINRFSSNLSQNSLQLSSVELPVILFYPLL

FTHPLPKNNNGTFCFTWQLDSPEAPSVRGRKNIIEFPIGDGGTEKLSDLTKVTLQSLGKW

ARTAAGNAYVEGLISSSRASRKNHTFLLPDATPCSEDQGTQ

>GRIA4

MGGWRGGRGGEEGTGSVWEGLLEEGWHRAFWAEKDTWTAAAPAAAPPPPAAASCLPLKAC

DLSARLYNMLNSLDSPYCKNTGFPKVLPPQFHQCCKQTKMVQECIRRSCQLERSRIYLIR

KMEIKTVSPMWDVQPEQLVSATVLRTGLAAYIYYTPSTVLNAGAKKQQGLMDRAWIQLCH

SSGYSILQAIMEKAGQNGWQVSAICVENFNDASYRRLLEDLDRRQEKKFVIDCEIERLQN

ILEQIVSVGKHVKGYHYIIANLGFKDISLERFMHGGANVTGFQLVDFSTPMVTKLMQRWK

KLDQREYPGSETPPKYTSALTYDGVLVMAETFRNLRRQKIDISRRGNAGDCLANPAAPWG

QGIDMERTLKQVRIQGLTGNVQFDHYGRRVNYTMDVFELKNTGPRKTQRVLGWCASRGCL

KAEQEIETPQKFGEDGLRGTQHRCQPQRSMNNKQNNLAEGCLSGLKCPLEEDTRQLENIG

HFWDATNPTDPIIPVHHYFLGQSGQDKILARTLLRRRLQILLTERVQNPSAALAHSQHSR

HDLGLTSITRQVQGTASQPLYFTDLPEAFDNINKPEVGYWNDMDKLVLIQDVPTLGNDTA

AIENRTVVVTTIMESPYVMFKKNHEMFEGNDKYEGYCVDLASEIAKHIGIKYKIAIVPDG

KYGARDAETKIWNGMVGELVYGKAEIAIAPLTITLVREEVIDFSKPFMSLGISIMIKKPQ

KSKPGVFSFLDPLAYEIWMCIVFAYIGVSVVLFLVSRFSPYEWHTEEPEDGKEGLSDQPP

NEFGIFNSLWFSLGAFMQQGCDISPRSLSGRIVGGVWWFFTLIIISSYTANLAAFLTVER

MVSPIESAEDLAKQTEIAYGTLDSGSTKEFFRRSKIAVYEKMWTYMKSAEPSVFTRTTAE

GVARVRKSKGKFAFLLESTMNEYIEQRKPCDTMKVGGNLDSKGYGVATPKGSPLRKWKHL

TTYIIPQFPKAEWPQNYKAYAAIPKGGGLRRTNQLQDQEQGSKPSVKYGSDTKWQYLLST

YCVRSVVLSIRGIQYELEDTIPALKDLTI

>PDGFD

MLLALGHVASHVRYFQVSLPKESSGGNGWNQLFQQILLHLYSKERNSHGSLNRNSRNPDR

CTLGPSIDTDTNMKVVIWQKFIDSTGTVGSRKQQYLYRKEETIHVTGNGCVQSPRFPNSY

PRNLLLTWRLSSQGNTRIQLAFDNQFGLEEPENDICRYDFVEVEDISETSTIIRGRWCGH

KEIPPRITSKTHRIKITFKSDDYFVAKPGFKIYYSPVGIAYHPPSVTDPTLTADALDQTV

AEFDTVEDLLKHFNPETWQEDLENLYLETPRYRGRSYHDRKSKVDLDRLNDDVKRYSCTP

RNYSVNLREELKLSNVVFFPRCLLVQRCGGNCGCGFPNWRSCTCSSGKTVKKFHEHAILF

PDIHSVKKNTISEQPTLPMLKFSGLKSTVSGSTLFGISGDEVTEAQSSVVTCPQYTSVRA

GIRIQVLLSPRPVLYPLGNAASLQEERLPKPRTGHPPRPALLLVRHSLLQTEGLVQSWQW

GSNCSNRSICSDVYGEGELEAA

>MTMR4

MAICRMGVRRMQWSGEGRAGTLGIVTVESEVAPAKGAKVKAKGNASHTGDSGSCFPHPAR

TLRSSAANLLTVPRSRLSRHRPPAHSPALVEGVTVRPSVRPSAGDMDGSPEPRRRRQIRR

FLEDPEEAELAQFVQEFPGGDGGGGGGGCRRPESEEPSCRDPEALPAALEPSPEPDPRPP

ARPWPPDGHQHISAPAPLSPLTRPRSPWGKVDPYDSSEDDKEYVGFATLPNQVHRKSVKK

GFDFTLMVAGESGLGKSTLINSLFLTDLYRDRKLLNAEERITQTVEITKHSVEIEEKGIK

LRLTIVDTPGFGDAVNNTECWKPLADYIDQQFEQYFRDESGLNRKNIQDNRVHCCLYFIS

PFGHGLRPLDVEFLKALHQRVNIVPILAKADTLTPPEVEHKKRKVRGRGWRRRRKSGRKG

RERGRAQQPSPRQIREEIERFGIRIYQFPDCDSDEDEDFKLQDQALKDSIPFAVIGSNTV

VEARGRRVRGRLYPWGIVEVENPAHCDFVKLRTMLVRTHMQDLKDVTRETHYENYRAQCI

QSMTRMVVKERNRNKLTRESGTDFPIPTVPPGADAETEKLIREKDEELSWTLWPSLGNPA

SCRETRLSETHGLRAGPRVAATILRLLVPNPGPVGLLILGGSSRQTRRLEQCFAHSKRLI

NAITLILLSPHFTDEQRLEQCFAHSKRLINAITLILLSPHFTDEQRLEQCFAHSKRLINA

ITLILLSPHFTDEQCLEQCFAHSKRLINAITLILLSPHFTDEQRLDQCFAHSKRLINAII

LILLSPRFTDEQCLEQCFARSKRLTNAIIIIKTSRLEAGRRSKPPGPRAPTLVGWFLSWG

ASGTPPRSRSARRRTLVVVVVVVVVVVVVACPLGAPLGNRVFIGVILEPVGGEEGPPSLE

YIQAKDLFPPKELVKEEESLQVPFAVLQGEGVEFLGRAADALIAISNYRLHVKFKDSVIN

VPLRMIDSVESRDMFQLHISCKDSKVVRCHFSTFKQCQEWLSRLSRATAQPAKPEDLFAF

AYHAWCLGLTEEDQHTHLCQPGEPVRCRQETELARMGFDLHNVWRVSHINSNYKLCPSYP

QKLLVPVWITDKELENVASFRSWKRIPVVVYRHLRNGAAIARCSQPEISWWGWRNADDEY

LVTSIAKACALDPGGKAAGGAACSGNGQGSEAGDTDFDSSLTACSGVESSSGPQKLLILD

ARSYTAAVANRAKGGGCECEEYYPNCEVVFMGMANIHSIRNSFQYLRAVCSQMPDPSNWL

SALESTKWLQHLSVMLKAAVLVSNAVDGEGRPVLVHCSDGWDRTPQIVALAKILLDPYYR

TLEGFQVLVESDWLDFGHKFGDRCGHQENAEDQNEQCPVFLQWLDSVHQLLKQFPCLFEF

NEAFLVKLVQHTYSCLYGTFLANNPCERELRNIYKRTCSVWALLRAGNKNFHNFLYVPGS

ELVLHPVCHVRALHLWTAVYLPASSPCTLREESVDIYLAPAAQSQEFSGRSLDRLPKTRS

VDDLLSACDTSSPLTRTSSDPNLNNHCQEARVDLEPWSGQPEGADPPAGDHGPVGPPRPT

PELGHLPPPPASRKDFRTDKTLTRHKSCPPSCKVPSPVALWPPESGSLDPQLKGGEEIPE

PAPELPARDTSLRTRDGPGEPPEEPSGKEAAGPPADAASSGQDGTGNLPEAPCQELAPDA

SGKASQAPVPGQKAPDPGSDGRRAARRAGCPALEDPLAAPPLRRDPSGPAGSGASCQGPP

NPGPDPGPREEDGGRRENGRNGPSAESSRFGKAPPELGRKPVSQSQMSEFSFLGSNWESF

QGMVASLPSGEPAPRRLLSYGCCGKRSGGKPTRPTGLCPAGQWAQREGVRSPICSSQSGG

HCAGPAGKSNRTWPLGRPKPASGPKPAPASCPSPGPPLYLDDDGLPFPPDVVQQRLRQIE

AGYKQEVELLRRQVRELQMRLDIRHRCAPPAEPPVDYEDDFTCLKESDGSDAEDFGSDPS

EDGLSEASWEPVDKKETEVTRWVPDHMASHCYNCDCEFWLAKRRHHCRNCGNVFCAGCCH

LKLPIPDQQLYDPVLVCNACYEHIQVSRARELMNQHLKKPIATASS

>ABHD11

MLRGARAWRLRPPRSLCLARASTHGRPSTCVYLYVIIMAFIKRLLCAKRCCKRWGGYKVI

RPVPLSFTQFDGPTQETPLVFLHGLFGSKTNFQSIAKSLVQQTGRKVLTVDARNHGESTH

SSEMSYEAMSADLQALLSQLGLPRCVLIGHSMGGKTAMTLALQKASGPGPRVWPELVERL

VSVDISPEETTGVSDFLSFVEAMQAIQIPKELTRSQARKLADERLKPVIQEVSVRQFLIT

NLVEVAGRYVWRVNLEALTHHMDALMGFPQLPGTYSGPTLFLGGSNSPFIRPSHHPKIRR

LFPQAQILSVAGAGHWVHADQPRDFIAAIRGFLT

>IL1B

MPAYGEADSSREVEETDNQKTTRRPGLESPMARVPDQSSDLMDCYSGDGEEQFYEDNGPS

QVKSGFQDLKARTCQEARACQEDDCSPCQMGIELKVTELSSSRGFRRAVVLVVAMEKLKR

QVVACDMSFMDRDLMDIFTTIFKEEPVSCTSWEQTLVTDSIYHYLRCQEVTIWDQEHKSF

TLNTMANPCELRALHLIGANATQEVKLNMNFYYKTEHPAGPTVKQPVTLGIKGGNPGSLY

LSCVKKGDKPTLQLEVVNQSDLEGKNQERFIFNKSTEGTSTTFESAAYPNWYISTSREED

EPVFLGASKGEEAITNFYLN

>IL18

MLMAGEAPQKINEDQSDIIFFQRGIVGFTDRYQFQPSSCPGSYLACEKKQNDPYFTMILKKV

SGDENDSTHMTLKPFPQ

>NEK7

MRINLKWKKSARCLDFCQQRSSAHAQERRRLTVEVIQGCGLVVRDRRRERGASVLSSGER

VVSRASLWSSREGSLGMEAKGGHDELRKLKGVKRSEVSVGKQHRFAERKCVGEKTGEKVV

KALRPDMGYNTLANFRIEKKIGRGQFSEVYRATCLLDGVPVALKKVQFNVQDRHSQLSMR

IESKAFEKVSLVHLAATLYVGRD

>LHX9

MVMRAREAVYHLSCFTCTTCTKTLTTGDHFGMKDNLVYCRAHFETLMQGDYPPQLSYTELAAKGGG

LALPYFNGAGPGTVQKGRPRKRKSPALGVDIVNYSSARFFAPVRALA

>GSDMD

MGVKTKTPKREKPDQVWEDAASTDKLVVATAGGEAGYRDWSSKEATAAATTVWLSGKSPG

FGVRGQKSSLCSRKLSQSLLSPLEGWQPSPDALLGRKGPISLFSPVFQLGVYHPAIPQAK

PARMFAQLTKKVAKKINAEGELLPLLSMNNSKRFRPLCLVRRKRKGTLFFGPRFRPTNLS

LLDVLDSDLPAPELKREDKFSLQDIVDGRFRAEVDLPDSLLSVKVSGEIKRVQKYSLEVQ

IVLISSEDLKRMQDERKLKKNEPEELKELRSLGENLYVVTEVVETLEEARLSSESKAEGS

CFLKLLSIHMKALCNHEEVVNIGKGCILAFRLGHLIFRDNWKILHTPTKEKTFPSEVLEK

GDPFVKDMAIAKDARGFEDLQREVNEEKQHLKYLDRQLKETILQAVQDLLGHREEMQKVE

DVLEDAMDGKGTQRLEGPGNIFLTTLEENSGQVMPELTGTVLYLLGALLVLSDTQQQLLK

LILEKGLLPQQLKLVKSILEQTFPMSQGGHFFLTLGPEDEEQSFTLALLEQYGLELPGPN

SSFLWKPDALASLSALYGALSLLDRRN

>ZC3H3

MRTARRPLWACAPPPARLLLMRRRRAPSPRQRGGPFAPEAGCRLMTSTAGGAQARAKAQA

QAQSRPPAMEEREQLRRQIRILQGLIDDYKNVHGNSRTPPAAGPRWPPPSYRGRGTFGVG

YSRPARRDFFPHQGPSWRKKYSLVNRPPGAPEQPEGGTPPSRDRPGPPPPDPRRQVQLRP

DQNMVIRIQAPSDAGSAGGSRARQDAPRSDPGLRKKDGEAGGSSREEGTSLVCRKEKGKP

RVVNSVGGGPGRPREPRWTASENARGLTRQALPARPQSSEGDAVGKAGSRIPDALCLQRL

RPGREPLLKNSLAPATKTTREPSLPGPCRTPKFRRTNYTWVASTVKAPRGPPRRPLSPRA

AAEGARKAPSSGAADRPVKPQPRADPGVKPRKPAAPSKPRGSSSKYRWKATGLTPAAAAP

FQWRAEAPGKSDGPPASPDGADFPAPGQASGGPGGWKPTFGESALSAYKVKSRTKIIKRR

GSVSLPVDKKSSLLPPATPKGHSSLRRKPSPRAKSSPASKRTPNRGAAQVTNHRLRRVPA

PRAHTPGKEAAALVGREPPAAAGTRSSPVPTCWRAVGPDAKGSTLDKPLDHQKEHCTKRL

TDLPVGPPDLRRSAVREIPQCSSESVCREPGILFYSGWIGGLRSGSVRETLWSGLRNREF

GGRRERRFGPGDGNLLETSEMPFH

>MROH6

MAARVGEGAQLELARAEGPGSPDPPPQPKAKPTRARRGRPKVTGHPRLVVAPGAQPPPCP

VGALTLAALAEEIQSHRGGQAGPSQQRAVQGDRQTAEPASRRPALEGNGGQHGAASAETG

EDTASRTRKKQQQQQPQQQPPQEEPRRESQPCSPAQSTLQPLASASPCTPGPEQFPLASC

FLTDLAVHTVACLTDAGFSGTQATAACLSSTLEAHGTILRDKVQELVHGLHLQIHRFSEG

RARRAALRVLCSLAVEHAQDVALRLPARTPGSFSSAVELWRGLSRNQRVNMMVLVQLLWK

LKGHPRLPGSSPAGSDGTLQEPLAATRALGEMLAVAGCVGAMRGFYPQMLIALVTQLHQL

ARCPPDSLSKACGHPQSKGGHPHGHAHCAVEALKALLRADGGRMVVTCMEQAGGWERLSG

PDTHLEGVLLLASAMVAHADHHLRGLFADLLPLLRSPDATRRLTAMAFFTGLLQSRPTVR

LLRAGAILERLGAWQGDPEPSVRWLGLLGLGHMALHAGKVRHVEILLPALLGALGEADGR

LVGAALGSLHRILLQPCGHSCTNSICLDVGARLWPLLDDGRDPVRCSAIGLFGTLVGRSP

VPQFCAIRDLVLDSLVPLLLHLQDQSPDAAEQSAEWTLARCDRFLHWGLLEEIVTMAHYD

SPEALSRTCQCLVQWYPGRVPGFLDQARGYLRSPQVPIRRAAGMFIGFLVHHTDAGTVRE

GLMDSLLHSLRELEWDPEASVRSTTHVTQHQLRLASQDWAARPGRFSPRRLLRPRGRPAR

PWPLYEEGPFKRRSRAGLWGSHMGA

>NAPRT

MGSRKPTCLPETVWEDKRKYDEAERRYYEREAAQAAPEASPPPEALNGLSQEESGDSGGQ

DPRMQKKRKRSPRHKTPGLDLALVGLSADHVWFDKPLFDRAERTFRAMLADGPAGEGPEA

EAAEPPPAGTPCDHGNRTACHHVVRGIWVNKFYFDRAERAFVERTQVSAPPRPLDLPALP

RPEAHQASGWGTPDEGYITAVPTPAAAGLPPAGEEGPTGSPPFALGWPGPSSPPANGKPQ

PAGLRAPTAEPRFASAERCFYQAAFDGHPPGKVRLQEREGWQDAARRGRKDRRNRNPPGK

RPKRAEPCGPKEADSPPPTCYFPREDSEPRWPGKPPLGGPGARHRAARTLRTARPALARK

APPAASAPGPLLDTRDPHSVSAIRPKKMATSFLMQEKIWFDKFKYDEAERKFYEQMNGPV

NSSSCPQENGASTILRDIARARENIQKSLAGLKTVLQSPPETPSQADAGASAPAGSSTGP

SGDHNELASRVASLEVENQSLRGGEKGPRETGPERGGWEGASSGAGVGSEAELLVGTEGS

PFCHTVIQDLQLAMSKLEARLSTLEKCSPSHRSPAPQTQHVSPMRKVEPSAPAPAPKAAT

PAEDDEDDEIDLFGSDDEEEDKEAARLREERLRQYAEKKSKKPGLIAKSSILLDVKPWDD

ETDMAKMEECVRSIQLDGLVWGGSKLVPVGYGIKKLQIQCVVEDDKVGTDILEEEITKFE

DYRGPDIGRGRSSRARGPGDARERNTTGSGPDQHRIKPGSGPDQDQTRTGSRQDQDRIRL

YQDRIRTGSGPDQDRIRPGSGPDQALPGQDQVRIRSGSDPDQDRIKTGSGADQDRIRPGP

GQNQARTRTGSRQDQTRTRTGSGPDQDRIRPGPGFTRTGSGPGPDQNRIRTGPGPEPDQD

QFRPRRSPDQDRTKPSPGPDQNQTKPRPGPDQDQRRPGRSPDQARVRTGRSPDQARIGRS

PDQDRTKPKPGPDQNQTKPRPGPDQDRTRPGRSPDQARIRTGRSPDQAQIRTGRGPGEAQ

TRPGSGPDEAQTRPGSGPDEAQTSPGSGPDEAQTRPGSGSDEAQTRPGSGPGEAQTRPRS

GPDEARAKPRPGPDQDRTRPRPGPDQDWTKPRPGPDQDRAKPRPGPDQDRTRPGRSPDQA

RIRTGRGPDQARIRTGRSPDQARIRTDEARAKPRPGPDQDRTRPGRSPDQARIRTGQSPD

QARIRTGRSPDQARTSPEPGLAQARARPIPVTMAYGYWRAGRAQEQAHFDLFFRRCPFGG

GFALAAGLRDCLRFLRRFRLRDPDIDYLASVLPPDTDPAFFDYLRGLDTSGVTVWALPEG

SVAFPMVPLLQVSGPLPVVQLLETTLLCLVNYASLVATNAARLRLIAGPEKRLLEMGLRR

AQGPDGGLSASIYSYLGGFDATSNVLAGQLCGIPVAGTLAHSFVTSFSGQEQLPTGALAP

GDLSAQAETWLTRVCEHLGRQVKDAHPGERAAFVAYALAFPRAFQGLLDSYSVMRSGLPN

FLAVALALADLGYRAIGVRLDSGDLIGQAQEIRRVFQNCAAHFQVPWLEFISIAVSNNVD

EALLAQLAQKGSEVNLIGIGTNVVTCPLQPSLGCVYKLVTVGGQPRLKLSEEEEKRTLPG

CKAAYRLGGPDGALLMDLLTLAEEPPPQAGQELRVWPLGSGQESRTLTPATVETLHRLYF

QRGQECESLPTLTEARALAQESLSRLSSAHKRREAPEPYQVALSEKLHALLESLCRSSRG

L

>SYK_like1

MDTYRAPSAVLDARRAQSSVLDAYRSAVLDTSQSSSVVFIERFLCAEHCTKRLEKYKLAT

YRDSPYPTTGSEHSAVLDTSYSVQSAVLGAYRAQGTVLSAYCRQRAVLGVYRVQRGVLDA

YCMQSAVLGVYCMQSAVLGLYPVQGTVLSAYHMQSAVLGVYCMQSAVLGPYPVQGTVLSA

YHMQSAVLGVYCMQSAVLDASHCIHSFIQSYLLGAYCSPSNPLMRLYKETRDALAHAKIV

PHAAGHPHQAAVNRAENAAELNPYVTQRGRRGPGDAQQEEPVWALPMDTAVYESPYADPE

ELRPDAVELDRGLLTLEDGELGAGNFGTVRKGFYRMKKGDKAVAVKILKDGGGGGGGVGS

GGVDEAVKEELLREADVMRRLDNPYIVRMIGLCRAEAWMLVMELADLGPLNKYLQKNRHV

QARNLTELVHQVCMGMRYLEEHSFVHRDLAARNVLLVTQHYAKISDFGLSKALGADQNYY

RSAVLGVYRVRSAVLGVYRVRSAVLDASYRVQSAVLGVYRVQSAVLDASYRGQSAVLGIY

CVQSAVLDASYHVQSAVLGVYRVQSAVLGTSYRVQSAVLGVYRVQSAVLGTSYRVQSAVL

GASCVQSAVLGVYRVQSSVLDASYRVQSAGLGVSWGQSAVLGVYCVRNTVPSIQCVQSSV

LGAHRRFVRPDATPLTAGGDAQTHGKWPVKWYAPECINYYTFSSKSDVWSFGVLMWEAFS

YGQKPYKGMKGSEVSAMLEKGERMQSPEGCPAEVYDLMNLCWTYKVEERPDFAAVELRLR

NYYYDISN

>Syk_like2

MCEPCRTGCVRAVCGPCGGHFVGRERAVSRRPCPSRVGALCEPCGDHVWALEGVMYEPWA

SRVTQAVGETCAGCVWAVREPCGGHFVGRERAVSHRLCASRVGALCEPCGDHVWAMGGVM

YEPCHAGHGGHFSGRERAVSRRPWASRVGALCEPCGGHVWATEGVMYEPCVSRVTQAMDE

TCAGRVRAVREPCGGHFVGRRVRAVWGPCANHVETICGPWKGSRVSHVTQAVDQTCAGRV

REPCGGHFVGRERAVSRRPCGGHFVGRGRAVWGPCTNHVEACVGQEWVMYEPCRTGCVRA

VCGPCGGHCVGRERAVSRRPCVSRVWALCEPCGDHVWALEGVMYEPCHTGHGRDMRRPCA

GRRVQAMCVPWFAGRGQSVGRPFCAPCESRAAQAVCMPRFAGHGRAMCVPRFAGRVRAMC

VPCFAGRVRAVSEPCAGHFVHHVQAVCRPWASRVQAVGESCAGHFAGRVKAMSCRPCAGY

VPATFCRPCAGYVRATFCRPCAGYVRAMICRPWAIRVHFARRVKAMLLRPCAARVQAMCM

PCFAGHVRARLVQCLASSKRFTNAIIIIIRLVQCLAPSKRFTNAIVIIIIIIIITIINLS

NGDEAQTPGAMASSGGDGWGHLPFFFGNITREEAEAHLEEAGLGEGLFLLRQSRSSLGGF

SLSVSSGGRVHHYTIERDVSGAFAISGGRSHPGPAELCAFHGREADGLVCRLGDPCVRPP

GLRPRAGPFEGLRETLIRDYVRTTWNLQVGGRGREAEIAGDGFRGSGGASIQGQALEQAI

LSQRPQLEKLIATTAHEKMDWFHGALTRSQAEDALLAAPRAEGKFLVRSREPAGSFALCL

LHGGRPLHYRIDKDKAGKLSIPDGKKFDTLWQWAHSLELSLRIKISIIEIVIDDNDDDDK

IIIIRININNDDDDDDDSPYPTWAHSLELSLRIKISIIEIVIDNNDDDDKMICIKINVNN

DDDDDSPYPAWAHSLELSLRIEISIIEIIIDNNDDDNKIIIKINVNNDDDDDDDDDDDSP

YPTWAHSLELSLRIKISIIEIVIDNNDDDNKIIIIKINIDDDDDDDDSPYPAWAHSLELS

LRIKISIIEIVIDDNDDDNKIIRIKIHVNNDDDDDDDSPYPTLVEHYSYKADGLLRALDS

ACPRKHNGSDVLGSRPPLPGDHPRAGGLIGRLHSFQRTKKSTVLSAWEVQVGNIERQSLP

NNGLGAQRCAGRLLLCAERCAGRRVQGTVLSAYRRQRAVLGVYRVQSGVLDAYCMQSTVL

SANCVQSAVLGPYPVQGTVLSAYRMQSAVLGVYRVQSAVLDAYCMQSAVLGPYPVQGTVL

SAYHMQSAVLGVYCMQSAVLDASHCIHSFIQPYLLGTYCVRSTVLSAWKSTNWQHIETVP

TQQWAQSTALYWTPPNHHQSYLLSAYCVQSTVLSAWEVQIGTLCRTLCWAPTVCRVLCWA

PINQSIVFIERFLCAEHCTKRQGKSKLAAYRDSPYPTVGSQSRSLSCAKRCAGRLLCAER

CAERLLFIKGAGAGRRSAGRQQGAPGVARMLRTAHARIPSSAPLPSPSNPLMRLYKETRD

ALAHAKIVPHAAGHPHQAAVNRAESAAELNPYVTQRGRRGPGDAQQEEPVWALPMDTAVY

ESPYADPEELRPDAVELDRGLLTLEDGELGAGNFGTVRKGFYRMKNAGAVVLICCRLGLP

ERSVQCSARRKRSINTIDDDDDDDDDDDDDDDGEGRLEQCLAHSKRLTNIIIIIIITTII

NLSNGDEDLSAERGRPGGRVRSREPAGSFALCLLHGGRPLHYRIDKDKAGKLSIPDGKKF

DTLWQWAHSLELSLRIKISIIEIVIDDNDDDNEITCIKIKVNNDDDDDDDDDSPYPAWAH

SLELSLRIKISIIEIVIDDNDDDNEITCIKINVNNDDDDDDDRPYPAWAHSLELSLRIKI

SIIKIVIDNDDDDNKIICIKINVNNDDDDDDDDDDDSPYPAWAHSLELSLRIKISIMKIV

IDDDDDNEIICIKINVNNDDDDDDDDSPYPTWAHSLELSLRIKISIIEIVIDDNDDDDKI

ICIKIHVNNDDDDDDDDSPYPALVEHYSYKADGLLRALDSACPRKHNGQHPPPPPPVTSP

LHPTRPVTSSGTEPAPPAHPDTWPLGAANPDCPRG

>AUH

MTSDSKARGLSIKRLLRAKHRAKRQGGVPGPPPDGRPPPRPPMEALKPVPQRDAALPPPP

SPSPSTSSRRRRREFTPEEKKDAQYWEKRRRNNEAAKRSREKRRLNDLVLESRLLALGRE

NAALRAELLALKARFGLLPPPPPSPSSAVKTEPPEPGAGPEADEAGGGKTPSDGEDEQRV

PKGPAALPHKLRLKFRGAPAAAPAEGPPPFALQSAVGPSVRARVGHGGDGVREALATRRR

RPVDRIHRSTSIHPAPASPGPGRSVDRIYQSINQSVDQYLPSAGFSRSQSVGRSDLINQS

VLIERWLLPVPIAVFLERLRCARRYTERLGESSTAADTFIELRPWVAEEKRLPGRARGRW

RRPRCRCRAGGWGGVWRGARFRFRWGPGAVRGWGSEPRDEDELSLRFLPDEDKGIAVLGL

NRPQAKNALSWNLIKQLSHSLDALKSDKKVRTVIVRSLVPGVFCAGADLKERAKMEAGQV

GAFVAKVRGVVHELVQCSAHGKRSINTIDDDDDLDFPKRLVQCSARGKRSINTIDDDDDD

DLDFPKRLVQCSAHGKRSINTIDDDDDDDLDFPRRLRLVQCSAHGKRSINTIDDDDDDLD

FPRRLVQCSARGKRSINTIDDDDDDDDLDFPSAYAWNGAGHIRLERCWAHSKRLINAIII

IIAMMIFSRVTLGESLPSSGPQFPHLLNGDEDLIPPYPPQRLERCRAHTRLPVPTIAALD

GLALGGGLELALACDIRVAAASAKMGLVETKLAVIPGAGGTQRLPRTVGPALAKELIFSG

RLLDGAEARAAGLVTHSPPQNGQGDAAYRRALALAREFLPQGPVAVRAAKLAINQGMEVD

LVTGLAIEEACYAQRQSSSSSIVFIERFLCAERCTERLGSPSRQHLETVIIIIIINRIY

>NFIL3

MTSDSKARGLSIKRLLRAKHRAKRQGGVPGPPPDGRPPPRPPMEALKPVPQRDAALPPPP

SPSPSTSSRRRRREFTPEEKKDAQYWEKRRRNNEAAKRSREKRRLNDLVLESRLLALGRE

NAALRAELLALKARFGLLPPPPPSPSSAVKTEPPEPGAGPEADEAGGGKTPSDGEDEQRV

PKGPAALPHKLRLKFRGAPAAAPAEGPPPFALQSAVGPSVRARVGHGGDGVREALATRRR

RPVDRIHRSTSIHPAPASPGPGRSVDRIYQSINQSVDQYLPSAGFSRSQSVGRSDLINQS

VLIERWLLPVPIAVFLERLRCARRYTERLGESSTAADTFIELRPWVAEEKRHREPAVG

>ROR2

MEEKGVEKEEKGVEEEEEQKEEGGRRKRRGEEEDERGEEEEEEERRMKKGGGGGDGEKGG

EKEEEEEEEQKEKGKREGEEDGFCQPYRGIACARFIGNQTIFVRSLQMQGDIENRVTAAL

TMIGTSTQLSDECSRFAIPSFCHFVFPLCEPGGGARPEGPTAPARPEGPEAPAAPARPRP

LCRDECEALESDLCRQEFGIARSNPLLLMRLELPRCRDLPPPGTPDAQRCVRLGVPPPPL

PTRGAHFTSPPGGARHTPPTPHHQGALTLPHHQGAPSTPNPHLTTRGRSLHLTA

>DIRAS2

MPEQSNDYRVVVFGAAGVGKSSLVLRFVRGTFRETYIPTIEDTYRQVISCDKNICTLQIT

DTTGSHQFPAMQRLSISKGHAFILVYSVTSKQSLEELQPIYEQICQIKGDVHKIPIMLVG

NKSDESQRELAAGEGEALAARWNCSFMETSAKMNYNVQELFQELLNLEKRRAVCLQVDGK

KAKQQKKKDKLKGKCSVM

>ZAP70_like

MDDSRIPKQLLYSELNWGGRKPGELTECFKDIVSSCGPEPPKDPKETPDRTAGEELRITS

WPMPCPGLVNPGPGGTAKVSPSPEATATQSAWPQCGPLAPAPLWEGASRGLGLIPGGCII

SASPGATAATVTGPAFRSPFPTPNGELGSATEMPDAAAHLPFFYGSISRAEAEEYLKLAG

MADGLFLLRQCLRSLGGYVLSLVHDLRFHHYPIERQLNGTYAIAGGKAHCGPAELCEFYS

RDADGLCCTLRKPCNRPSGLEPQAGVFDSLRETMVRDYVRQTWKLEGEALEQAIISQAPQ

VEKLIATTAHERMPWYHNAISRDEAERMLFSGSQPDGKFLLRPRKEQGTYALSLIYGKTV

YHYLITQDKAGKYCIPEGTKFDTLWQLVEYLKLKADGLIYCLKESCPNASVPTGTAAPTL

PAHPSMPRRIDTLNSDGYTPEPESCQVGLGRAAGGCSRKKAEAEPCGSGRLVAGGDAKAG

EKSRILPMDTSVYESPYSDPEELKDKKLFLKRENLMMDEVELGSGNFGCVRKGVYKMRKK

QIDVAIKVLKSGNEKAEKEEMMKEAQIMHQLDNPYIVRIIGVCRAEALMLVMEMALGGPL

HKFLSSKKEEIPVSNVVELMHQVSMGMKYLEEKNFVHRDLAARNVLLVNQHYAKISDFGL

SKALGADDSYYTARSAGKWPLKWYAPECINFRKFSSRSDVWSYGITMWEAFTYGQKPYKK

MKGPEVISFVEQGKRLERPTDCPPEMYTLMNDCWIYKWEDRPDFSMVETRIRTYYYSIAS

KADLATSPVQGAEAACA

>ADAMTS10

MAIACRLLSWALAFSLSLPSQPASAFQSQEEFLSSLKSYEITFPVRVDHNGAFLDFAPPQ

RQRRSLGTRPPEPAEPRVFYKVEALHTRFLLNLTLTSHLLADHFSVEYWKRDGLDWRHHI

RQECLYAGHLQGQRLSSKVAISNCHGLHGLIVADEEEYFIEPLNGRGSGVPEGEGSPHVV

YKRSSLQRPHLDAACGVLAPAMVGHPSQSFGLLADEKPWKGRPWWLRPLKTAPTKPLGNQ

TQRGQLALKRSVSQERYVETLVVADRMMVAYHGRRDVEQYVLAIMNIVAKLFQDSSLGNI

VNILVTRLILLTEDQPTLEINHHAGKSLDSFCKWQKSIVNRNGHGNAIPENGIANHDTAV

LITRYDICIYKNKPCGTLGLAPVGGMCERERSCSINEDIGLATAFTIAHEIGHTFGMNHD

GVGNGCGARGHETAKLMAAHITMKTNPFVWSSCSRDYITSFLEHRLFPRNSRALTHASEK

QRGSVERARALESEAVGSNPGSATCQLCDFGSGLGLCLNNAPPKQDFVYPTMAPGQAYDA

DEQCRFQYGVKSRQCKYGEVCSELWCLSKSNRCITNSIPAAEGTICQSSTIDKGWCYKRV

CVPFGSRPEGVDGAWGLWAPWAECSRTCGGGVSSSTRHCDSPRPTIGGKYCLGERKRYRS

CNTDDCPPGSQDFRELQCSEFDSVPFRGKYYTWRTYRGGGVKSCSLNCLAEGFNFYTERA

AAVVDGTPCRPDTIDICVNGECKHVGCDRILGSDLREDKCRVCGGDGSSCETIEGVFAPT

LTEGGYEEVIWIPKGSVHISIRNLNLSLSHLALKGENDAFLLEGKPGPSPQLRLPLAGTI

FHLRRGPDQPECLEALGPTNATLIVMVLVRSELQGIRYRFNAPITHEALPPTYTWHYAPW

TKCSALCAGGSQVQAAECRKQPDGSPVPLHHCKAHAKLPERQRSCNTEPCPPSWAVGNWS

GCSRSCNMGARTRSVVCQRRMSPNEEKTLDDSACAQPRPHVLEPCSSQSCPPEWAALDWS

ECNPSCGPGLRHRVVLCKSGDHSATLPTSQCSAATKPPTSMRCNLRRCPPPRWVAGEWGE

CSAQCGFGQQLRSVQCTTHTGQPSGDCTAALQPPATQQCESKCESSPTESPEECRDVNKV

AYCPLVLKFKFCSRSYFRQMCCKTCLGR

>MYO1F

MGSKERFHWQSHNVKQSGVDDMVLLPRVSEEAIVENLKKRFLDDYIFASSNEGWGREGWS

EFPQTDSHWLNVKATQEKVNQLGGGVHTGGERDSPEIVALDPAAQTYIGSVLISVNPFKQ

MPYFTDREIELYQGAAQYENPPHIYALTDNMYRNMLIDGENQCVIISGESGAGKTVAAKY

IMGYISKVSGGGDKVQHVKDIILQSNPLLEAFGNAKTVRNNNSSRFGKYFEIQFSRGGEP

DGGKISNFLLEKSRVVTQNESERGFHIYYQLIEGASQDQRQNLGIMTPDYYYYLNQSETY

KVDDTDDRSDFHETLNAMQVIGIPTEVQQLVLQIVAGILHLGNISFCEQGNYAQVESADL

LAFPAYLLGVDSGRLNEKLTSRKMDSKWGGRSESIDVTLNVEQAAYSRDALAKGLYARLF

DFLVEAINRAMQKPHQEYSIGVLDIYGFEIFQRNGFEQFCINFVNEKLQQIFIELTLKAE

QEEYVQEGIKWTPIEYFNNKVVCDLIENKLNPPGIMSVLDDVCATMHATGGGADQTLLQK

LQAAVGCHEHFNSWSSGFVIHHYAGKVSYDINGFCERNRDVLFSDIIELMQSSEHAFIRM

LFPEKLDADKKGRPTTAGSKIKRQANELVSTLMKCTPHYIRCIKPNETKRPRDWEESRVK

HQVEYLGLKENIRVRRAGFAYRRPFQKFLQRYAILTPETWPHWRGDERQGVQHLLHSVHM

EPDQYQMGRTKVFVKNPESLFLLEEMRERKFDGFARTIQKAWRRHVAVRKYEQMREEASN

ILLNKKERRRNSLNRNFVGDYLGLEERPELRRFLGKRERVDFADSVTKYDRRFKSIKRDL

ILTPKRLYVIGREKVKKGPEKGQVQEVLKKHLDIQVLRSVSLSTRQDDFFILHEEAADIL

LESIFKTELLSLLCKRFEEVTQRALPLTFNDTLQFRVKKEGWGGGGSRNVNFSRGSGEMA

TLKISGKTLMVSIGDGLPKSSKPTKKGAPRSQSRGRRPAPARSAPGPPRGTCRNGAPPAM

APGSSQQRLEQMYAGHQKQSRGPPAAMLPKQGASRRMRARPPSEQNLEFLNVPDQGMAGM

QRKRSIGPRPPPGVGRPKPQPRAPGPRCRALYQYVGQDVDELSFNVNEVIDILLEDEVIE

AQRSENGAVEDGADVSAAPRRRGSRIRTKAPGGGEGRSGMDQQEEKPSPGSGGQAGPAPV

PFPDLYTSSSRNQGPKLEKDQLAPTFPLGDCSSPCLSGSGRTNVVPSTSSSRHTQDLRLQ

KRRPLPGKQYPCSSYGCKLVCSSSQELAHHLRSHYLPTQSMGGKLFHCSTLGCADTFPSM

QELVTHMKVHYKPNRYFKCENCLLRFRTHRSLFKHLHVCSDPSRSPTAGPPPPALEKEPP

EPEPSAGPSPEAAPLLSPLPLGPSHTQPFPLLEPSLFDPASLPRFPAQASSPMPGAFLPY

LPPSPYSLPPGSGQQRLRPFLPAQALPISNAIWKKSQGVSGSPRRPPGGSEVLAVFLKLQ

SCRRRHYCCFCSWETGAQQGLACQVLCGRRKQGHSSNSRIVWEHTRGRYTCMQCPFSTAS

RPAMTLHLEDHRKTPPPPARLDAHMDFGVGLAAFPSKLPAEMESSLYSQL

>TMEM131

MAAFIQSENIMEVLRFDDGGLLQTDAPIGLGSYQQKSVSLYRGNCRPIRFEPPMLDFHEQ

PVGMPKMEKVYLHNPSSEETITLISISATTSHFHASFFQNRKIPPGGNTSFDVVFLARVV

GNVENTLFINTSNHGIFTYQVVEMFSSGGDLHLELPTGQQSGTSKLWVSERQSSSPGEGG

DQKPGVESGQRKMRGSGMPAPWGRRAPLPVWGKTDNFCPGVFWIAVIIRCLSRYSVGAQY

SVRPQEIPPYETKGVMRASFSSREADNHTAFIRIKTNASDSTEFIILPVEVEVTTEMLDF

GTLRSQGKVLYIDTLGKFLGPLLFSIYTHSLGEFIRSHVFNYHLYADDTQIYISAPALSP

SLQARISSCLQDISIWMSARHLKLNMSKTELLIFPPKPCPLPDFPITVDGTTILPISQAC

NLGVILDSALSFTPHIQSVTKTCRSHLRNIAKILPFLSVQTTTLLVQSLILSRLDYCIRL

LSDLPSSCLSPLQSTLHAAARIVFVQKRSGHVTPLLKNLQWLPFNLRIRQKLLTLSFKAL

HHLASSYLSFSSPARTLCSSATNLLTVPRSCLSRRRPPAHILPLAWSALPLHIRQASSLP

AFKALLRAHLLQEAFPD

>CLEC_like1

MDNEVTYADLKFQDSFKPQRIQEFDNSREIEHPAPSPAWRWSALGLLTLCLLLLIGLASL

GILYHACKPCPETWFWHEKSCYGASIVKQTWEDSREACAAVNSSLVKIDNKEEWDFIASL

QNRQYHWVGLYQNPNGRQWEWEDGSALSRDLNSLVSGDRTGGKMCAYTYGSYFYIDPCTD

KHYYICEKVAGLVKKLIAG

>CLEC_like2

MPREHLESAIEVALTPFSQIQALDAQRQIELLFQAEFLFPAFLLWPTANPWATQTSLKAA

DFEEVPGAFLSFGLEEPEATTGSWSSMGVSHNQWGLLGYPGYRKRANRAGGLQSLLMFQL

TSPDSPGSGRSPCGQGSGGLKGGGGEEEMANEVTYANLKFQDSSMAQRIQKFDTIQEIAE

AMSFSERDINRPLGFIEKQRSSVERARALESECLEKCFARSKRLINAIIIIIKGRTLIET

ITGIPTKKPWATDFLTAACEFHCGLPGTARGAAVIRPFLWTRVFQEKKNIQQLNGVKENL

SLQLDISANISKEKDLIQSDLSDALKKMATKLCRELTRNKQAPDCLSNLVSEHACKPCPE

KWQWHRDSCYWIARKLNLEKSRKVCAENNSSLVKIDNKEELVFVASKLNPYYWVGLSRNI

SSGQWVWEDGSTLSPDLQTGRTCFRAGLQALPGEVVFTQGQLCEDFEGKQTRDESRDSCA

TQNSSLLNIDNKKEWMEQRAALAIHATTIGERHPTLGYRSLLQGYQVFWGEAPASSDLPV

HFHVWRLLSLLLLTLCVLQLIGVVGFGIRYSILFRGICRDCFKQKEFLLSQNSNLSADLR

EVTTKLCQELIMKQPDHACRPCPEGWHWHGYNCFKILMDKRTWYESREACAFQNSSLMKI

DNKEEWNLLTPKIQSYHWVGLSRNASDLSLKWEDGSEINPKVLLLLSDAKTKGRLCAVVY

RNDLSLDFCNNTYPFICEKAAEPVKKELLT

>CLEC_like3

MKEEVTYADLSFQRTDDVEKVPQMDLIEKPVSSLPQLGSSSKGSRGNWPIFRCFGVWAGG

LKPGGLDLGNTAYSEFCLSVLDRTICSPVCEEICSPGSADTVFAATAAAVAAAERTRGPR

DPLCFNGFLFPESKCSPCEKGWQWSGDSCYRKFDIWNTWPKGKKFCHDNNSTLVKVDSRE

ELLIGLVVLGIKFSQARSQEIGGTSSPNDKPENLDINGSCSKQQEFLLSQNSKLSAGLRK

MAIELCREVTRNKPGLRREEESANLVDVQKKGVPGQGEDVGWWSTAGQVRTKHSEEFSSR

GAEGAGWAVEGKKGESKCSPCEKGWQWSGDSCYRKFDIWNTWPKGKKFCHDNNSTLVKVD

SREELLIGLVVLGIKFSQARSQEIGGTSSPNDKPENLDINGSCSKQQEFLLSQNSKLSAG

LRKMAIELCREVTRNKPESKCSPCGKGWQWSGDSCYRKFDIWTTWPKGKKICHDNNSTLV

KVDSREELVRSISMELTGLKAENVEDEGGYTMMSIYRQTSIRGLAGSEPARSKASMEDED

GYTMLNLKSSRIHDFSKGLSGHKCNPCSSSKYHQGNCYHLYLRNRTWEENRIYCASKNYT

LVKVDNQEELAYLTRSTHKIRWIGLSRTANDAPWTWEDGSVPAVDLFQLSGDEEAKHCAL

FHNGKIEAAGCQENYPSLCESVGGNIKIDLLL

>CLEC_like4

MQDEDGYIMLDFKSRIHATSKGPSESGCPGLDRHGNLPALKGRGLSADPPGLFPSWCWMT

LALLILCLGMLIGLIALGSMWLSTVLCTHLTKGPRKGGVVEGKESSNKRSDQLYDVRGMV

DIHSPASTNTDSRQQALLQQITQKYCQELSSKPGGHKCSSCDNNWRFHGGKCYGTFKNNK

TWEESKKYCDDRNSTLLKIDTQEAWNFIQGKPDFTRWIGLSRPSSGGRWMWMDNSALTDN

LFELSGDGDEGKHCAYIQKKQISTTFCRELHYYICEKLPPRPSADSAILPEPPAPLIILE

LRLSGQSHYLEVMQDQGAYDSLCWVTPDPPPVKRPSLRTEHQDTQQQEQPLQQQQQQQLM

CLSLPRRPDACSTTRTNTLRQGPRDRNLVAVSYGDVLALLPAFAAFHCHPEPQVLLRCHV

FMSKIELFVFPPKPCPLPDFPISVDGTTILPVSQARNLGVILDSARSFTPHIQAITKTCW

SQLRNIAKIHPFLSIQTATLLVQALILSHLDYCISLLSDLPSSCLSPLQSILHAAAQIVF

VQKRSGHVTPLLKNLQWLPINLRIRQKLLTLGFKAVHHLAPSYLTSLLSFSSPARTLHSS

AANLLTVPRSRLSRRQPPAHVIPLAWNALPPHICQASSLSPFKALLRAHLLQEAFPD

>CLEC_like5

MAYVQSDQLVSTSALSTVPGNVIQASELIGKQEEILANLSHQQHVCSESLQMCQVRIRMF

TSPESNCSPCLEPWVKNGKSCYLFFDQWKNWTSSSEFCLQEKSELLKIGSKEELNFINQN

IEKKKMGSSWSYWVGLMQDGCFGDWRWRDSTVPSSDLWPKQGSWSAGETCGHLTHGVLSS

ASCSKWKYLICEKCTSSAVDFQLD

>CLEC_like6

MRRWRKRHCYCFTSYETETVPDLVILYLGQCFAYRHVAPTRTWRPVALTLLILCFVLLFG

LGALGFEFFQVFRLSNTQRTAISQQEERLGNLSQQLQDLQAQNRKLTGTLQVAQKLCWEL

YNKTGARNLGVILDSALSFTPHIQAVTETSRSQLRNIAKIRPFLSIQTATLLVQALILSR

LDYCISLLSDLPSSCLSPLQSILHATARIVFVQKRSGHVTPLLKNLQWLPINLRIRQKLL

TLGFKAVHHLAPSYLTSLLSSSSPASTLPPPPPSSATNFLTVPHSRLSRRRPPAHVVPLA

WNALPLHIRQASSLPPFKALLRAHLLQEAFPD

>CLEC_like7

MQDEDGYTILPPYVRLNPCHPAASDKGQSCLDSRMEDEDGYTILNPRTRAFARDPAASDK

GLPAVSPRWRPAAVTLGIVCLGLLGATGILASPQTTTPRDLEQQQREPASWQLGEETTGV

GVKDFVFLRIPNSCRSITKQSYDSVDTSLPERPASICNHSGRVFSVKIQKWSFVAFFHTV

NLSLCLRLTPTLLLLPRTASHLPPFVPDDHAKQLQGISSVAQWKEPGLWSQRGLNAGESH

AVGNGNSQRLAEGLSPPQRRRPRECRPSPPLRAGGGQRWRRSSLGNQSVVQMRAKHSSTQ

DMLEDDGDTTLSLHSRTSTAAGSPKPAGPDCEPAVG

>CLEC_like8

MGIKSVSPMWDNLITLYPPQRLEQCFAHMCISAGVSVWACAYLQDMEDSVQYAELKFKMP

EEKPKQKLPEEKAKDSSSLSPWWFPAAIVLGIFSFGLLGAVAVLGLEINQAHGLMNQQVK

NYTHLEGETAQFTDQKEVAKATPRDQEHVEDLLGKLENITEERNALLLQNEHLQEALKKV

KNYTGPCPQDWLWHTESCYSFPSRSTNWKESQENCASQGAQLLKVDNQDELEFIYQATVH

SRNPFWLGLRRSEAISRWLWADGSAPFVGLLQAWRYISHTYPSGTCAYIFQDNIFAENCI

ITAFSICERKANLLKLQ

>CLEC_like9

MASEIVYAEVKFKMDTQKLGAPPAPAPQKATPPPLRPWLPGLLMALLLLLLLLLLLSFLV

AFVVFYRRSHLCREDRSSKSTRILSEFVCAKQSSKTPVFSCSYIETHQENEDPPSPHQFP

KNQTTLDCITEGSEVEGRSWDCCSIGWRPFRSRCYFISTDKMPWAESQQNCSQMGAHLVV

ISSDTEQKFLESILDNHDVYFLGLTDLEGSKDWRWVDQTPYNKSVLFWHPGEPNYSWERC

ASLHWINYRGWGWNNIRCNEKQNRISRQWSKSPAASRPVECPPLSCSIVIPSDLLGIEVT

NGQRGVLQPAAE

>CLEC_like10

MVQTVCKHSVNVIDWLYERNSQAQTDRGGQCPRGAHIGLTDIFERRDSQTMVQSTFASPT

LPASTIIVTGISILLLTSCFTASCLVTQNGFAQLCKDSNGLAPLNHTQLSCLRERPQVEG

RMWSCCPQDWIPFQTSCYLFPTDVLPWDEAQSKCLKQQAHLVVINTEAEKNFITQGKPFN

FSIHLGLKKWKMESQWRWEDKTPYNPADTFGLEGDLAEGDGARRCAGLTLGQPSIRWRWS

STDCAAAAHRICEMPRRSF

>CLEC_like11

MVSILLLSACFIARCLVTQRAFSRFCKEEGTLLRLNDIFTEISCYSSGSGSIQDCCPRGW

KHFQSHCYFFSSDTLTWSSSLLNCTGMGAQLVVINSLEEQEFIFHSKPSGREFYLGLTDQ

QVEGKWTWVDGTAYDPSWSFWDLGEPNNILGLEDCATTRDSPSAKETWNDVTCFSFHYRI

CEMPAQTLSAGKKGM

>NECAP

MAAEAEYESILCVKPDISVYRIPPRASNRAYRNFPSPSPPPGERYPPPSGDRAPILIIHC

WVGTVSICCQLVLPKRLVQCSAHKHCTTRLGEYNITDAFPDHSQLSGKNYGPGNPLLTHT

ALMASDWKLDQPDWTGRLRITSKGKVAYIKLEDKVSGELFAQAPVDQYPGIAVETVTDSS

RYFVIRIQDGTGRSAFIGIGFSDRGDAFDFNVSLQDHFKWVKQESEISREAEKPDTHPKL

DLGFKEGETIKLSIGNITTKKGGPTKPRPVGAGGLSLLPPPPGGKISIPPPASSVAICNH

VTPPPVQTSSQGGGESDILLDLDAPAPSTKASASVPAAPDLWGGFSTAARMLTNKFLFSA

HSAASDTGSVPGGPCKSPHPQKHYSQKLPVPLIPLCSVFKSMSPR

>C3AR1

MGTDCEPHVGQPDHVVSPQRLEQCFAHNKRLTNAIIIIIIMGTCRGHRMGRMSVPWGNTS

SPDSPLQQSHSLPELLSMVILSFTLVLGLLGNGLVMWVAGWKMQRTVTTVWFLHLTAADL

LCCLSLPFSLAHLALGGHWPHGQLLCKLVPATIVLNMFASVFLLTVISLDRCLLVTRPVW

CQNHRGVRLATAVCGAAWVLAFVLCLPILLYRETYVDGPLWRCGYNFGHHASLDGLEDTG

LMDIGLVGFSDSSPPTPEMDDLGTLDVPLRARDALLTGSDWLLGSSLPWDPLDTKAGHPS

SIPNPDLSPRGDLLPTEMPSQPPGELDLDTDFWDDLIKIYAAESQVPGPLVAMTLTRLGL

GFLVPLAIMVACYSVTVVRVQSSRFAVGQTRTFWLATRVVAAFFTCWAPYHLVGVLSLLA

TPDSPLDHAVAALDPLTQALASANSCINPLLYALAARDFRVRARLSLRSILEAAFSEGLS

HSSTCPPSQAVALSIDPLGTEV

>CARD9

MATNCTVLFQVLNTVLCTQRSGAGHGQTQTASPSQALSGGGPGRMMGSERGGGFFLESCH

PNCPLLPPRAFWRHLTDWRSAGDDVEAKKTEVKGRLGVSRSGSEVIAAKKRNGGAPGGRG

PERDRRSEEPSEMFGPRFLPYRGVGEPVSWLRLRVVVIVFVTTPTRGDPNGSPGGRDGAL

APCCKLCIWLPSRKTYYQGTTLQASISSPVASGRMIPGILKPRHGDKMQRHLSVAGIGSA

PTTGIFQARIVLREASSGCHLKRSPSREVHWAPAMSDYENDEECWNTLEGFRVKLISTID

PSRITPYLRQCKVINPDDEEQVLNDPSLVIRKRKVGVLLDILQRTGHKGYVAFLESLELY

YPHLYKKITGKEPTRVFSMIIDASGESGLTQLLMNEVLKLQKKVQELNLLLNSKEDFIKE

TRVKNSMLRKHQERAEKMKEECQAFSQELKKCKDENYNLAMSFAKQSEEKNAALMKNRDL

QLEIAQLKHNLMKAEDDCKVERKHTTKLKHAIEQRPSQEVMWEMQQEKDLLLAKIQELEN

SIREGKREKNSLYITVLEEDWRQSLMEHQEKETTIFHLRKDLRQAEALRNKCMEEKEMFE

LQCTTLRKDSKMYKDRIEAILQQMEEVAIERDQAIMTREQFHLQYSQSLIEKDGHRKQIR

ELGEKCDELQLQLFTREGQLLSMESKLKRLQLETPNLSSDLEETSPRNSQELTLPRSLDE

DAQLSDKSEPAILPSLAIIEKSPGEGSDL

>MALT1

MPTLQIPHGNSSELIFNPIQVNDAGFYVCRVNNDTTFEFSQWAQLDVCELMESSYGNLGGFSESKLQI

CIEPKPQKLIPGDTLLLHCVAVGSPIPHYQWFRNGFPITDETKKLYMVPYVDMEHQGTYWCHVYNDQ

DSQNSKKVEVIIDELNNYGQNAKDKVALLIGNMSYLNHPKLKAPLVDVYELTNLLRQLDFKVVSLLDLT

EHEMRNAVDEFLLLLDKGVYGLLYYAGHGYENYGNSFMVPIDAPNPYRSANCLCVQSILKLMQEKET

GLNVFLLDMCRKRNDYDDTIPILDALKVTANIVFGYATCQGAEAFEIQHSGLANGIFMKFLKDRLLEDK

KITVLLDGVAEDMGKCHLTKGKQALEIRSSLSEKRALTDPIQQTVYSAESLVRNLQWAKAHELPESMY

LEFKCGVQIQLGFAAEFSNVMIIYTRIIHKPAEIMMCEAYVTDLPLEHLIFTVHLSYNYQGMEDTVDERQ

EVNVGKPLIAKLDIYRGFGRKSCFQTSLMSSPSNLAPISGAAEHYHSFPNSFPGAFDPHPGNSGCDLP

SISCHCSRTSDMVFSSQSTQDYSSQFCKSNIPVETTDELPFTFSDKLKFSEK

>BCL10

MPRTIVWTGVDRRKSDQTLSPSHMWPTVQGNPVPLTPPGFATCKVPLQTFHLGEFNRGQQQSRKS

QVRGLNYSSCLSSPYGATNNLSRSHSDESNSSVNVNDRDSTVIYHPEGESSTATFFSTVPSLNLPIVE

EGSTENAVFSSATLPGPGEPGAPPLPADLQREDEESSGNSSDNLFLPLSPGQVVPERSVSREPFRR

PAEEMESAGRGVIDVTRRSLVSRGNPFLKLKSAPPKKSFLVSVIFPVMGLATRPWCRVLNFFLEA

>MYD88

MAAAAPGPSPGPSPGPSIDSPPGSSPGSSLGSSPGSPPGPSPSLALELVPLVALNFKVRTRLALYLN

PQTPVAADWTALAEELGYEYLQIRHFQAQPDPTGRLLEDWERRGRQATVGRLLALLAKLQRDDVLS

DLGPSIEEDCRKFLLKQQEEAEKPVQVAAVDSSVPRTAELSGITTRDDPLGQMPELFDAFICYCPSDI

QFVHEMIRQLEQTDFGLKLCVSDRDVLPGTCVWSITSELIEKRCRRMVVVISDDYLQSNECDFQTKFA

LSLCPGARQKRLIPVKYKAMKRDFPSILRFITICDYTNPCTKAWFWPRLAKALALP

>TNFA

MRRTGPSADQTLPPPPPSSTDPAGGTVRHPSTAQAMSMENMLRDIELAGEPAGKTAGARRFGHCL

CLSLISFFLVAGATTLFCLIHFGVIGPQDREQDSPLLPLTQMLKSYQPSAEKPAAHVVANSQEDKKLV

WVGGRANALLEGSVILNNNQLVVPASGLYLVYSQILFKDSSCPANPDDSPILTHNVSRFSESYEHEVSI

LSAIKTPCQGGAKGTWYEPIYQGGIFRLNQGDRLSSQTNSPEYLDFSMEGQVYFGIIAL

>PANX1

MTSDSQTQATSPVVLVQTREGPSIFLNDNKFFPYILLLVAILLYLPSLFWRFTAAPHLCSDMKFIMEELD

KVYNRAIKAAKSFRDIDVRDAAGSVLALHDNGGQSGWEIPENHFKYPIVEQYLKTKKRSKNLIVKYLLC

RVLTFLIILLACVYLGYYISLSSLSDEFVCSIKSGILRNDSTVPEQFQCKLVAVGVFRLLSFLNLVVYVAL

APVVVYTLFVPYRQKTDVLKVYEILPTFDVLRFKSERYDDLSLYHLFLEENISELKSYKCLKVLENIKGS

GPGVDPMILLTNLGTVKTDVVDGRESKSGEEAEPQPAATGPGELRVLTDQDLSNETKANNEEVKIRQ

RLLDSS

>P2X7

MPGDCSWRNVCEYETDKVVRIQSVTYGNIKWILHMIVFSYVSFALVKDKLYQQKEPLISSVHTKVKGL

AEVKGHRPPKIFDTADYTFSLPGNSFFVLTNFIETYQRQGECPEYPSPRTLCSTDRSCKAGRMDPQS

KVLSNLENISVGKYSGLRECVSKYWAMLDEAHIIFSSLRPALLKSAENFTVLIKNNIDFPAHNYTTRNIL

PDLNTSCTFHKIQNPQCPIFRLGDILRETGEEFSEVAIKGGIMGIEISWDCNLDRWFHHCRPQYSFRRL

DDKTTNESLYPGFNFRYAKYYKEGGIDTRTLIKAYGIRFDILVFGTATVFIDFLISSYSNECCRTRIYPCC

NCCKPCVVNEHYYRKKCELVVEPTSTLKYVSFVDEPHIRMVDKQLLGESLQDAKGENIPELVLSRPTL

QFMLFYRDPLMTLDPDGLTRELRHCAYKRYIDWRFGSEDMVDFAILPSCCRWRIRKEFPKPGGQYS

GYKSLH
