# Supplementary material for: Conservation of NLRP3 Inflammasome Pathway in Monotremes and Large-Scale Restructuring of the Caspase-1 Gene Cluster Region in Mammals
Source: J Mol Evol. 2026 Mar 12;94(2):353–68. doi: 10.1007/s00239-026-10307-6 (PMC13076529; doi:10.1007/s00239-026-10307-6)
Supplement: Supplementary file 3 — Supplementary Material 3 (Online Resources 9-21) [file 239_2026_10307_MOESM3_ESM.docx]

**Conservation of NLRP3 Inflammasome Pathway in Monotremes and Large-Scale Restructuring of the Caspase-1 Gene Cluster Region in Mammals**

David Stevens, Tasman Daish and Frank Grützner

School of Biological Sciences, Adelaide University, Adelaide, 5005 SA, Australia

Address correspondence and reprint requests to Prof. Frank Grützner, School of Biological Sciences, Adelaide University, Adelaide, 5005 SA, Australia, email: frank.grutzner@adelaide.edu.au

Journal of Molecular Evolution

**Evolution of the Dectin family in mammalian species**

As described in the main text the Dectin-1 cluster consists of *Dectin-1* (*Clec7a*), *Clec1* (*Clec1a*), *Clec2* (*Clec1b*), *DNGR1* (*Clec9a*), *Micl* (*Clec12a*), *Mah* (*Clec12b*) and *Lox1* (*Olr1*) (Online Resource 9a) while the Dectin-2 cluster consists of *Dectin-2* (*Clec6a*), *DCIR* (*Clec4a*), *DCAR* (*Clec4b1*), *BDCA-2* (*Clec4c*), *Mincle* (*Clec4e*) and *Dectin-3* (*Clec4d*) (Online Resource 9b).

Clec2/Clec1b has a role in platelet activation in response to different stimuli (Alshehri et al. 2015; Christou et al. 2008). Clec1/Clec1a is able to negatively regulate inflammatory responses through interaction with Histidine-rich glycoprotein (Gao et al. 2020) while also providing protection against fungal threats through the recognition of 1,8-dihydroxynaphthalene (DHN)-melanin (Stappers et al. 2018). Opossum, Tasmanian devil and koala have *Clec1a* and *Clec1b* homologues present in their assemblies (Online Resource 9a and Online Resource 10). However, they lack any other *Dectin-1* cluster genes. The platypus contains a duplication of *Clec1b* (*Clec-like4* and *Clec-like5*). Echidna has at least one copy of *Clec1b* (*Clec-like4*). While the NJ tree suggests *Clec-like3* is also *Clec1b* the ML tree disagrees, placing it instead with *Clec12b* (Online Resource 10). Based on chromosomal arrangement, BLAST and phylogenetic data we predict echidna *Clec-like3* to be a *Clec1b* homologue (Online Resource 9a). The ML tree (Online Resource 10a) predicts that monotremes lack any *Clec1a* homologues while the NJ tree suggests that echidna *Clec-like6* is a homologue (Online Resource 10b). Based on these data as well as chromosomal arrangement and BLAST we predict echidna *Clec-like6* to be *Clec1a* (Online Resource 9a).

Both monotremes possess a copy of *Olr1* (*Clec-like8*) with very strong bootstrap support in both trees (Online Resource 10). Olr1 has been shown to detect GroEL on *Escherichia coli* and C reactive protein (Shih et al. 2009; Zhu et al. 2013). The lack of a homologue in the marsupial species examined could be compensated through other receptors such as TLR2 and TLR4, which have been shown to induce the NLRP3 inflammasome in response to GroEL (Zhang et al. 2024).

Clec12a acts as a negative regulator in response to monosodium urate crystals to regulate the inflammatory response to non-infectious cell death (Neumann et al. 2014). The NJ tree predicts a single homologue in platypus (*Clec-like3*) (Online Resource 10b). However, the ML tree result is much less clear (Online Resource 10a). Several monotreme Clec-like genes form a cluster prior to *Clec12a* and *Clec1a*. The bootstrap support for this is very low and this clustering may be impacted by the echidna *Clec-like6* and *Clec-like7* which exhibit long branch lengths suggesting significant divergence. Based on the phylogenetic results and BLAST we identify platypus *Clec-like3* as *Clec12a* (Online Resource 9a). The absence of this gene in marsupials suggest this regulatory response is absent or altered in these species.

Clec12b positively regulates the production of IFNγ-induced chemokines in melanocytes suggesting it plays an important role in skin immunity (Blot et al. 2025). The ML tree (Online Resource 10a) predicts the echidna possesses a single copy of *Clec12b* (*Clec-like3*). The NJ tree (Online Resource 10b) suggests that both platypus and echidna *Clec-like1* and *Clec-like2* are *Clec12b*. The bootstrap support is very low in both cases. Based on the phylogenetic data, and BLAST results we predict the echidna *Clec-like1* *and Clec-like2* to be homologues of *Clec12b* (Online Resource 9a). There is no clear consensus with our current data on platypus *Clec-like1* and *Clec-like2* and so we can’t confidently identify these genes. The lack of a *Clec12b* homologue in the examined marsupials suggests the IFNγ pathways may be less regulated in marsupial melanocytes.

Echidna contains a single copy of *Clec9a* (*Clec-like5*) (Online Resource 10). Clec9a has been shown to prime CD8+ T cells when it detects F-actin exposed by necrotic cells (Ahrens et al. 2012). No homologue was identified in platypus, the marsupials and dog. These data suggest the gene was present prior to monotreme divergence but has been lost in several species.

While phylogenetic analysis confirms that the platypus and echidna Clec-like9 are homologous it is difficult to determine the identity. In the NJ tree (Online Resource 10b) they form a clade predicted to have diverged prior to *Clec6a*, *Clec4c*, *Clec4b* and *Clec4a* while the ML tree (Online Resource 10a) predicts they diverged prior to *Clec4a* and *Clec4b*. *Clec4c* was only identified in chimpanzee and human while *Clec4b* was only identified in mouse and rat. Based on these data as well as chromosomal arrangement and BLAST we predict platypus and echidna *Clec-like9* to be *Clec4a* (Online Resource 9b). *Clec4a* is a negative regulator of immune responses (Kaifu et al. 2021; Richard et al. 2006; Zhao et al. 2015) and as such its absence in the marsupials suggests these species have lost a layer of regulation present in the eutherian mammals examined.


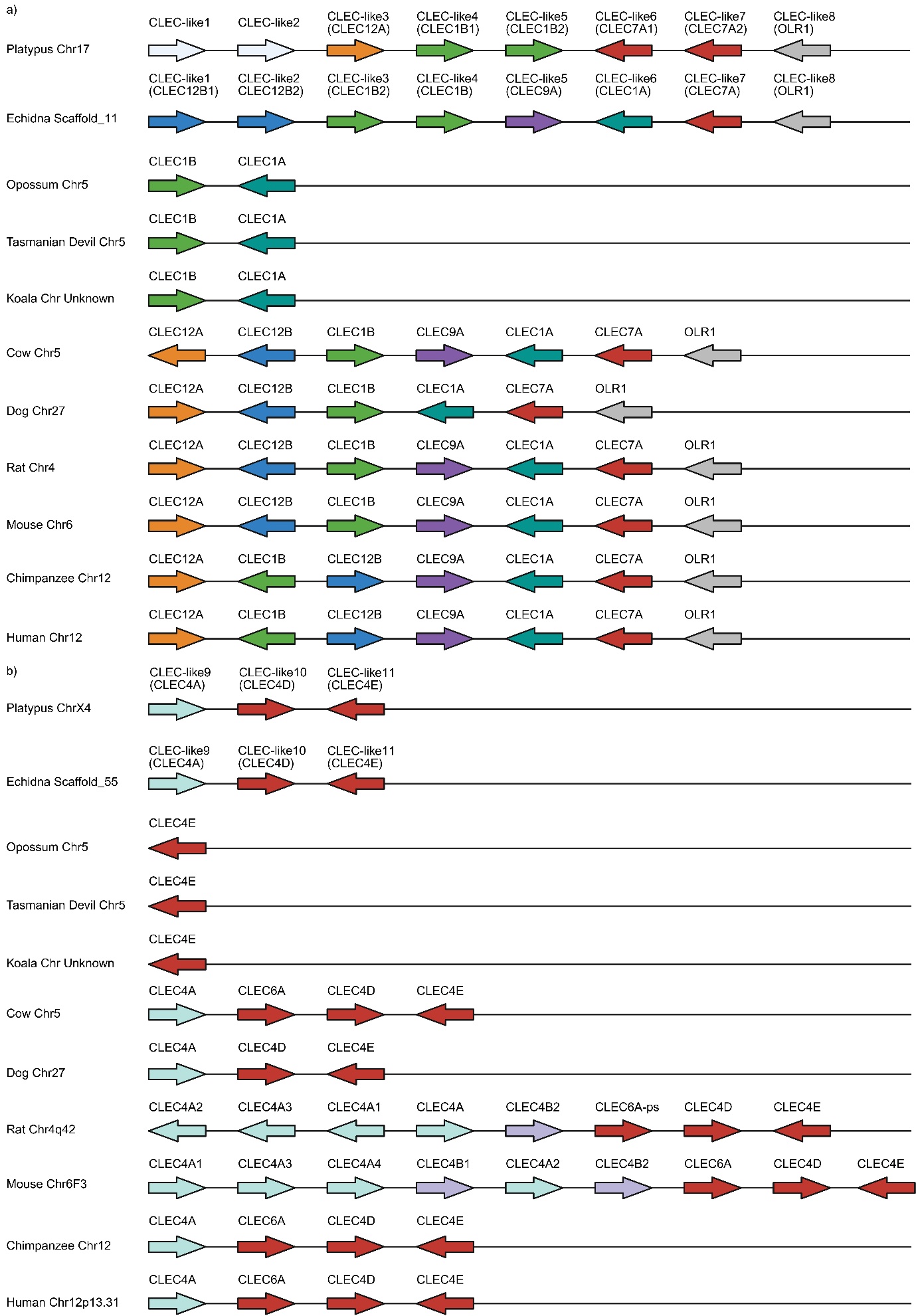


Online Resource 9 Chromosomal arrangements of the a) Dectin-1 and b) Dectin-2 clusters in multiple mammalian species. The monotreme gene identities (Brackets) are predicted based on phylogenetics, synteny and BLAST data. Red arrows represent the Dectin members with confirmed roles in the NLRP3 inflammasome pathway (*Clec7a*, *Clec4d*, *Clec4e* and *Clec6a*). The arrows represent the orientation of the genes. -ps at the end of a gene name represents a pseudo gene. Distances not to scale


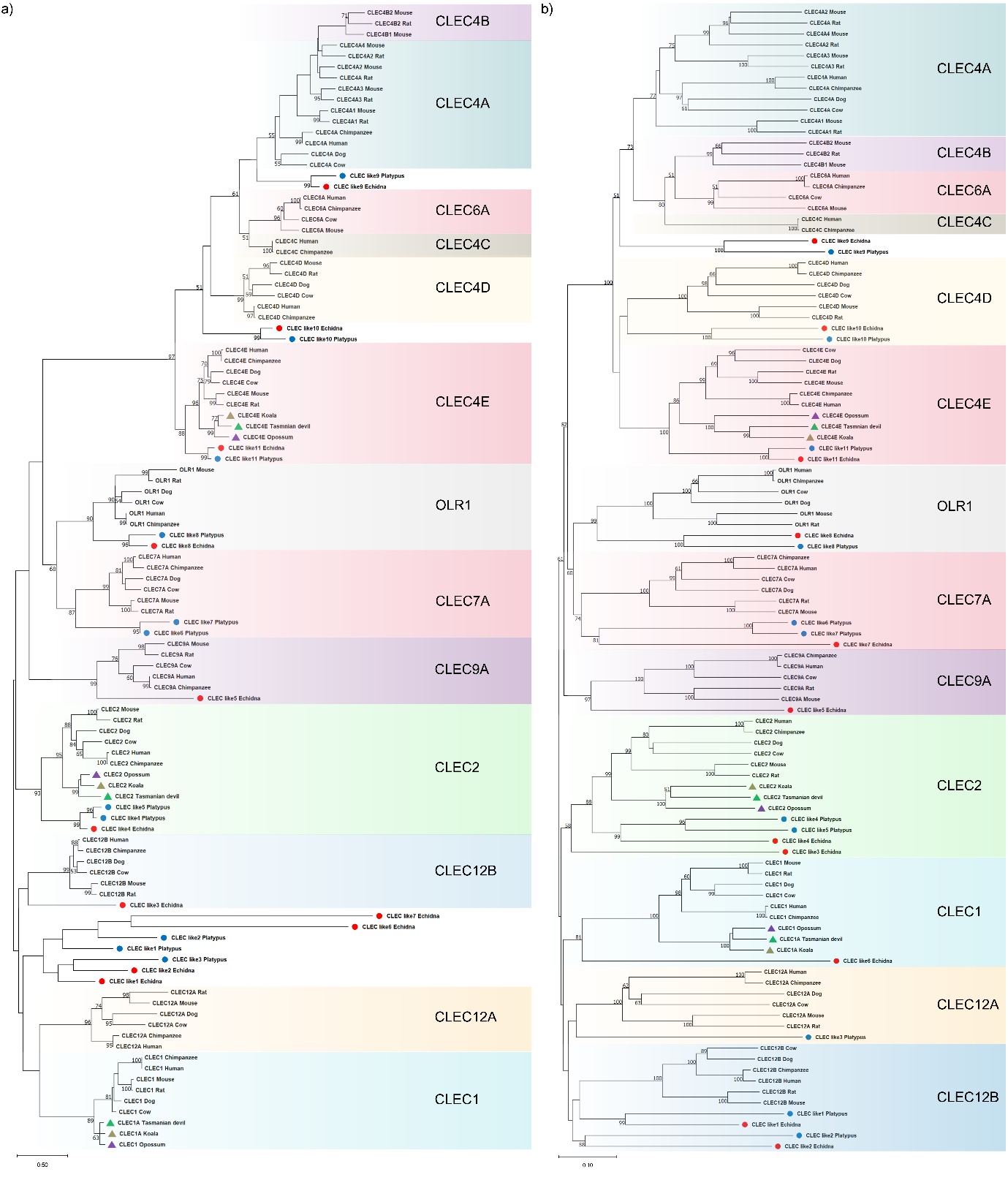


Online Resource 10 Phylogenetic analysis of Dectin family genes. Predicted protein sequences from multiple mammalian species were aligned in MEGAX using MUSCLE. a) The Maximum likelihood tree was generated using the Jones-Taylor-Thornton model with 1000 bootstrap replicates. Gamma distribution was used to model the evolutionary rate differences among sites. The tree is drawn to scale and represents the tree with the highest log likelihood (-19,689.47). 95% partial deletion was used to remove all positions with lower coverage. A midpoint root was used. Bootstrap values lower than 50% are not shown. b) The Neighbour-joining phylogenetic tree was generated with 1000 bootstrap replicates. The tree is drawn to scale. The p-distance method was used to compute evolutionary distances representing the number of amino acid substitutions per site. Pairwise deletion was used to remove all ambiguous positions for each sequence pair. The optimal tree with the sum of branch length = 19.665 is shown. A midpoint root was used. Bootstrap values lower than 50 are not shown. Monotreme sequences are marked by red (echidna) or blue (platypus) circles. Marsupial sequences are marked by purple (opossum), green (Tasmanian devil) or brown (koala) triangles

**Loss of Zap70 in platypus**

*Syk* and *Zap70* belong to the SYK/ZAP70 family and share domain structures. The echidna *Zap70* shows synteny with marsupial and chicken *Zap70* (Online Resource 11). The eutherian *Zap70* share synteny suggesting the gene has relocated following marsupial divergence. The echidna Zap70 is predicted to have two SH2 domains and a protein kinase domain indicating the whole gene is present and conserved in echidna. No *Zap70* homologue was found in the platypus genome assembly while the flanking genes *Myo1f* and *Adamts10* were found on chrX1 and *Tmem131* on chr18. No *Actl9* homologue was identified in monotremes. A *Nfil3-like* gene with similarity to *Nfilz* was found on platypus chrX2 however no *Zap70* homologue was identified in this region. Based on these data, *Zap70* is absent in the platypus. This is curious as homologues of the gene are present in many non-mammalian species and it has been shown to have a significant role in T cell development and in the regulation of T cell receptor responses (Ashouri et al. 2022; Negishi et al. 1995). Studies in *Zap70*-/- mice have shown it plays an essential role in CD4 and CD8 selection in the thymus (Negishi et al. 1995). In mice and humans, the loss of *Zap70* leads to immunodeficiency (Au-Yeung et al. 2018). It is possible another gene is compensating for this absence.


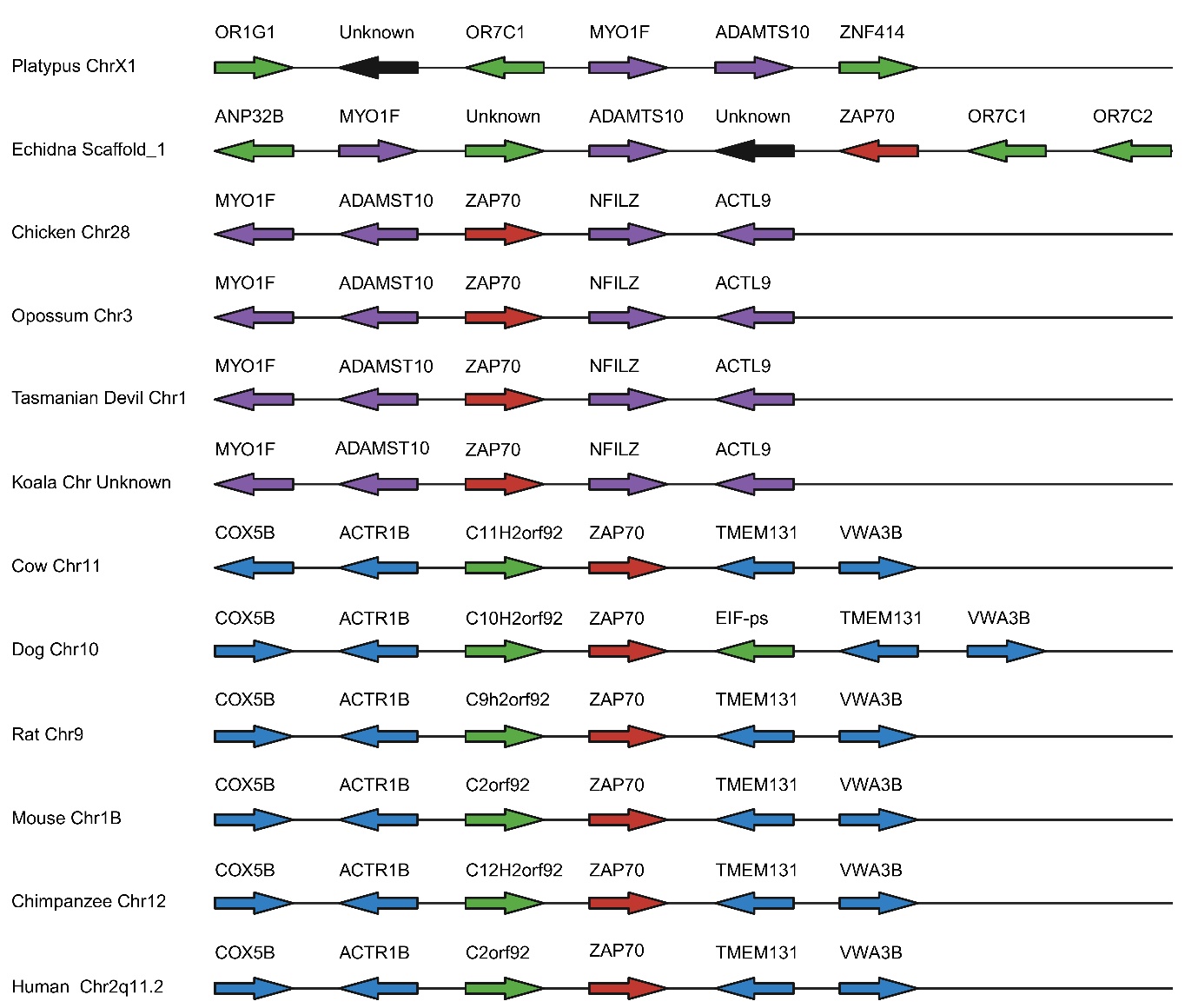


Online Resource 11 Chromosomal arrangements of *Zap70* for multiple mammalian species with chicken as non-mammalian outgroup. Arrows indicate orientation. Red arrows are genes of interest, blue and purple are conserved between species, and green arrows are species specific. The genes shown as unknown (black arrows) represent genes identified by GENSCAN that lack any apparent homologue in human. -ps at the end of a gene name indicates a pseudogene. Distances not to scale

**Evolution of the NLRP family in monotremes and marsupials**

The NLRP family consists of 14 members with functions involved in inflammation and reproduction with species specific duplications of *Nlrp4* in mouse, and *Nlrp9* in mouse and rat (Tian et al. 2009). NLRP12 is capable of negatively regulating the inflammatory response. It achieves this through the binding of IRAK1 to inhibit its activation (Zaki et al. 2011), as well as NF-κB and ERK (Zaki et al. 2011). A single copy of *Nlrp12* was identified in platypus (*Nlrp-like4*) and echidna (*Nlrp-like3*) (Online Resource 12a and Online Resource 17). These genes are in proximity to monotreme *Nlrp3* suggesting the gene may be the result of a duplication of *Nlrp3* prior to the divergence of monotremes. A *Nlrp12* homologue was also identified in opossum (*LOC100018825*), koala (*LOC110220084*) and Tasmanian devil (*LOC100923423*) (Online Resource 12b). Synteny is shared within lineages but not between them suggesting the genes have moved location through mammalian evolution.

Interestingly a gene identified by the NCBI automated systems as *Nlrp14* was found in proximity to the marsupial *Nlrp12* (Online Resource 12b). The phylogenetic results don’t support this identification and place the genes as either diverging before the reproductive NLRPs (Online Resource 17a) or grouping with *Nlrp5* (Online Resource 17b). One of the flanking genes, *Epn1* is located on human chr19q13.42 and shares synteny with *Nlrp9*, *Nlrp11*, *Nlrp4* and *Nlrp13* in human, one of the reproductive NLRP clusters in human. It is found on chimpanzee chr20 and shares synteny with *Nlrp9*. *Epn1* is found in proximity to *Nlrp2* and *Nlrp4c* in mouse (chr7), *Nlrp9* and *Nlrp4* in rat (chr1q12), *Nlrp9* in cow (chr18), and *Nlrp9*, *Nlrp13*, *Nlrp8* and *Nlrp5* in dog (chr1). While the identity of the marsupial genes cannot be conclusively identified we hypothesise that these represent a reproductive NLRP gene and at present we identify it as an *rNlrp-like* gene. The absence of a monotreme homologue would suggest the reproductive NLRPs arose after the divergence of monotremes supporting previous conclusions (Duenez-Guzman and Haig 2014).

The NLRP6 inflammasome is induced in response to viral dsRNA (Wang et al. 2015) as well as gram-negative bacteria surface molecules such as LPS (Shen et al. 2019) and lipoteichoic acid (Hara et al. 2018) and other DAMPs (Levy et al. 2015). Two *Nlrp6* orthologues were identified in platypus (*Nlrp6* and *Nlrp-like6/Nlrp6b*) and one in echidna (*Nlrp-like4*) (Online Resource 13 and Online Resource 17). No homologues were identified in the three marsupial species. The platypus *Nlrp6* shares synteny with the eutherian *Nlrp6* however no *Nlrp6* homologue was observed in this region in echidna or marsupials. Platypus *Nlrp6b* shares synteny with the echidna *Nlrp-like4/Nlrp6*. A search for *Mbd1* and *Comt*, two genes found in proximity, shows these genes to be present in therian species however they share no synteny. No NLRP genes were found in these regions supporting the initial results that opossum, koala and Tasmanian devil have lost *Nlrp6*. Based on these results we hypothesise that a single copy of *Nlrp6* arose prior to monotreme divergence with the synteny preserved through mammalian lineages. A lineage specific duplication occurred in the monotremes with the echidna losing the original copy of *Nlrp6* following its divergence. The marsupials appear to have lost *Nlrp6* although whether this was a single event in the marsupial ancestor or it has happened several times is unclear.

Mitochondrial damage induces the NLRP10 inflammasome (Prochnicki et al. 2023). NLRP10 has been shown to directly bind p63 and delay its degradation, promoting survival of keratinocytes and to reinforce skin barrier function (Cho et al. 2024). Two copies of *Nlrp10* were identified in platypus (*Nlrp-like2/ Nlrp10* and *Nlrp-like3/Nlrp10b*) (Online Resource 17). The NJ tree (Online Resource 17b) suggests that *Nlrp-like3* is a duplication while the ML tree suggests it is a *Nlrp3* duplication (*Nlrp3b*) (Online Resource 14). The lack of a LRR in both of these predicted proteins would suggest they are likely NLRP10, which is the only known NLRP to lack the LRR (Wang et al. 2004). The genes are in proximity to *Eif3f*, a gene found flanking *Nlrp10* in eutherians, however the other genes in this region are different. The syntenic region in echidna contains no *Nlrp10* homologue. *Nlrp10* was found in the three marsupials and shares no synteny with the eutherian or platypus genes. *Csmd2* and *Hmgb4* share synteny with the marsupial *Nlrp10* and these were searched for in echidna. While both genes are present in the echidna, they share no synteny and no NLRP gene was identified in these regions. Based on these results we conclude that echidna lacks a *Nlrp10* homologue.

Potential homologues to *Nlrp1* were identified in opossum and koala (Online Resource 15). The koala NLRP1 contains the PYD-NACHT-FIIND-CARD domains consistent with NLRP1 while the opossum NLRP1 appears to lack PYD. These genes share synteny with each other but not with eutherian *Nlrp1*. While they cluster with eutherian NLRP1 in the NJ tree (Online Resource 17b), they don’t in the ML tree (Online Resource 17a). Instead, the opossum and koala NLRP1 cluster with a group of NLRP sequences we are terming Nlrp-like as they have no apparent homologues among the known NLRP. The Nlrp-like cluster is present in both trees (Online Resource 17). Within this group are platypus and echidna *Nlrp-like1* and several marsupial *Nlrp3*-like and *Nlrp12*-like genes (Online Resource 16). These Nlrp-like genes lack the signature FIIND of NLRP1 suggesting these are not *Nlrp1* homologues.

Furthermore, while the monotreme *Nlrp-like1* appear to be homologous they don’t share synteny. The platypus *Nlrp-like1* syntenic region is present in echidna and contains a truncated gene which shows similarity to a NLRP, but the predicted protein lacks any recognisable domains. The echidna *Nlrp-like1* syntenic region in platypus does not contain any NLRP genes. The Tasmanian devil NLRPs in this clade appear to be the result of multiple duplication events and the genes are found in proximity to each other. The koala genes appear to be split into two clusters each containing a pseudogene. Koala *LOC110195196* and *LOC110195240* share synteny with the Tasmanian devil cluster on chr3 (*LOC100917067*, *LOC105749910*, *LOC105749909*, *LOC105750187*, *LOC100923605*) and opossum *LOC100018508* on chr4. Koala *LOC110195030* has no synteny with the other genes in the cluster. Due to the lack of synteny or domain similarity it is not possible to determine the identity of this cluster. However, our results suggest that opossum and koala contain *Nlrp1*, while monotremes and Tasmanian devil lack a homologue indicating a species-specific loss in the Tasmanian devil. This suggests *Nlrp1* evolved prior to the divergence of marsupials. The loss of NLRP1 has also been observed in Felidae (Digby et al. 2021). While there is some overlap between NLRP3 and NLRP1 function (Gorfu et al. 2014; Suzuki et al. 2014), the NLRP1 inflammasome also responds to unique threats such anthrax lethal toxin (Boyden and Dietrich 2006; Levinsohn et al. 2012) suggesting this response may be absent in these species.

Based on these data it appears that following mammalian divergence there were several duplications that gave rise to an increased repertoire of NLRP genes. From the platypus genome we can observe *Nlrp3*, *Nlrp6*, *Nlrp10* and *Nlrp12*. *Nlrp1* then appears following monotreme divergence. Our data supports previous results (Duenez-Guzman and Haig 2014) that suggest the reproductive NLRPs evolved after monotreme divergence with the presence of *rNlrp-like* in the marsupials examined. Following the divergence of the marsupials there is the expansion of the reproductive NLRPs as shown in both our data and previous work (Duenez-Guzman and Haig 2014; Tian et al. 2009). Our platypus and opossum results differ from those previously reported with the identification of a *Nlrp6* duplicate in platypus and *Nlrp3* in opossum (Duenez-Guzman and Haig 2014). These differences are likely the result of improvements to the opossum assembly and the new platypus assembly. Species-specific loss appears common in this family as multiple instances have been reported in the reproductive NLRP (Duenez-Guzman and Haig 2014). We observed species-specific losses in echidna which lacks a *Nlrp10* homologue and the Tasmanian devil which lacks a *Nlrp1* homologue. Opossum, Tasmanian devil and koala all lack a *Nlrp6* homologue suggesting this may have been lost in a common ancestor. The *Nlrp-like* cluster for now is a mystery. It’s unclear whether these are a novel NLRP that was lost in eutherians or whether these are very divergent homologues.





Online Resource 12 Chromosomal arrangements of *Nlrp12* and *Nlrp14* for multiple mammalian species. a) Monotreme *Nlrp12* shares synteny with *Nlrp3* b) Marsupial *Nlrp12* and *rNlrp-like* share synteny c) Eutherian *Nlrp12* and d) Eutherian *Nlrp14.* The predicted identities in brackets are based on phylogenetic results. Arrows indicate orientation. Red arrows are genes of interest, blue, purple, pink, light grey and dark grey are conserved between species, green arrows are species specific and black arrows are putative genes with no apparent orthologue. The yellow arrow represents a Nlrp-like pseudogene. -ps at the end of a gene name indicates a pseudogene. Distances not to scale


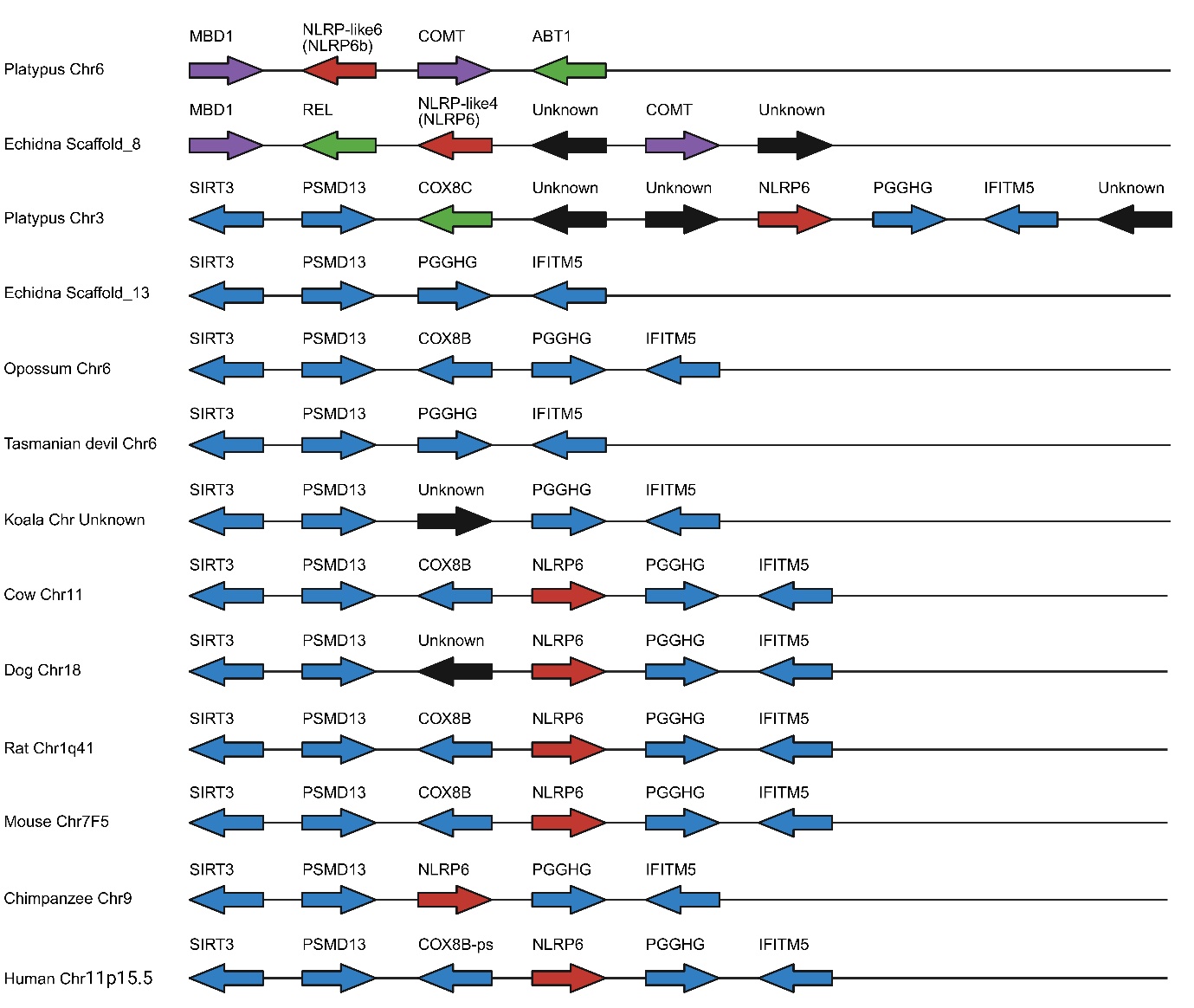


Online Resource 13 Chromosomal arrangements of *Nlrp6* for multiple mammalian species. Platypus has two copies of *Nlrp6*. The first is located on chr3 and shares synteny with eutherian *Nlrp6*. Echidna lacks a *Nlrp6* homologue in this region. The second platypus copy is found on chr2 and shares synteny with the echidna *Nlrp6* homologue. Opossum, Tasmanian devil and koala lack *Nlrp6*. The predicted identities in brackets are based on phylogenetic results. Arrows indicate orientation. Red arrows are genes of interest, blue and purple arrows are conserved between species, green arrows are species specific and black arrows are putative genes with no apparent orthologue. Distances not to scale


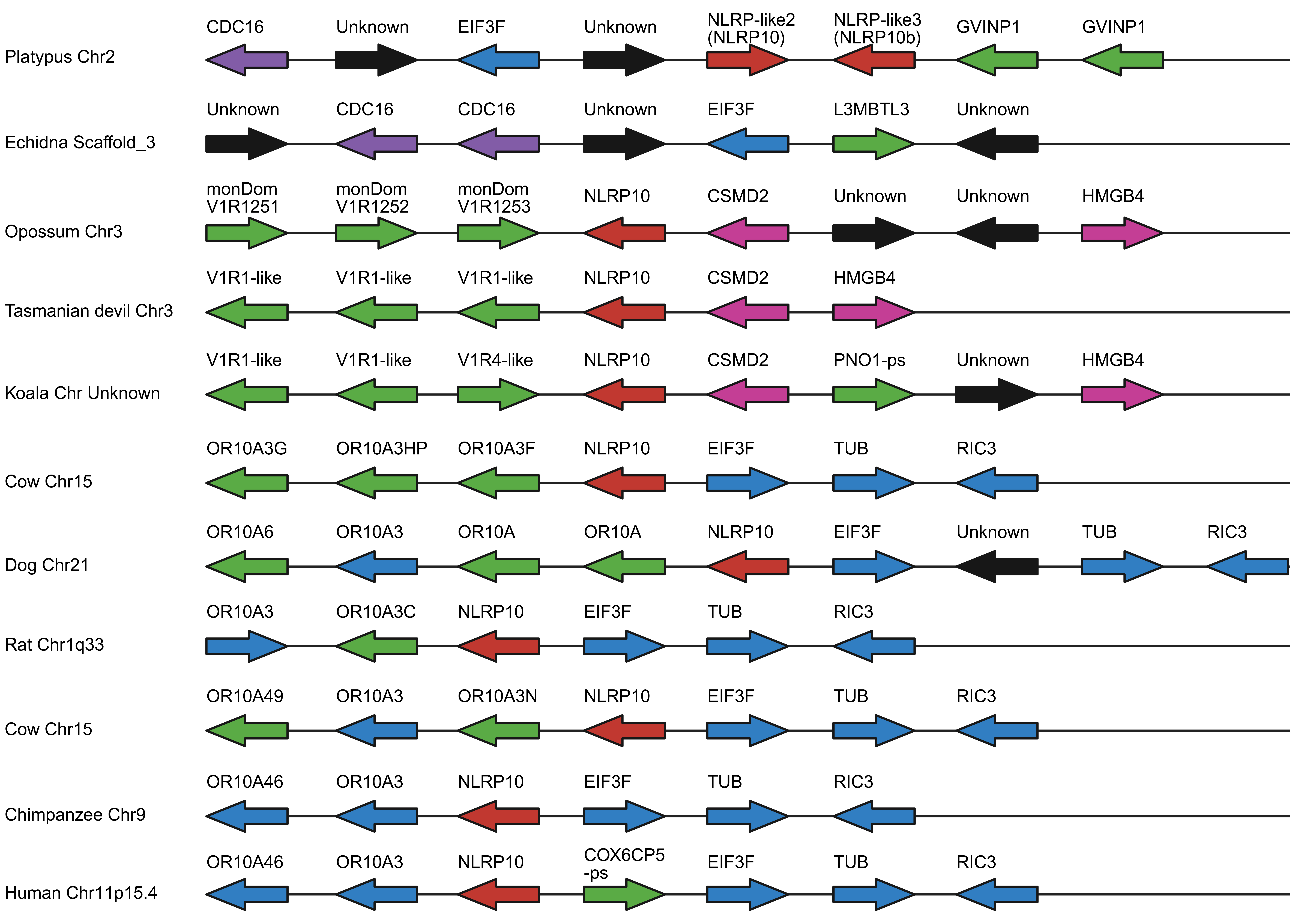


Online Resource 14 Chromosomal arrangements of *Nlrp10* for multiple mammalian species. A duplication of *Nlrp10* has occurred in the platypus while echidna lacks a *Nlrp10* homologue. The predicted identities in brackets are based on phylogenetic results. Arrows indicate orientation. Red arrows are genes of interest, blue, purple and pink arrows are conserved between species, green arrows are species specific and black arrows are putative genes with no apparent orthologue. -ps at the end of a gene name indicates a pseudogene. Distances not to scale


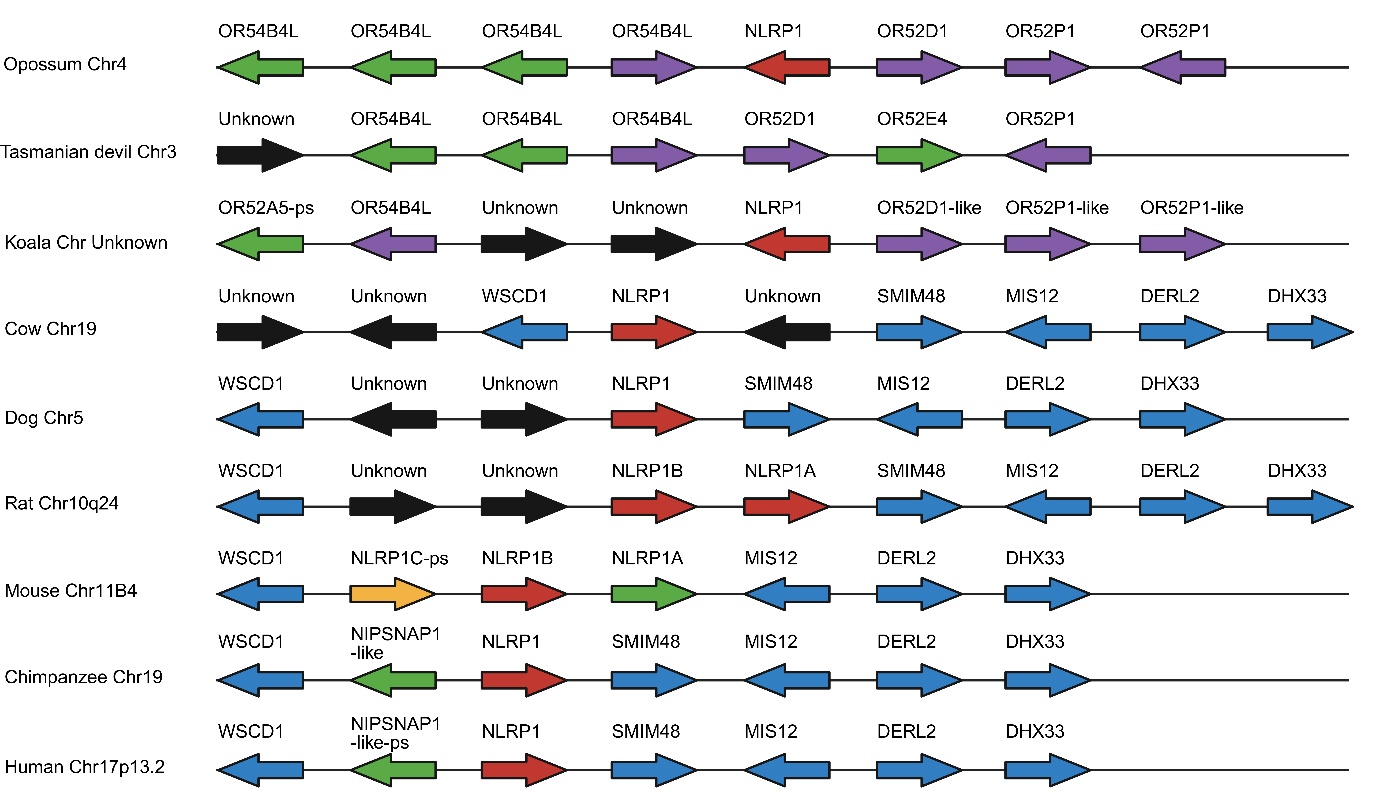


Online Resource 15 Chromosomal arrangements of *Nlrp1* for multiple therian species. Tasmanian devil lacks a *Nlrp1* homologue. Arrows indicate orientation. Red arrows are genes of interest, blue and purple arrows are conserved between species, green arrows are species specific and black arrows are putative genes with no apparent orthologue. The yellow arrow represents a Nlrp-like pseudogene. -ps at the end of a gene name indicates a pseudogene. Distances not to scale


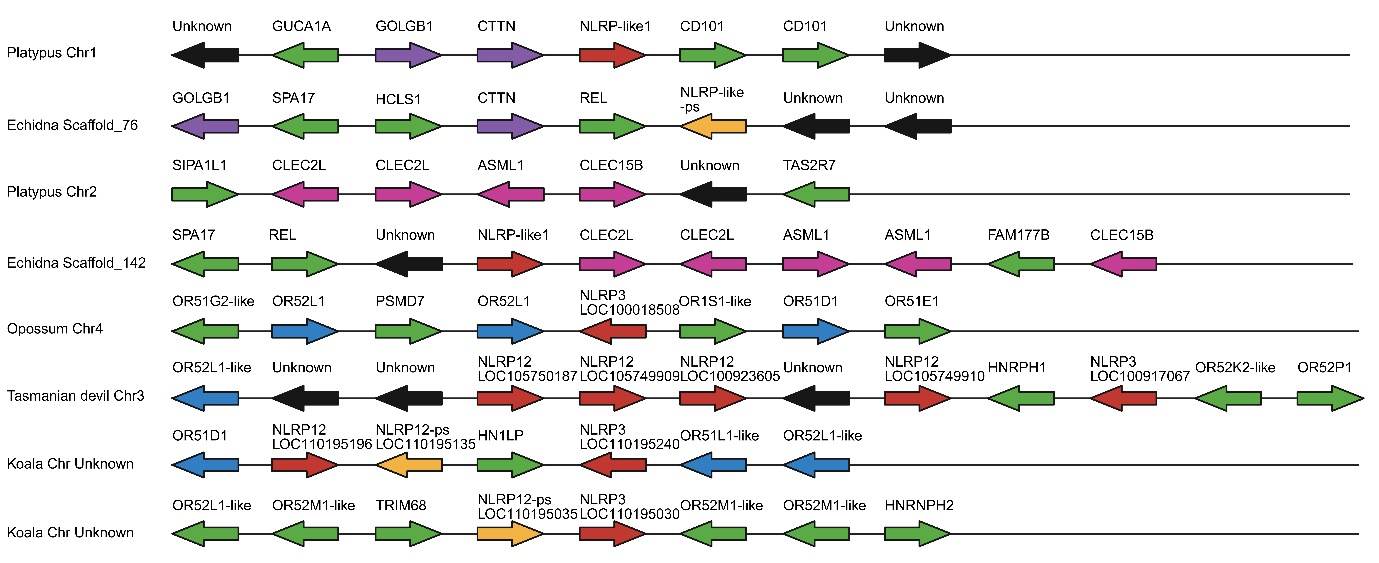


Online Resource 16 Chromosomal arrangements of genes associated with the *Nlrp-like* cluster including those from platypus, echidna, opossum, Tasmanian devil and koala. Platypus and echidna *Nlrp-like1* are homologous but share no synteny. Marsupial gene identities are as listed on NCBI. Arrows indicate orientation. Red arrows are genes of interest, blue, purple and pink arrows are conserved between species, green arrows are species specific and black arrows are putative genes with no apparent orthologue. Yellow arrows are Nlrp-like pseudogenes. -ps at the end of a gene name indicates a pseudogene. Distances not to scale





Online Resource 17 Phylogenetic analysis of NLRP and NLRP-like genes. Predicted protein sequences from multiple mammalian species and chicken as an outgroup were aligned in MEGAX using MUSCLE. The marsupial rNlrp and Nlrp-like sequences are named according to the NCBI identification. a) The Maximum likelihood tree was generated using the Jones-Taylor-Thornton model (+Freq) with 1000 bootstrap replicates. Gamma distribution was used to model the evolutionary rate differences among sites with 0.61% of sites evolutionarily invariant (+*I*). The tree is drawn to scale and represents the tree with the highest log likelihood (-52,231.02). 95% partial deletion was used to remove all positions with lower coverage. Chicken NLRP3 is used as the root. Bootstrap values lower than 50% are not shown. b) The Neighbour-joining phylogenetic tree was generated with 1000 bootstrap replicates. The tree is drawn to scale. The p-distance method was used to compute evolutionary distances representing the number of amino acid substitutions per site. Pairwise deletion was used to remove all ambiguous positions for each sequence pair. The optimal tree with the sum of branch length = 18.897 is shown. Chicken NLRP3 is used as the root. Bootstrap values lower than 50% are not shown. Monotreme sequences are marked by red (echidna) or blue (platypus) circles. Marsupial sequences are marked by purple (opossum), green (Tasmanian devil) or brown (koala) triangles


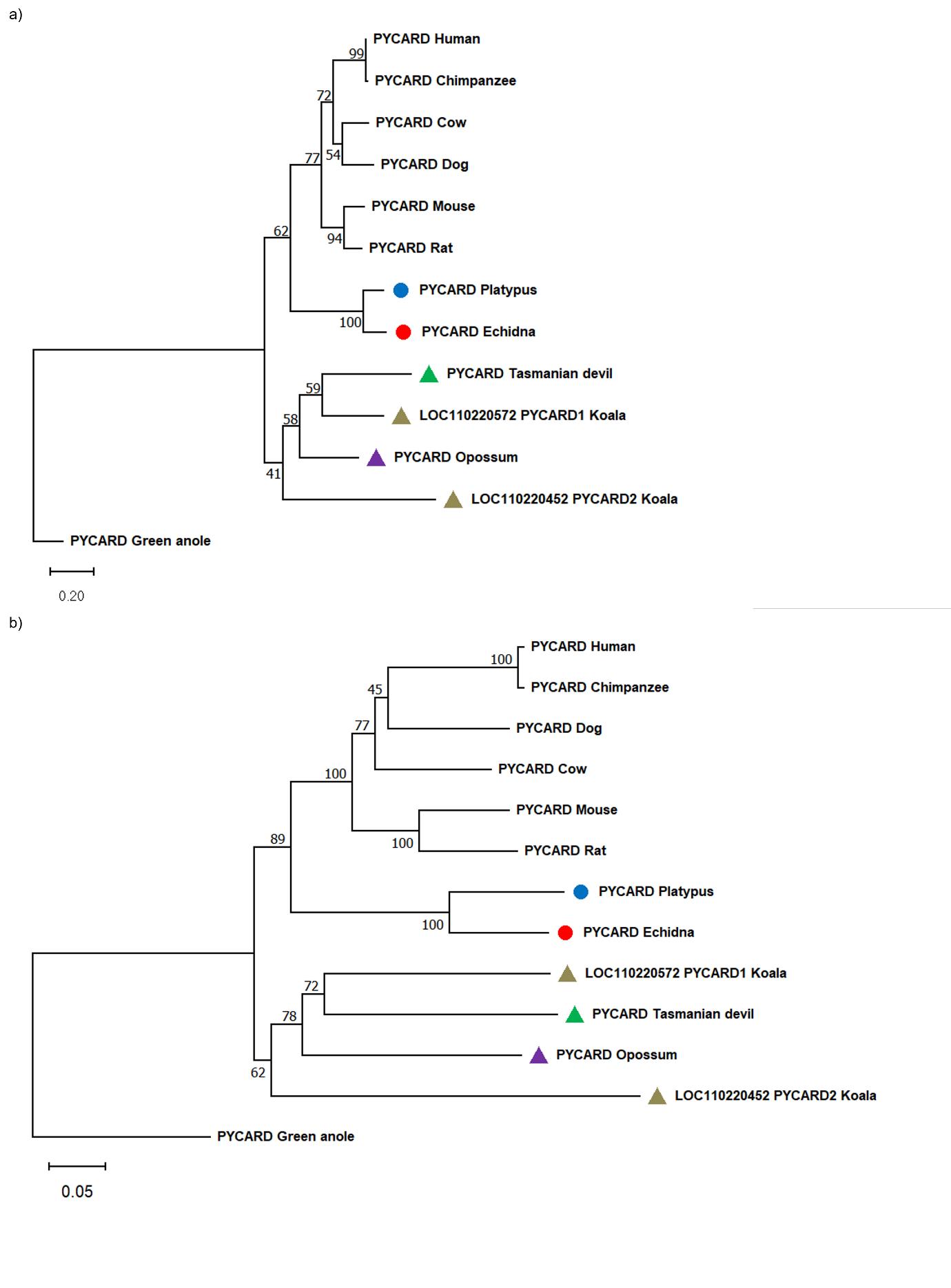


Online Resource 18 Phylogenetic analysis of Pycard. Predicted protein sequences from multiple mammalian species and green anole lizard as an outgroup were aligned in MEGAX using MUSCLE. a) The Maximum likelihood tree was generated using the Jones-Taylor-Thornton model with 1000 bootstrap replicates. Gamma distribution was used to model the evolutionary rate differences among sites. The tree is drawn to scale and represents the tree with the highest log likelihood (-2,870.68). 95% partial deletion was used to remove all positions with lower coverage. Green anole lizard Pycard is used as the root. b) The Neighbour-joining phylogenetic tree was generated with 1000 bootstrap replicates. The tree is drawn to scale. The p-distance method was used to compute evolutionary distances representing the number of amino acid substitutions per site. Pairwise deletion was used to remove all ambiguous positions for each sequence pair. The optimal tree with the sum of branch length = 2.344 is shown. Green anole lizard Pycard is used as the root. Monotreme sequences are marked by red (echidna) or blue (platypus) circles. Marsupial sequences are marked by purple (opossum), green (Tasmanian devil) or brown (koala) triangles


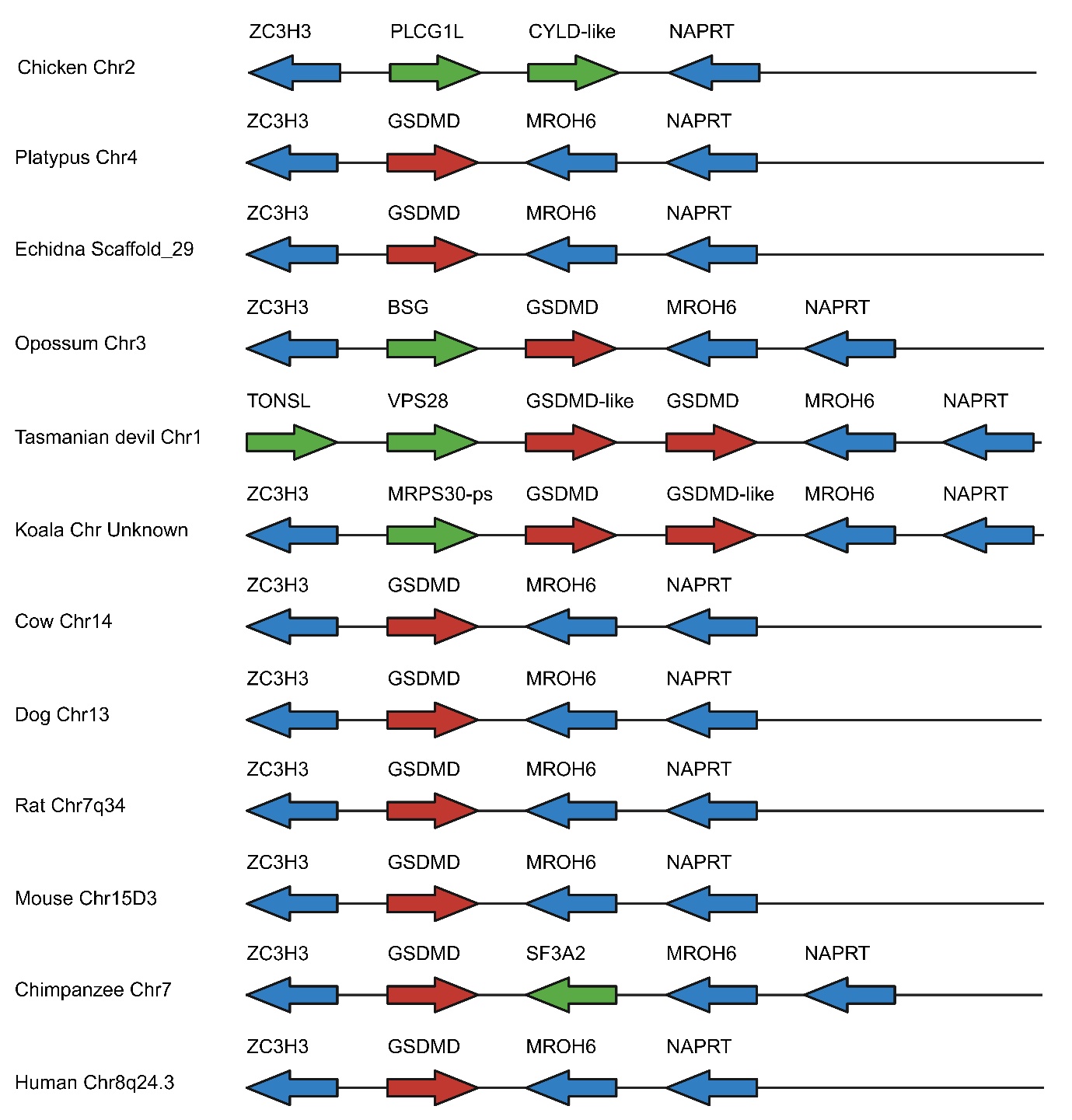


Online Resource 19 *Gsdmd* and flanking genes in multiple mammalian species with chicken as a non-mammalian outgroup. Arrows indicate orientation. The red arrows represent the genes of interest, blue arrows represent conserved gene synteny and green arrows are species specific. Distances not to scale


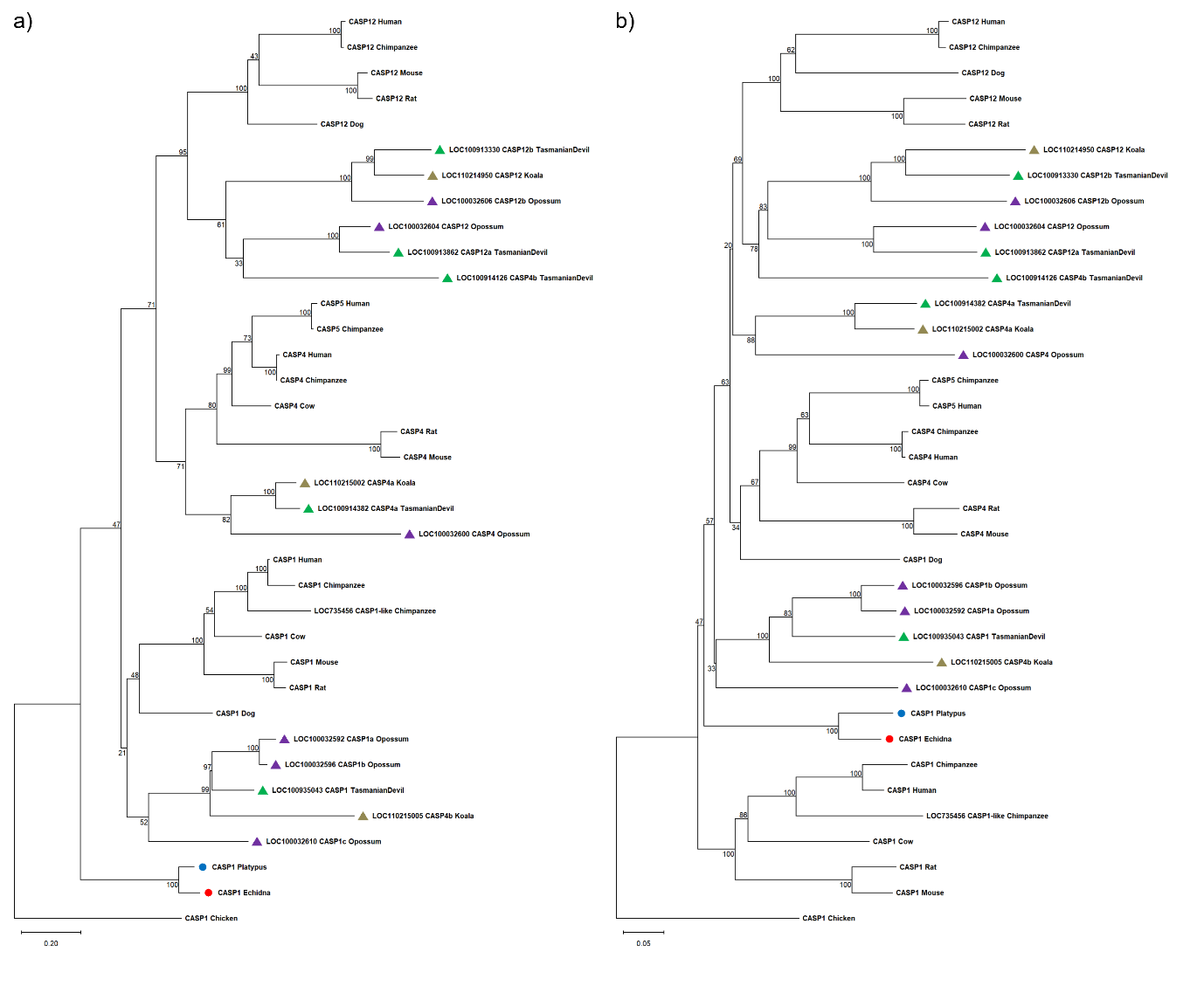


Online Resource 20 Phylogenetic analysis of inflammatory caspases in multiple mammalian species with chicken as a non-mammalian outgroup. Predicted protein sequences were aligned in MEGAX using MUSCLE. Marsupial caspases are listed according to their NCBI identification. a) The Maximum likelihood tree was generated using the Jones-Taylor-Thornton model with 1000 bootstrap replicates. Gamma distribution was used to model the evolutionary rate differences among sites with 7.37% of sites evolutionarily invariant (+*I*). The tree is drawn to scale and represents the tree with the highest log likelihood (-12,887.96). 95% partial deletion was used to remove all positions with lower coverage. Chicken Caspase-1 is used as the root. b) The Neighbour-joining phylogenetic tree was generated with 1000 bootstrap replicates. The tree is drawn to scale. The p-distance method was used to compute evolutionary distances representing the number of amino acid substitutions per site. Pairwise deletion was used to remove all ambiguous positions for each sequence pair. The optimal tree with the sum of branch length = 5.966 is shown. Chicken Caspase-1 is used as the root. Monotreme sequences are marked by red (echidna) or blue (platypus) circles. Marsupial sequences are marked by purple (opossum), green (Tasmanian devil) or brown (koala) triangles


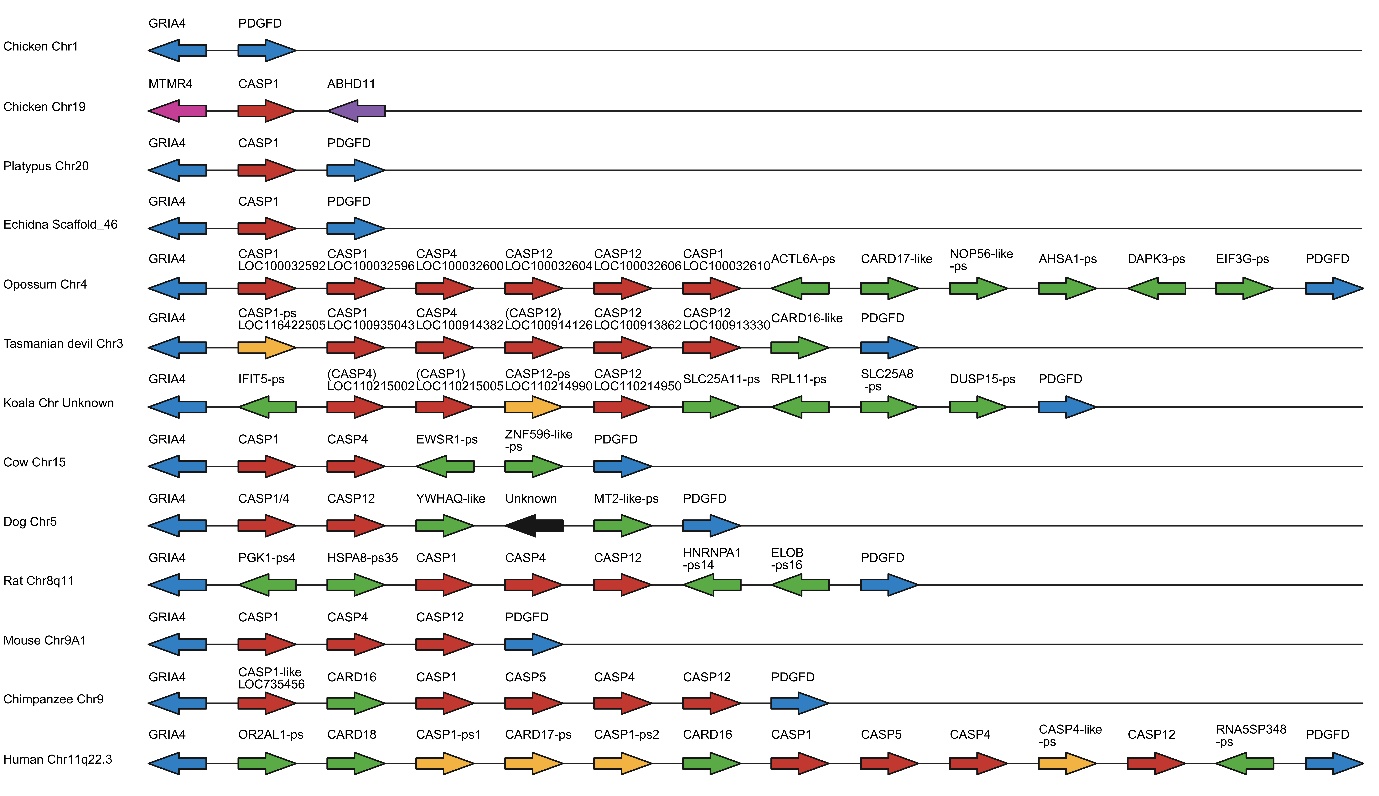


Online Resource 21 Expansion of the Caspase-1 subfamily cluster. The mammalian *Caspase-1* is located in a different gene environment to chicken *Caspase-1*. Opossum has a large expansion of the gene cluster with duplications of *Caspase-1* and *Caspase-12* as does Tasmanian devil with duplications to *Caspase-12*. Koala has a partial *Caspase-12* and a rearrangement of the gene order with the *Caspase-4* homologue before *Caspase-1*. Koala and Tasmanian devil genes with names in brackets indicate the predicted identity based on phylogenetic trees. Dog has a fusion of *Caspases -1* and *-4* while cow has a *Caspase-12* deletion. Chimpanzee contains a duplication of *Caspase-1*. Human has a *Caspase-4-like* pseudogene and two *Caspase-1* pseudogenes. *Caspase-1* is located on chicken chr19 and platypus chr20. The mammalian *Caspase-1* syntenic region can be found preserved on chicken chr1 without a caspase homologue. The chicken Caspase-1 syntenic block can be found with disrupted synteny on platypus chr17 with the upstream and downstream syntenic genes forming separate blocks (not shown). Red arrows are genes of interest, blue arrows are conserved between species, green arrows are species specific and black arrows are putative genes with no apparent orthologue. Purple and pink arrows represent the chicken *Caspase-1* flanking genes. Yellow arrows represent Caspase pseudogenes. -ps at the end of a gene name indicates a pseudogene. Distances not to scale

**REFERENCES**

Ahrens S, Zelenay S, Sancho D, Hanč P, Kjær S, Feest C, Fletcher G, Durkin C, Postigo A, Skehel M, Batista F, Thompson B, Way M, Reis e Sousa C, Schulz O (2012) F-Actin Is an Evolutionarily Conserved Damage-Associated Molecular Pattern Recognized by DNGR-1, a Receptor for Dead Cells. Immunity 36:635

Alshehri OM, Montague S, Watson S, Carter P, Sarker N, Manne BK, Miller JL, Herr AB, Pollitt AY, O'Callaghan CA, Kunapuli S, Arman M, Hughes CE, Watson SP (2015) Activation of glycoprotein VI (GPVI) and C-type lectin-like receptor-2 (CLEC-2) underlies platelet activation by diesel exhaust particles and other charged/hydrophobic ligands. Biochem J 468:459

Ashouri JF, Lo W-L, Nguyen TTT, Shen L, Weiss A (2022) ZAP70, too little, too much can lead to autoimmunity. Immunological Reviews 307:145

Au-Yeung BB, Shah NH, Shen L, Weiss A (2018) ZAP-70 in Signaling, Biology, and Disease. Annual Review of Immunology 36:127

Blot L, Bzioueche H, Heim M, Cheli Y, Sormani L, Verhoeyen E, Stella A, Burlet-Schiltz O, Rocchi S, Larue L, Rombouts Y, Lepenies B, Passeron T, Tulic MK (2025) CLEC12B Regulates Melanocyte Immunity and Homeostasis in the Skin through the Signal Transducer and Activator of Transcription 1/IRF1 Axis. J Invest Dermatol 145:2007

Boyden ED, Dietrich WF (2006) Nalp1b controls mouse macrophage susceptibility to anthrax lethal toxin. Nature genetics 38:240

Cho Y, Cao Z, Luo X, Tian JJ, Hukkanen RR, Hussien R, Cancilla B, Chowdhury P, Li F, Ma S, LaGory EL, Schroeder M, Dusenberry A, Marshall L, Hawkins J, van Lookeren Campagne M, Zhou Y (2024) NLRP10 maintains epidermal homeostasis by promoting keratinocyte survival and P63-dependent differentiation and barrier function. Cell Death & Disease 15:759

Christou Charita M, Pearce Andrew C, Watson Aleksandra A, Mistry Anita R, Pollitt Alice Y, Fenton-May Angharad E, Johnson Louise A, Jackson David G, Watson Steve P, O'Callaghan Chris A (2008) Renal cells activate the platelet receptor CLEC-2 through podoplanin. Biochemical Journal 411:133

Digby Z, Tourlomousis P, Rooney J, Boyle JP, Bibo-Verdugo B, Pickering RJ, Webster SJ, Monie TP, Hopkins LJ, Kayagaki N, Salvesen GS, Warming S, Weinert L, Bryant CE (2021) Evolutionary loss of inflammasomes in the Carnivora and implications for the carriage of zoonotic infections. Cell Rep 36:109614

Duenez-Guzman EA, Haig D (2014) The Evolution of Reproduction-Related NLRP Genes. JOURNAL OF MOLECULAR EVOLUTION 78:194

Gao S, Wake H, Sakaguchi M, Wang D, Takahashi Y, Teshigawara K, Zhong H, Mori S, Liu K, Takahashi H, Nishibori M (2020) Histidine-Rich Glycoprotein Inhibits High-Mobility Group Box-1-Mediated Pathways in Vascular Endothelial Cells through CLEC-1A. iScience 23:101180

Gorfu G, Cirelli KM, Melo MB, Mayer-Barber K, Crown D, Koller BH, Masters S, Sher A, Leppla SH, Moayeri M, Saeij JP, Grigg ME (2014) Dual role for inflammasome sensors NLRP1 and NLRP3 in murine resistance to Toxoplasma gondii. mBio 5

Hara H, Seregin SS, Yang D, Fukase K, Chamaillard M, Alnemri ES, Inohara N, Chen GY, Núñez G (2018) The NLRP6 inflammasome recognizes lipoteichoic acid and regulates gram-positive pathogen infection. Cell 175:1651

Kaifu T, Yabe R, Maruhashi T, Chung SH, Tateno H, Fujikado N, Hirabayashi J, Iwakura Y (2021) DCIR and its ligand asialo-biantennary N-glycan regulate DC function and osteoclastogenesis. J Exp Med 218

Levinsohn JL, Newman ZL, Hellmich KA, Fattah R, Getz MA, Liu S, Sastalla I, Leppla SH, Moayeri M (2012) Anthrax lethal factor cleavage of Nlrp1 is required for activation of the inflammasome. PLoS Pathog 8:e1002638

Levy M, Thaiss CA, Zeevi D, Dohnalová L, Zilberman-Schapira G, Mahdi JA, David E, Savidor A, Korem T, Herzig Y (2015) Microbiota-modulated metabolites shape the intestinal microenvironment by regulating NLRP6 inflammasome signaling. Cell 163:1428

Negishi I, Motoyama N, Nakayama K-i, Nakayama K, Senju S, Hatakeyama S, Zhang Q, Chan AC, Loh DY (1995) Essential role for ZAP-70 in both positive and negative selection of thymocytes. Nature 376:435

Neumann K, Castiñeiras-Vilariño M, Höckendorf U, Hannesschläger N, Lemeer S, Kupka D, Meyermann S, Lech M, Anders H-J, Kuster B, Busch Dirk H, Gewies A, Naumann R, Groß O, Ruland J (2014) Clec12a Is an Inhibitory Receptor for Uric Acid Crystals that Regulates Inflammation in Response to Cell Death. Immunity 40:389

Prochnicki T, Vasconcelos MB, Robinson KS, Mangan MS, De Graaf D, Shkarina K, Lovotti M, Standke L, Kaiser R, Stahl R (2023) Mitochondrial damage activates the NLRP10 inflammasome. Nature immunology 24:595

Richard M, Thibault N, Veilleux P, Gareau-Pagé G, Beaulieu AD (2006) Granulocyte macrophage-colony stimulating factor reduces the affinity of SHP-2 for the ITIM of CLECSF6 in neutrophils: A new mechanism of action for SHP-2. Molecular Immunology 43:1716

Shen C, Lu A, Xie WJ, Ruan J, Negro R, Egelman EH, Fu T-M, Wu H (2019) Molecular mechanism for NLRP6 inflammasome assembly and activation. Proceedings of the National Academy of Sciences 116:2052

Shih HH, Zhang S, Cao W, Hahn A, Wang J, Paulsen JE, Harnish DC (2009) CRP is a novel ligand for the oxidized LDL receptor LOX-1. American Journal of Physiology-Heart and Circulatory Physiology 296:H1643

Stappers MHT, Clark AE, Aimanianda V, Bidula S, Reid DM, Asamaphan P, Hardison SE, Dambuza IM, Valsecchi I, Kerscher B, Plato A, Wallace CA, Yuecel R, Hebecker B, da Glória Teixeira Sousa M, Cunha C, Liu Y, Feizi T, Brakhage AA, Kwon-Chung KJ, Gow NAR, Zanda M, Piras M, Zanato C, Jaeger M, Netea MG, van de Veerdonk FL, Lacerda JF, Campos A, Carvalho A, Willment JA, Latgé J-P, Brown GD (2018) Recognition of DHN-melanin by a C-type lectin receptor is required for immunity to Aspergillus. Nature 555:382

Suzuki S, Mimuro H, Kim M, Ogawa M, Ashida H, Toyotome T, Franchi L, Suzuki M, Sanada T, Suzuki T, Tsutsui H, Núñez G, Sasakawa C (2014) Shigella IpaH7.8 E3 ubiquitin ligase targets glomulin and activates inflammasomes to demolish macrophages. Proc Natl Acad Sci U S A 111:E4254

Tian X, Pascal G, Monget P (2009) Evolution and functional divergence of NLRP genes in mammalian reproductive systems. BMC Evolutionary Biology 9:202

Wang P, Zhu S, Yang L, Cui S, Pan W, Jackson R, Zheng Y, Rongvaux A, Sun Q, Yang G (2015) Nlrp6 regulates intestinal antiviral innate immunity. Science 350:826

Wang Y, Hasegawa M, Imamura R, Kinoshita T, Kondo C, Konaka K, Suda T (2004) PYNOD, a novel Apaf‐1/CED4‐like protein is an inhibitor of ASC and caspase‐1. International Immunology 16:777

Zaki MH, Vogel P, Malireddi RKS, Body-Malapel M, Anand Paras K, Bertin J, Green Douglas R, Lamkanfi M, Kanneganti T-D (2011) The NOD-Like Receptor NLRP12 Attenuates Colon Inflammation and Tumorigenesis. Cancer Cell 20:649

Zhang L, Duan M, Pu X, Zheng H, Ning X, Tu Y, Xu C, Zhang D, Liu C, Xie J (2024) GroEL triggers NLRP3 inflammasome activation through the TLR/NF-κB p-p65 axis in human periodontal ligament stem cells. Acta Biochim Biophys Sin (Shanghai) 56:1340

Zhao X, Shen Y, Hu W, Chen J, Wu T, Sun X, Yu J, Wu T, Chen W (2015) DCIR negatively regulates CpG-ODN-induced IL-1β and IL-6 production. Molecular Immunology 68:641

Zhu H, Lee C, Zhang D, Wu W, Wang L, Fang X, Xu X, Song D, Xie J, Ren S, Gu J (2013) Surface-associated GroEL facilitates the adhesion of Escherichia coli to macrophages through lectin-like oxidized low-density lipoprotein receptor-1. Microbes and Infection 15:172
